# The oncoprotein MUC1 facilitates breast cancer progression by promoting Pink1-dependent mitophagy via ATAD3A destabilization

**DOI:** 10.1038/s41419-022-05345-z

**Published:** 2022-10-26

**Authors:** Quanfu Li, Yunkai Chu, Shengze Li, Liping Yu, Huayun Deng, Chunhua Liao, Xiaodong Liao, Chihyu Yang, Min Qi, Jinke Cheng, Guoqiang Chen, Lei Huang

**Affiliations:** 1 Department of Histoembryology, Genetics and Developmental Biology, Key Laboratory of Cell Differentiation and Apoptosis of Chinese Ministry of Education, Shanghai Key Laboratory of Reproductive Medicine, Shanghai Jiao Tong University School of Medicine, Shanghai, China; 2 Department of Anesthesiology, Shanghai Pulmonary Hospital, Tongji University School of Medicine, Shanghai, China; 3 Department of Plastic Surgery, Xiangya Hospital, Central South University, Changsha, China

**Keywords:** Breast cancer, Mitophagy

## Abstract

Mitophagy is a vital process that controls mitochondria quality, dysregulation of which can promote cancer. Oncoprotein mucin 1 (MUC1) targets mitochondria to attenuate drug-induced apoptosis. However, little is known about whether and how MUC1 contributes to mitochondrial homeostasis in cancer cells. We identified a novel role of MUC1 in promoting mitophagy. Increased mitophagy is coupled with the translocation of MUC1 to mitochondria, where MUC1 interacts with and induces degradation of ATPase family AAA domain-containing 3A (ATAD3A), resulting in protection of PTEN-induced kinase 1 (Pink1) from ATAD3A-mediated cleavage. Interestingly, MUC1-induced mitophagy is associated with increased oncogenicity of cancer cells. Similarly, inhibition of mitophagy significantly suppresses MUC1-induced cancer cell activity in vitro and in vivo. Consistently, MUC1 and ATAD3A protein levels present an inverse relationship in tumor tissues of breast cancer patients. Our data validate that MUC1/ATAD3A/Pink1 axis-mediated mitophagy constitutes a novel mechanism for maintaining the malignancy of cancer cells, providing a novel therapeutic approach for MUC1-positive cancers.

## Introduction

Mitophagy is a selective form of autophagy that eliminates damaged mitochondria or excessive mitochondria [1]. Given its crucial role in cellular homeostasis, defective mitophagy has been proposed to contribute to various diseases, including cancer [2]. Deficiency in mitophagy leads to the accumulation of abnormal mitochondria, which cause metabolic disturbances and reactive oxygen species (ROS) accumulation and consequently initiates tumorigenesis [3]. However, during cancer progression, enhanced mitophagy may also benefit tumor growth. Mitophagy facilitates the recycling of metabolic precursors that are important for cell survival [4]. Mitophagy has also been shown to protect against drug-induced cell death [5] and stimulate cancer stemness [6]. However, how mitophagy is regulated in cancer cells is not completely understood.

Mucin1 (MUC1) is a heterodimeric transmembrane protein that serves as a protective barrier against microbes, toxins, mechanical forces, and other forms of stress. MUC1 also contributes to repairing damaged epithelia by activating epigenetic reprogramming, epithelial-mesenchymal transition (EMT), and stemness to maintain epithelial homeostasis [7]. MUC1 consists of two subunits: an N-terminal highly glycosylated subunit and an oncogenic MUC1 C-terminal (MUC1-C). MUC1-C consists of a 58-aa extracellular domain, a 28-aa transmembrane region, and an intrinsically disordered 72-aa cytoplasmic tail [8]. MUC1 is aberrantly overexpressed in numerous human carcinomas [9], including in greater than 90% of breast cancer cases [10]. MUC1-C involves multiple hallmarks of cancer cells, including integrating inflammation and proliferation with EMT, epigenetic reprogramming, chromatin remodeling, and the promotion of plasticity [11]. MUC1 enhances cancer stemness through the activation of pluripotency networks [12]. MUC1 also localizes to the mitochondrial outer membrane, maintaining mitochondrial membrane integrity to attenuate the drug-induced release of mitochondrial apoptogenic factors and activation of apoptosis [13]. Our previous work reported that MUC1 induces acquired chemotherapy resistance and stimulates cancer stem cell enrichment in cervical cancer [14, 15]. Here, we investigated the effect of MUC1 on mitophagy, dissected the potential underlying molecular mechanism, and uncovered the biological function of MUC1-related mitophagy in cancer.

## Results

### MUC1 promotes mitophagy in multiple cancer cell lines

**Fig. 1:**
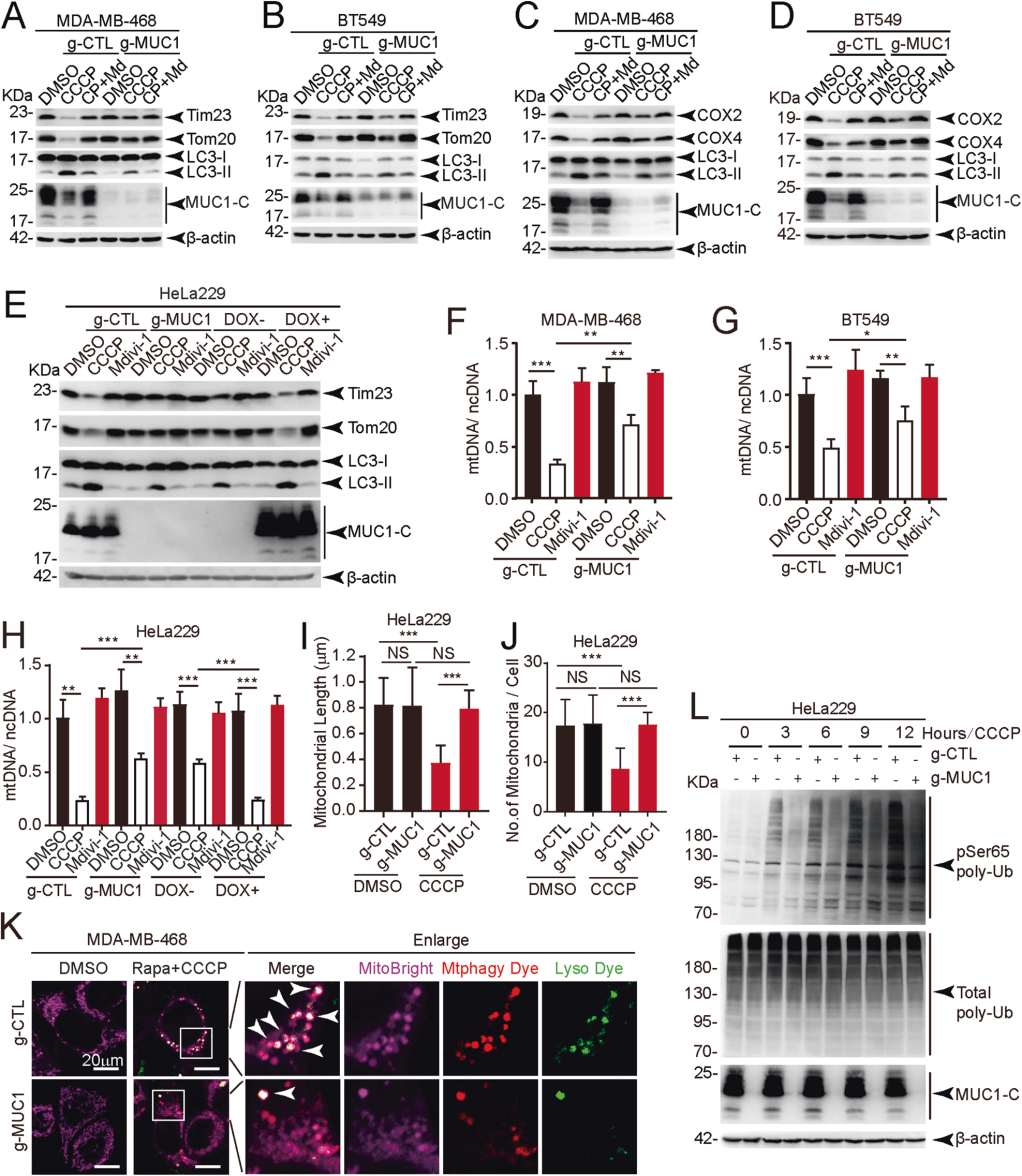
MUC1 enhances CCCP-induced mitophagy. **A**, **C** Western blotting was performed to detect proteins as indicated in MDA-MB-468/g-CTL and MDA-MB-468/g-MUC1 cells treated with DMSO, 5 μM CCCP (24 h) or 5 μM CCCP (24 h) + 10 μM Mdivi-1 (24 h). Quantification of mitochondrial proteins was calculated. **B**, **D** Western blotting was performed to detect proteins as indicated in BT549/g-CTL and BT549/g-MUC1 cells treated with DMSO, 10 μM CCCP (24 h) or 10 μM CCCP (24 h) + 10 μM Mdivi-1 (24 h). Quantification of mitochondrial proteins was calculated. **E** After treatment with DMSO, 30 μM CCCP (24 h) or 30 μM Mdivi-1 (24 h), western blotting was performed to detect proteins with the indicated antibodies in HeLa229/g-CTL, HeLa229/g-MUC1, and HeLa229/g-MUC1 cells treated with 1 μg/ml DOX for 48 h to induce MUC1 expression (DOX+) and without DOX treatment (DOX−) as a negative control. Quantification of mitochondrial proteins was calculated. **F** The ratio of mtDNA (COX1) to ncDNA (B2M) (mtDNA/ncDNA) in MDA-MB-468/g-CTL and MDA-MB-468/g-MUC1 cells treated with DMSO, 5 μM CCCP (24 h) or 10 μM Mdivi-1 (24 h). **G** The ratio of mtDNA (COX1) to ncDNA (B2M) (mtDNA/ncDNA) in BT549/g-CTL and BT549/g-MUC1 cells treated with DMSO, 10 μM CCCP (24 h) or 10 μM Mdivi-1 (24 h). **H** The ratio of mtDNA (COX1) to ncDNA (B2M) (mtDNA/ncDNA) in HeLa229/g-CTL, HeLa229/g-MUC1, and HeLa229/g-MUC1 cells treated with 1 μg/ml DOX for 48 h to induce MUC1 expression (DOX+) and without DOX treatment (DOX−) as a negative control. HeLa229 cells were treated with DMSO, 30 μM CCCP (24 h) or 30 μM Mdivi-1 (24 h). **I**, **J** Average length of mitochondria per cell (**I**) and the number of mitochondria per cell (**J**) in TEM micrographs from Fig. S1I. More than 16 cells in each graph were analyzed. **K** Representative fluorescence images from (Fig. S1J) with Mtphagy Dye (Red), Lysosome Dye (Green), and MitoBright (Purple) staining in MDA-MB-468 cells stimulated with DMSO, 5 μM CCCP and 500 nM rapamycin. The arrow indicates colocalization pots. Rapa: Rapamycin. Bars: 20 μm. **L** HeLa229/g-CTL and HeLa229/g-MUC1 cells were treated with 30 μM CCCP for the indicated times, and western blotting was performed to detect pSer65-Ub and total-Ub. The data represent the mean ± SD from three independent experiments. Differences between linked groups were evaluated by a two-tailed Student’s *t* test. *P < 0.05, **P < 0.01; ***P < 0.001; NS not significant.

To examine the possible effects of MUC1 on mitophagy, we first detected the mitochondrial protein levels of Tom20 (located in the outer membrane) [16] and Tim23 (located in the inner membrane) [17] in cells treated with either the mitophagy activator carbonyl cyanide 3-chlorophenylhydrazone (CCCP) or the mitophagy inhibitor mitochondrial division inhibitor 1 (Mdivi-1). Interestingly, CCCP alone but not combined treatment induced a marked reduction in the mitochondrial proteins Tom20 and Tim23 in MUC1-proficient cells (MDA-MB-468/g-CTL) but not in MUC1-deficient cells (MDA-MB-468/g-MUC1) (Figs. 1A, S1A). Similar results were obtained in BT549/g-CTL cells and BT549/g-MUC1 cells (Figs. 1B, S1B). Parallel assessment was further performed on proteins encoded by mitochondrial DNA (mtDNA), including cytochrome-c-oxidase 2 (COX2) [18] and cytochrome-c-oxidase 4 (COX4) [19]. Consistently, the reduction in COX2 and COX4 protien levels was considerably enhanced by CCCP treatment in MDA-MB-468/g-CTL and BT549/g-CTL cells but not in MDA-MB-468/g-MUC1 and BT549/g-MUC1 cells (Figs. 1C, 1D, S1C, S1D). This result was also confirmed in a cervical cancer HeLa229 cell line, a subline of HeLa that is easy to culture and transfect [20]. DOX-induced MUC1 in HeLa229/g-MUC1 cells rescued the downregulation of Tom20 and Tim23 upon CCCP treatment (Figs. 1E, S1E). This phenomenon was corroborated by the MUC1 dose-dependent degradation of the mitochondrial proteins Tom20 and Tim23 (Fig. S1F). We also found that a higher concentration of either CCCP or O/A (another activator of mitophagy) led to a more obvious reduction in Tom20 and Tim23 in MUC1-proficient cells (HeLa229/g-CTL) than in MUC1-deficient cells (HeLa229/g-MUC1) (Fig. S1G, S1H), suggesting mitochondrial protein degradation in a dose-dependent manner. These results suggest a role for MUC1 in CCCP-induced mitochondrial turnover.

To further detect mitophagy activity, mtDNA abundance was measured. Upon CCCP treatment, mtDNA was considerably diminished in MDA-MB-468/g-CTL (Fig. 1F), BT549/g-CTL (Fig. 1G), and HeLa229/g-CTL (Fig. 1H) cells. We then quantified the average length of mitochondria and the number of mitochondria using transmission electron microscopy (TEM) images [21]. Although the average length of mitochondria and the number of mitochondria were comparable between HeLa229/g-CTL and HeLa229/g-MUC1 cells in the control group, CCCP treatment induced a significant reduction in the average length (Figs. 1I, S1I) and number (Figs. 1J, S1I) of mitochondria in HeLa229/g-CTL cells, suggesting a higher level of mitophagy in MUC1-expressing cells. A Mitophagy Detection Kit was applied to further verify mitophagy. Compared with DMSO treatment, CCCP significantly increased signature colocalization of Mtphagy dye (Red) with Lysosome dye (Green) in MDA-MB-468/g-CTL cells upon the induction of autophagy by rapamycin [22] (Figs. 1K, S1J, S1K). This phenomenon was further supported by an immunofluorescent staining assay. With chloroquine (CQ) treatment to inhibit autolysosomes [23], CCCP resulted in a considerable increase in the colocalization of Tom20 and LC3B in MUC1-expressing cells (Figs. S1L, S1M). Given that phosphorylation of ubiquitin at Ser65 (pSer65-Ub) by Pink1 is the initiation step of mitophagy [24], we assessed whether MUC1 influences pSer65-Ub. Indeed, compared with MUC1-deficient cells, in MUC1-expressing HeLa229 cells, MUC1 dramatically augmented pSer65-Ub after CCCP treatment over time (Fig. 1L). These results collectively reveal that MUC1 promotes mitophagy in cancer cells.

### MUC1 interacts with the mitochondrial protein ATAD3A

**Fig. 2:**
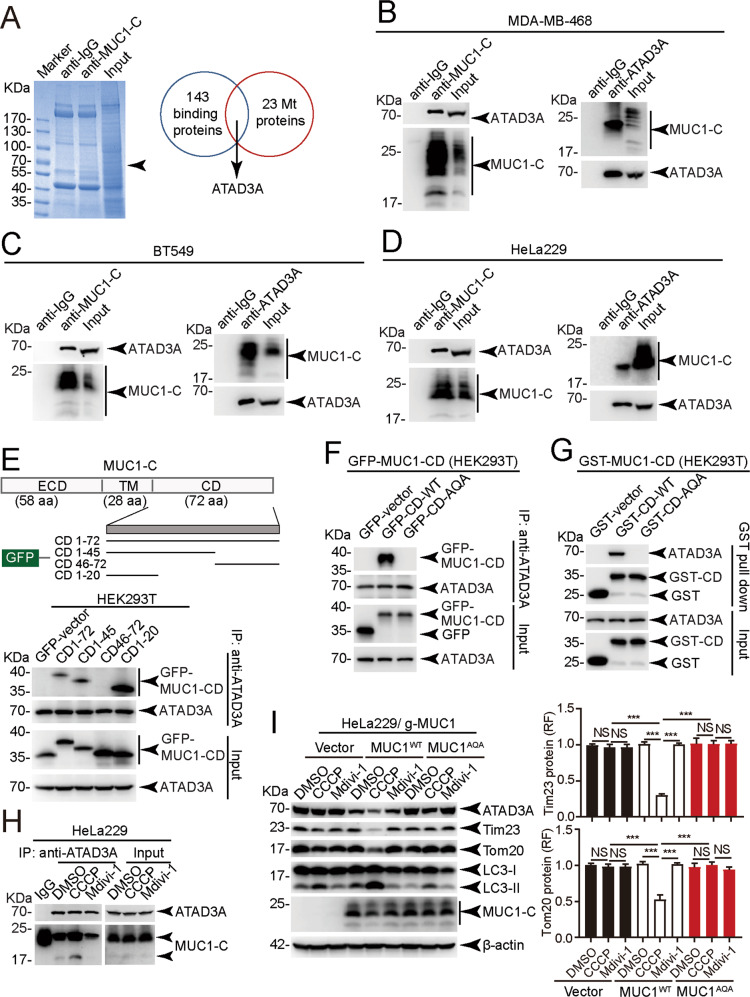
MUC1 interacts with ATAD3A through the CQC motif, which is necessary for mitophagy. **A** Proteins of HeLa229 cells precipitated by anti-MUC1-C or IgG antibody were analyzed by mass spectrometry and co-IP. The proteins from the co-IP assay were separated by SDS-PAGE and stained with Coomassie brilliant blue. Screening of proteins localized in mitochondria, including ATAD3A. **B**–**D** Co-IP assay to detect the interaction of endogenous MUC1 and ATAD3A. Anti-MUC1-C immunoprecipitates from MDA-MB-468 (**B**), BT549 (**C**) and HeLa229 (**D**) cells were analyzed by western blotting with the indicated antibodies (left). Anti-ATAD3A immunoprecipitates from MDA-MB-468 (**B**), BT549 (**C**), and HeLa229 (**D**) cells were analyzed by western blotting with the indicated antibodies (right). **E** The schematic shows GFP fused with different truncations of MUC1-CD (upper). HEK293T cells were transfected with GFP-MUC1-CD truncated mutant plasmids, subjected to a co-IP assay with an anti-ATAD3A antibody, and then analyzed by western blotting using anti-GFP and anti-ATAD3A antibodies. **F** Co-IP assay of MUC1-CD and ATAD3A in HEK293T cells. HEK293T cells were cotransfected with ATAD3A and pEGFP-MUC1-CD or pEGFP-MUC1-CD-AQA. Cell lysates were subjected to denatured co-IP. **G** Purified recombinant GST, GST-MUC1-CD, or GST-MUC1-AQA was incubated with ATAD3A. The precipitated beads were detected by western blot using anti-ATAD3A and anti-GST antibodies. **H** Co-IP of endogenous MUC1 and ATAD3A in HeLa229 cells treated with 30 μM CCCP (2 h) or 30 μM Mdivi-1 (2 h). After IP with IgG or anti-ATAD3A, the immunoprecipitates were analyzed with anti-MUC1-C and anti-ATAD3A antibodies. **I** HeLa229/g-MUC1 cells were transfected with vector, MUC1-CD-WT and MUC1-CD-AQA plasmids and then treated with DMSO, 30 μM CCCP (24 h) or 30 μM Mdivi-1 (24 h). Western blotting was performed using antibodies as indicated. Quantification of mitochondrial proteins was calculated. The data represent the mean ± SD from three independent experiments. Differences between linked groups were evaluated using a two-tailed Student’s *t* test. *P < 0.05, **P < 0.01; ***P < 0.001; NS not significant.

To understand the mechanism underlying MUC1 mediating mitophagy, we searched for interacting partners of MUC1 by performing coimmunoprecipitation (co-IP)-coupled liquid chromatography with tandem mass spectrometry (LC-MS/MS) (Fig. 2A). Among the 143 proteins identified (Table S1), there were 23 mitochondrial proteins. We focused on the ATPase family AAA domain-containing 3A (ATAD3A) protein, which was enriched among all identified proteins and is essential for mitophagy regulation [25]. The interaction between MUC1 and ATAD3A was confirmed by co-IP in MDA-MB-468 (Fig. 2B), BT549 (Fig. 2C) and HeLa229 cells (Fig. 2D). We further mapped the domain of MUC1 responsible for binding with ATAD3A using various truncated mutants of MUC1 in HEK293T cells (Fig. 2E upper). The results indicated that MUC1-CD1-20 contained a binding site for ATAD3A (Fig. 2E lower). Consistent with a previous report that the CQC motif in MUC1-CD1-20 is involved in MUC1-C targeting mitochondria [26], the MUC1-CD-AQA mutant abolished the interaction of MUC1 and ATAD3A both in vivo (Fig. 2F) and in vitro (Fig. 2G). Moreover, we found that CCCP treatment enhanced the interaction between MUC1 and ATAD3A (Fig. 2H). Ectopic expression of MUC1 wild-type (MUC1^WT^) but not AQA-mutant (MUC1^AQA^) restored CCCP-induced mitophagy (Fig. 2I). These results demonstrate that the MUC1-CQC motif is required for the binding of MUC1 and ATAD3A, which abolishes MUC1-mediated mitophagy if mutated.

### MUC1 promotes ATAD3A degradation via the ubiquitin‒proteasome pathway

**Fig. 3:**
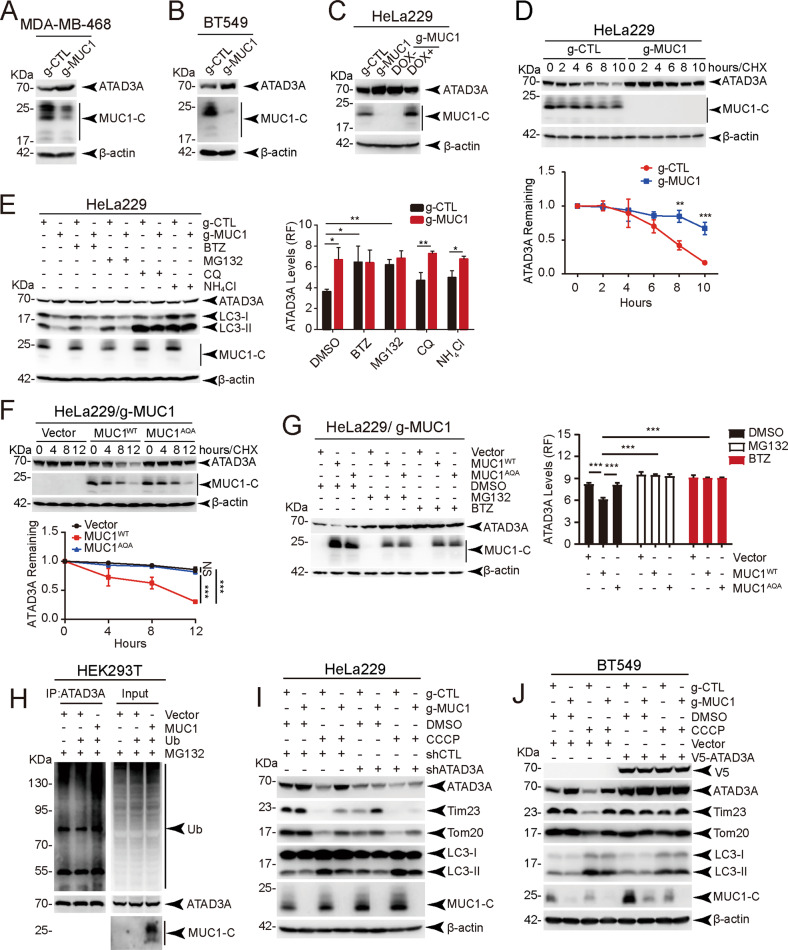
MUC1 downregulates ATAD3A, and ATAD3A inhibits MUC1-mediated mitophagy. **A**, **B** Western blotting was performed to detect ATAD3A protein levels in MDA-MB-468 cells (**A**) and BT549 cells (**B**). **C** Western blotting was performed to detect ATAD3A protein levels in HeLa229/g-CTL, HeLa229/g-MUC1, and HeLa229/g-MUC1 cells treated with 1 μg/ml DOX for 48 h to induce MUC1 expression (DOX+) and without DOX treatment (DOX−) as a negative control. **D** After treatment with CHX (10 μg/ml) for different times, the degradation rates of ATAD3A proteins were measured in HeLa229/g-CTL and HeLa229/g-MUC1 cells. The relative quantification of ATAD3A protein levels after CHX treatments was analyzed, and the half-life curve of ATAD3A was generated. **E** Western blotting was performed to detect ATAD3A protein levels in HeLa229/g-CTL and HeLa229/g-MUC1 cells after treatment with proteasome (50 nM BTZ (8 h) or 10 μM MG132 (8 h)) or autolysosome (100 μM CQ (8 h) or 20 mM NH_4_Cl (8 h)) inhibitors, respectively. The relative quantification of ATAD3A protein levels was analyzed. **F** HeLa229/g-MUC1 cells were transfected with vector, MUC1-CD-WT or MUC1-CD-AQA plasmids and then treated with CHX (10 μg/ml) for 0, 4, 8, and 12 h. Western blotting was performed. The relative quantification of ATAD3A protein levels after CHX treatments was analyzed, and the half-life curve of ATAD3A was generated. **G** HeLa229/g-MUC1 cells were transfected with vector, MUC1-CD-WT, and MUC1-CD-AQA plasmids for 48 h and then treated with 50 nM BTZ (8 h) or 10 μM MG132 (8 h). Western blotting was performed to detect ATAD3A protein levels. The relative quantification of ATAD3A protein levels was analyzed. **H** HEK293T cells were transfected with His-Ub and vector or MUC1-HA plasmids and then treated with 10 μM MG132 for 8 h. After IP with anti-ATAD3A antibody, the immunoprecipitants were analyzed with the indicated antibodies. **I** HeLa229/g-CTL and HeLa229/g-MUC1 cells were transfected with shCTL and shATAD3A lentiviral vectors and then treated with DMSO or 30 μM CCCP (24 h). Western blotting was performed using antibodies as indicated. Quantification of mitochondrial proteins was calculated. **J** BT549/g-CTL and BT549/g-MUC1 cells were transfected with vector and ATAD3A-V5 lentiviral vectors and then treated with DMSO or 10 μM CCCP (24 h). Western blotting was performed using the indicated antibodies. Quantification of mitochondrial proteins was calculated. The data represent the mean ± SD from three independent experiments. Differences between linked groups were evaluated by a two-tailed Student’s *t* test. *P < 0.05, **P < 0.01; ***P < 0.001; NS not significant.

To investigate the significance of the MUC1-ATAD3A interaction, we detected ATAD3A protein levels. The results showed that MUC1 notably decreased ATAD3A protein levels (Fig. 3A–C) but had little, if any, effect on ATAD3A mRNA levels (Fig. S3A). These data suggest that MUC1 might regulate ATAD3A protein stability. Therefore, we measured the protein half-life of ATAD3A. The half-life of ATAD3A was remarkably shortened in MUC1-expressing cells compared with MUC1-depleted cells (Fig. 3D), suggesting that MUC1 promotes ATAD3A protein degradation. To investigate how MUC1 stimulated ATAD3A turnover, proteasome (bortezomib (BTZ) and MG132) or lysosome (CQ and NH_4_Cl) inhibitors were employed [27, 28]. The data showed that only proteasome inhibitors blocked ATAD3A turnover (Fig. 3E). MUC1-triggered ATAD3A degradation was confirmed by re-expression of wild-type MUC1 but abolished by AQA-mutant MUC1 in HeLa229/g-MUC1 cells (Fig. 3F). ATAD3A degradation mediated by MUC1 overexpression could also be blocked by BTZ and MG132 (Fig. 3G). Consistently, we observed MUC1-induced ATAD3A ubiquitination (Fig. 3H). These data demonstrate that MUC1 induces ATAD3A turnover via the ubiquitin‒proteasome pathway.

We next explored how ATAD3A contributes to MUC1-mediated mitophagy by using loss-of-function and gain-of-function strategies. Partial depletion of ATAD3A using short hairpin RNA considerably increased CCCP-induced mitophagy in HeLa229/g-CTL cells (Figs. 3I, S3B). In contrast, ectopic expression of ATAD3A blocked CCCP-induced mitophagy in BT549/g-CTL cells (Figs. 3J, S3C). These results suggest a role for ATAD3A in the suppression of MUC1-mediated mitophagy. Interestingly, we found that 2 h of treatment upregulated MUC1 levels in both HeLa229 and MDA-MB-468 cells. However, CCCP treatment for 6, 12, and 24 h decreased MUC1 levels in MDA-MB-468 cells (Fig. S3D) but had minimal effect on MUC1 levels in HeLa229 cells (Fig. S3E).

### MUC1 is translocated to mitochondria and protects Pink1 from ATAD3A-mediated cleavage upon CCCP treatment

**Fig. 4:**
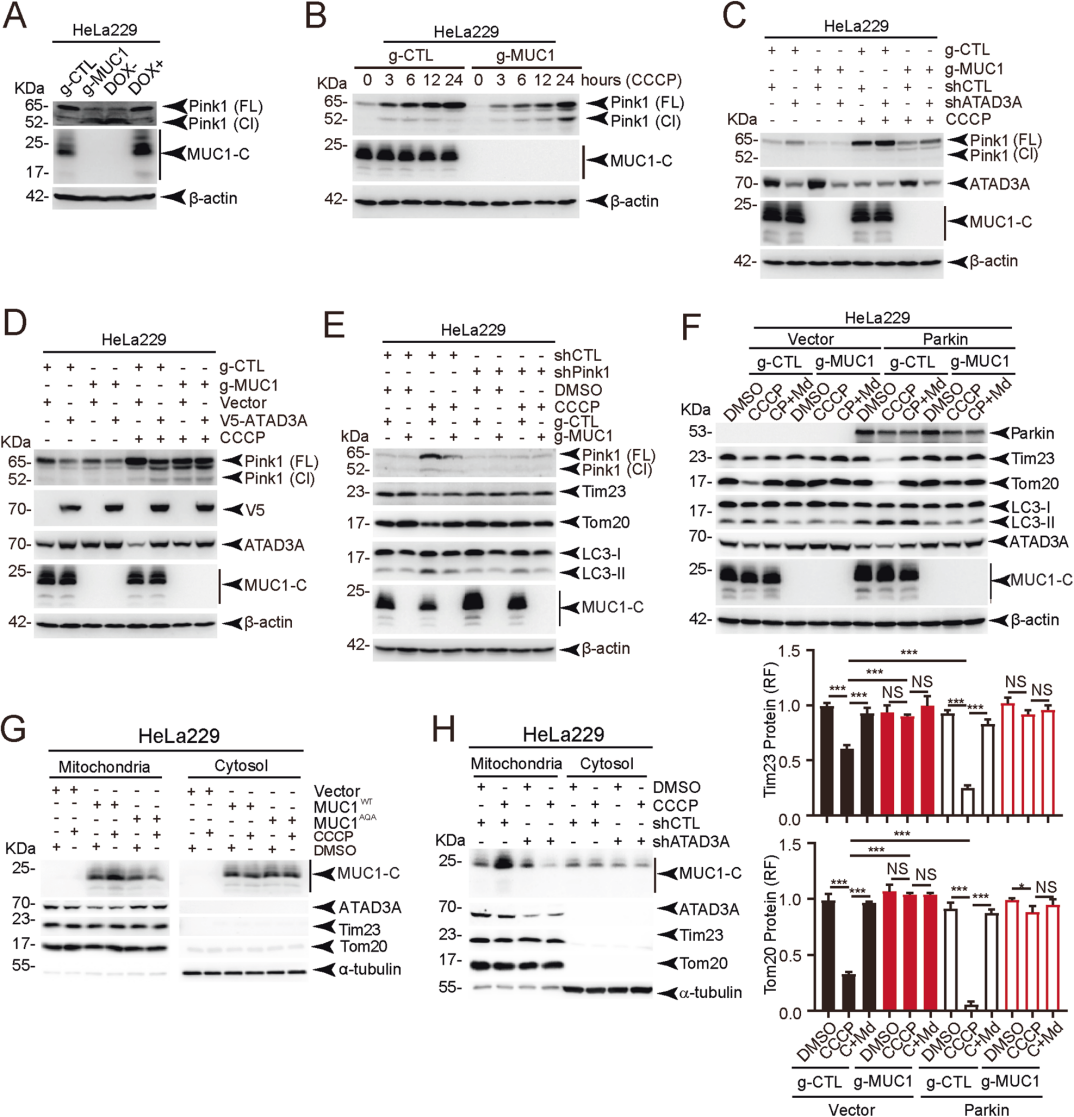
MUC1 translocates to mitochondria to protect Pink1 from ATAD3A-mediated cleavage. **A** Western blotting was performed to detect Pink1 protein levels in four cell lines, HeLa229/g-CTL, HeLa229/g-MUC1, and HeLa229/g-MUC1, treated with 1 μg/ml DOX for 48 h to induce MUC1 expression (DOX+) and without DOX treatment (DOX−) as a negative control. Pink1 (FL): full-length Pink1; Pink1 (Cl): cleaved Pink1. **B** HeLa229/g-CTL and HeLa229/g-MUC1 cells were treated with 30 μM CCCP for the indicated times. Western blotting was then performed to detect Pink1 protein levels. **C** HeLa229/g-CTL and HeLa229/g-MUC1 cells were transfected with shCTL and shATAD3A lentiviral vectors for 48 h and then treated with or without 30 μM CCCP. Western blotting was performed to detect Pink1 protein levels. **D** HeLa229/g-CTL and HeLa229/g-MUC1 cells were transfected with vector and ATAD3A-V5 lentiviral vectors for 48 h and then treated with or without 30 μM CCCP. Western blotting was performed to detect Pink1 protein levels. **E** HeLa229/g-CTL and HeLa229/g-MUC1 cells were transfected with shCTL and shPink1 lentiviral vectors and then treated with DMSO or 30 μM CCCP (24 h). Western blotting was performed using antibodies as indicated. The relative quantification of protein levels was analyzed. **F** HeLa229/g-CTL and HeLa229/g-MUC1 cells were transfected with vector and Parkin-HA plasmids for 48 h and then treated with DMSO, 30 μM CCCP (24 h) or a combination of 30 μM CCCP (24 h) and 30 μM Mdivi-1 (24 h). Western blotting was performed using the indicated antibodies. The relative quantification of protein levels was analyzed. CP + Md: CCCP + Mdivi-1. **G** HeLa229/g-MUC1 cells were transfected with vector, MUC1-CD-WT and MUC1-CD-AQA plasmids and then treated with DMSO and 30 μM CCCP (2 h). Sediment from the mitochondrial fraction was detected by western blot. Tim23, Tom20, and ATAD3A served as mitochondrial markers, and α-tubulin was used as a cytosolic marker. **H** HeLa229 cells were transfected with shCTL and shATAD3A lentiviral vectors and treated with DMSO and 30 μM CCCP (2 h). Sediment from the mitochondrial fraction was detected by western blot. Tim23, Tom20, and ATAD3A were used as mitochondrial markers, and α-tubulin was used as a cytosolic marker. The data represent the mean ± SD from three independent experiments. Differences between linked groups were evaluated by a two-tailed Student’s *t* test. *P < 0.05, **P < 0.01; ***P < 0.001; NS not significant.

Previous studies have shown that the accumulation of full-length PTEN-induced kinase 1 (Pink1) on the mitochondrial outer membrane is critical for the induction of mitophagy [29]. Moreover, ATAD3A suppresses mitophagy by promoting Pink1 cleavage [25]. We hypothesized that the MUC1-triggered downregulation of ATAD3A expression might protect Pink1 from cleavage. Indeed, the data showed that MUC1 expression was associated with an increase in full-length Pink1 and a reduction in cleaved Pink1 upon CCCP exposure (Fig. 4A, B). As predicted, the results showed that shATAD3A resulted in a more obvious accumulation of Pink1 in MUC1-positive cells (g-CTL) (Fig. 4C), and enforced ATAD3A diminished full-length Pink1 in g-CTL cells (Fig. 4D). To test whether MUC1-mediated mitophagy depended on Pink1, shPink1 was applied. The results showed that the depletion of Pink1 considerably reduced CCCP-induced mitophagy in HeLa229/g-CTL cells (Figs. 4E, S4A). These results together indicate that MUC1 induces the accumulation of full-length Pink1 by downregulating ATAD3A, resulting in the activation of mitophagy. To further determine whether MUC1 promoted mitophagy through the Pink1-Parkin pathway, we detected Parkin expression in all three cancer cell lines, and HEK293T cells served as a positive control [30]. Minimal, if any, Parkin expression was detected in MDA-MB-468, BT549, and HeLa229 cells (Fig. S4B). However, ectopic Parkin expression enhanced CCCP-induced mitophagy in MUC1-expressing HeLa229 cells (Fig. 4F). These data suggest that MUC1-mediated mitophagy is Pink1-dependent but Parkin-independent. We also obtained similar results with O/A-induced mitophagy (Fig. S4C). Consistently, CCCP stimulated the translocation of MUC1^WT^ to the mitochondria, which was restrained upon MUC1^AQA^ (Figs. 4G, S4D). Notably, silencing ATAD3A also blocked CCCP-induced MUC1 distribution to mitochondria (Figs. 4H, S4E). These results illustrate that MUC1 translocates to mitochondria and protects Pink1 from ATAD3A-mediated cleavage.

### MUC1-mediated mitophagy triggers tumor cell malignancy

**Fig. 5:**
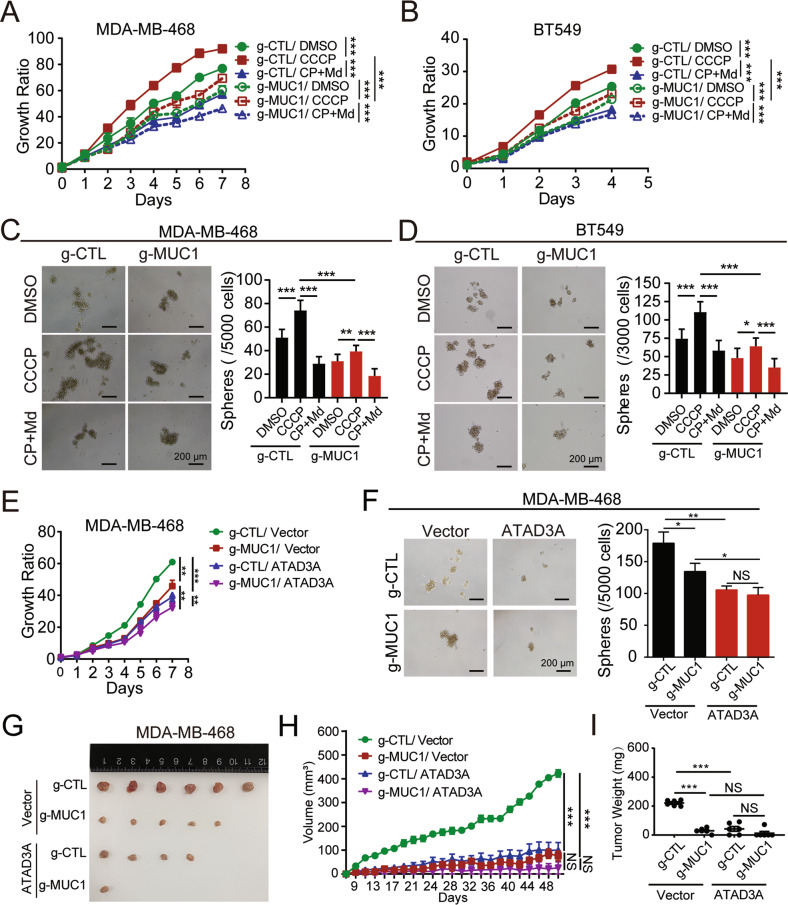
MUC1-mediated mitophagy promotes tumor cell malignancy. **A** MDA-MB-468/g-CTL and MDA-MB-468/g-MUC1 cells were pretreated with DMSO, 5 μM CCCP (2 h), or a combination of 5 μM CCCP (2 h) and 10 μM Mdivi-1 (6 h) before being plated in medium with 10 μM Mdivi-1, and then the cells were subjected to a viability assay. CP + Md: CCCP + Mdivi-1. **B** BT549/g-CTL and BT549/g-MUC1 cells were pretreated with DMSO, 10 μM CCCP (2 h) or a combination of 10 μM CCCP (2 h) and 10 μM Mdivi-1 (6 h) before being plated in medium with 10 μM Mdivi-1, and then the cells were subjected to a viability assay. **C** MDA-MB-468/g-CTL and MDA-MB-468/g-MUC1 cells were pretreated with DMSO, 5 μM CCCP (2 h) or a combination of 5 μM CCCP (2 h) and 10 μM Mdivi-1 (6 h) and then plated in medium with 10 μM Mdivi-1. Cells were subjected to the mammosphere formation assay. Representative images are shown. The number of mammospheres > 50 μm in diameter from MDA-MB-468/g-CTL and MDA-MB-468/g-MUC1 cells was calculated. Bars: 200 μm. (**D**) BT549/g-CTL and BT549/g-MUC1 cells were pretreated with DMSO, 10 μM CCCP (2 h) or a combination of 10 μM CCCP (2 h) and 10 μM Mdivi-1 (6 h) and then plated in medium with 10 μM Mdivi-1. Cells were subjected to the mammosphere formation assay. Representative images are shown. The number of mammospheres >50 μm in diameter from BT549/g-CTL and BT549/g-MUC1 cells was calculated. Bars: 200 μm. **E** MDA-MB-468/g-CTL and MDA-MB-468/g-MUC1 cells were transfected with vector and ATAD3A-V5 lentiviral vectors. Then, the cells were subjected to a viability assay. **F** MDA-MB-468/g-CTL and MDA-MB-468/g-MUC1 cells were transfected with vector and ATAD3A-V5 lentivirus. Then, cells were subjected to the mammosphere formation assay. Representative images are shown. Bars: 200 μm. The number of mammospheres >50 μm in diameter was calculated. The data represent the mean ± SD from three independent experiments. **G**–**I** Tumor sizes (**G**, **H**) and weights (**I**) in mice injected with the indicated MDA-MB-468 cells (g-CTL/g-CTL-ATAD3A, g-MUC1/g-MUC1-ATAD3A) were compared. Differences between linked groups were evaluated by a two-tailed Student’s *t* test. *P < 0.05, **P < 0.01; ***P < 0.001; NS not significant.

Multiple studies have reported that mitophagy stimulation favors tumor cell malignancy [31]. To assess the function of MUC1-induced mitophagy, we stimulated cells for 2 h with CCCP to activate mitophagy and examined breast cancer cell proliferation. To observe the effect of CCCP on mitochondrial function, we detected the ROS levels in MDA-MB-468 cells and BT549 cells treated with CCCP for 2 or 24 h. The results showed that CCCP treatment for a short time (2 h) slightly increased ROS levels, but CCCP treatment for a long time (24 h) more significantly elevated ROS levels in MUC1 knockdown cells than in MUC1-expressing cells, suggesting the role of MUC1 in ROS clearance (Fig. S5A, S5B). We also found that short-term CCCP exposure considerably increased the proliferation of MUC1-proficient cells in either MDA-MB-468 (Fig. 5A) or BT549 cells (Fig. 5B). Conversely, cotreatment with CCCP and Mdivi-1 blocked CCCP-stimulated proliferation of cancer cells (Fig. 5A, B). We also found that mammosphere formation was drastically augmented by CCCP and markedly diminished by Mdivi-1 in MDA-MB-468/g-CTL (Fig. 5C) and BT549/g-CTL cells (Fig. 5D). These results demonstrate that MUC1 promotes mitophagy and thereby enhances tumor cell proliferation and mammosphere formation. We further assessed the impact of ATAD3A on the oncogenic properties of breast cancer cells. ATAD3A overexpression was associated with a substantial decrease in cell proliferation and mammosphere formation not only in MDA-MB-468 cells (Fig. 5E, F) but also in BT549 cells (Figs. S5C and S5D) and delayed tumor growth without affecting mouse body weight (Figs. 5G–I, S5E). These results together suggest that MUC1-induced oncogenic activity through mitophagy was hindered by ATAD3A.

### Cosuppression of MUC1 and mitophagy sufficiently impedes tumor growth

**Fig. 6:**
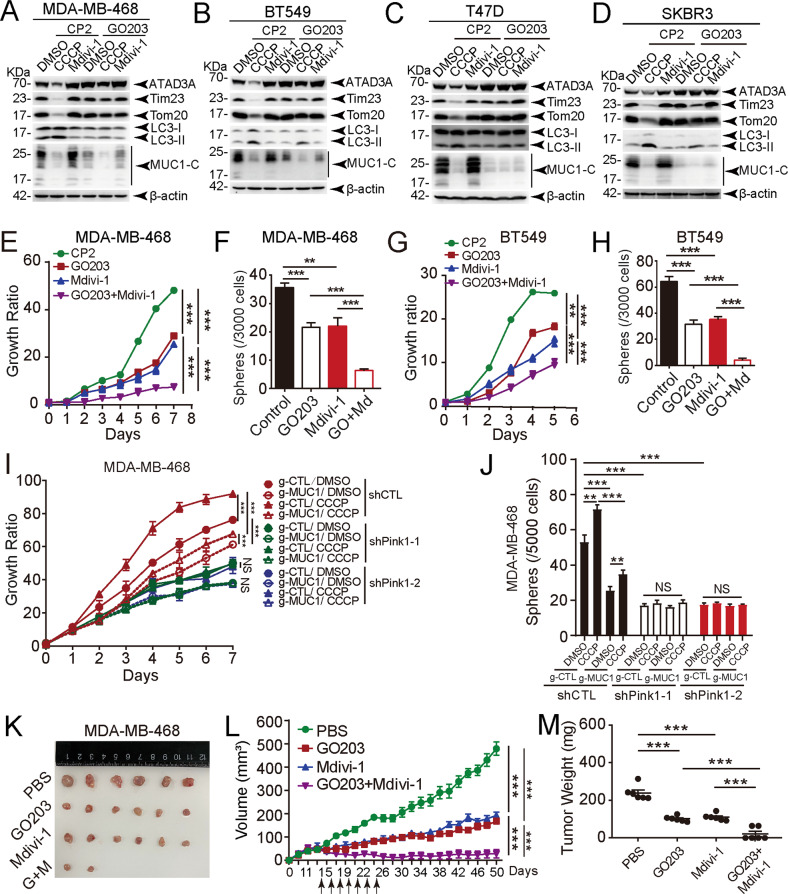
Targeting MUC1-mitophagy signaling inhibits tumor malignancy. **A** MDA-MB-468 cells were treated with 5 μM CP2 (48 h) and 5 μM GO203 (48 h) and then treated with DMSO, 5 μM CCCP (24 h) or 10 μM Mdivi-1 (24 h). Western blotting was performed to detect proteins as indicated. Mitochondrial proteins were quantified. GO-203, a cell-penetrating peptide containing a polyArg cell transduction domain linked to the CQCRRKN sequence ([R]9-CQCRRKN; all D-amino acids), can bind directly to the MUC1-C cytoplasmic domain. In contrast, CP2 is a GO203 analog (CP2: [R]9-AQARRKN) that is ineffective in inhibiting MUC1-C. CP2 acted as a control for GO203. **B** BT549 cells were treated with 5 μM CP2 (48 h) and 5 μM GO203 (48 h) and then treated with DMSO, 10 μM CCCP (24 h) or 10 μM Mdivi-1 (24 h). Western blotting was performed to detect proteins as indicated. Mitochondrial proteins were quantified. **C** T47D cells were treated with 10 μM CP2 (48 h) and 10 μM GO203 (48 h) and then treated with DMSO, 10 μM CCCP (24 h), or 10 μM Mdivi-1 (24 h). Western blotting was performed to detect proteins as indicated. Mitochondrial proteins were quantified. **D** SKBR3 cells were treated with 10 μM CP2 (48 h) and 10 μM GO203 (48 h) and then treated with DMSO, 10 μM CCCP (24 h) or 10 μM Mdivi-1 (24 h). Western blotting was performed to detect proteins as indicated. Quantification of mitochondrial proteins was calculated. **E** The cell viability assay was performed in MDA-MB-468 cells treated with 5 μM GO203 and 10 μM Mdivi-1 alone or in combination. **F** The mammosphere formation assay was performed in MDA-MB-468 cells treated with 5 μM GO203 and 10 μM Mdivi-1 alone or in combination. The number of mammospheres was calculated. GO + Md: GO203 + Mdivi-1. **G** The cell viability assay was performed in BT549 cells treated with 5 μM GO203 and 10 μM Mdivi-1 alone or in combination. **H** A mammosphere formation assay was performed in BT549 cells treated with 5 μM. GO203 and 10 μM Mdivi-1 alone or in combination. The number of mammospheres was calculated. **I** MDA-MB-468/g-CTL and MDA-MB-468/g-MUC1 cells were transfected with shCTL, shPink1-1, and shPink1-2 lentiviral vectors for 48 h and then treated with DMSO and 5 μM CCCP (2 h). The cell viability assay was performed. **J** MDA-MB-468/g-CTL and MDA-MB-468/g-MUC1 cells were transfected with shCTL, shPink1-1, and shPink1-2 lentiviral vector for 48 h, then treated with DMSO and 5 μM CCCP (2 h). Cells were subjected to the mammospheres formation assay. The number of mammospheres >50 μm in diameter was calculated. The data represent mean ± SD from three independent experiments. **K**–**M** Tumor sizes (**K**, **L**) and weights (**M**) were compared in mice that received four different treatments (PBS, GO203, Mdivi-1 and G0203 + Mdivi-1). G + M: GO203 + Mdivi-1. Differences between linked groups were evaluated by a two-tailed Student’s *t* test. *P < 0.05, **P < 0.01; ***P < 0.001; NS not significant.

We further applied inhibitors targeting either MUC1 or mitophagy to test the antitumor activity. CCCP-induced mitophagy in MUC1-expressing cells was mitigated by the MUC1 inhibitor peptide GO203 compared with the control peptide CP2 [32] in all four breast cancer-derived cell lines (Fig. 6A–D and S6A–S6D). Both GO203 and Mdivi-1 significantly inhibited cell growth and mammosphere formation (Figs. 6E–H, S6E, S6F). The combination of GO203 and Mdivi-1 exhibited more potent inhibitory activity in both MDA-MB-468 (Figs. 6E, F, S6E) and BT549 cells (Figs. 6G, H, S6F). To demonstrate whether MUC1 promoted cancer cell progression via Pink1, we performed a cell viability assay and mammosphere formation assay after silencing Pink1 in both MDA-MB-468 and BT549 cell lines (Fig. S6G, S6H). The results showed that Pink1 knockdown intensely constrained CCCP-induced cell proliferation and mammospheres formation not only in MDA-MB-468 cells (Figs. 6I, J, S6I), but also in BT549 cells (Fig. S6J, S6K), indicating MUC1 facilitates breast cancer progression by promoting Pink1-dependent mitophagy. These results were further verified by in vivo data showing that xenograft tumor growth was apparently delayed by treatment with combined GO203 and Mdivi-1 compared to individual inhibitors (Fig. 6K–M). Notably, minimal effect on mouse body weight was noted (Fig. S6L), indicating that the treatment was not toxic to the animals. These results suggest an important role of MUC1-mediated mitophagy in maintaining the tumorigenic properties of cancer cells, which could be exploited as a novel therapeutic target for breast cancers.

### Inverse correlation between ATAD3A and MUC1 in tissues from breast cancer patients

**Fig. 7:**
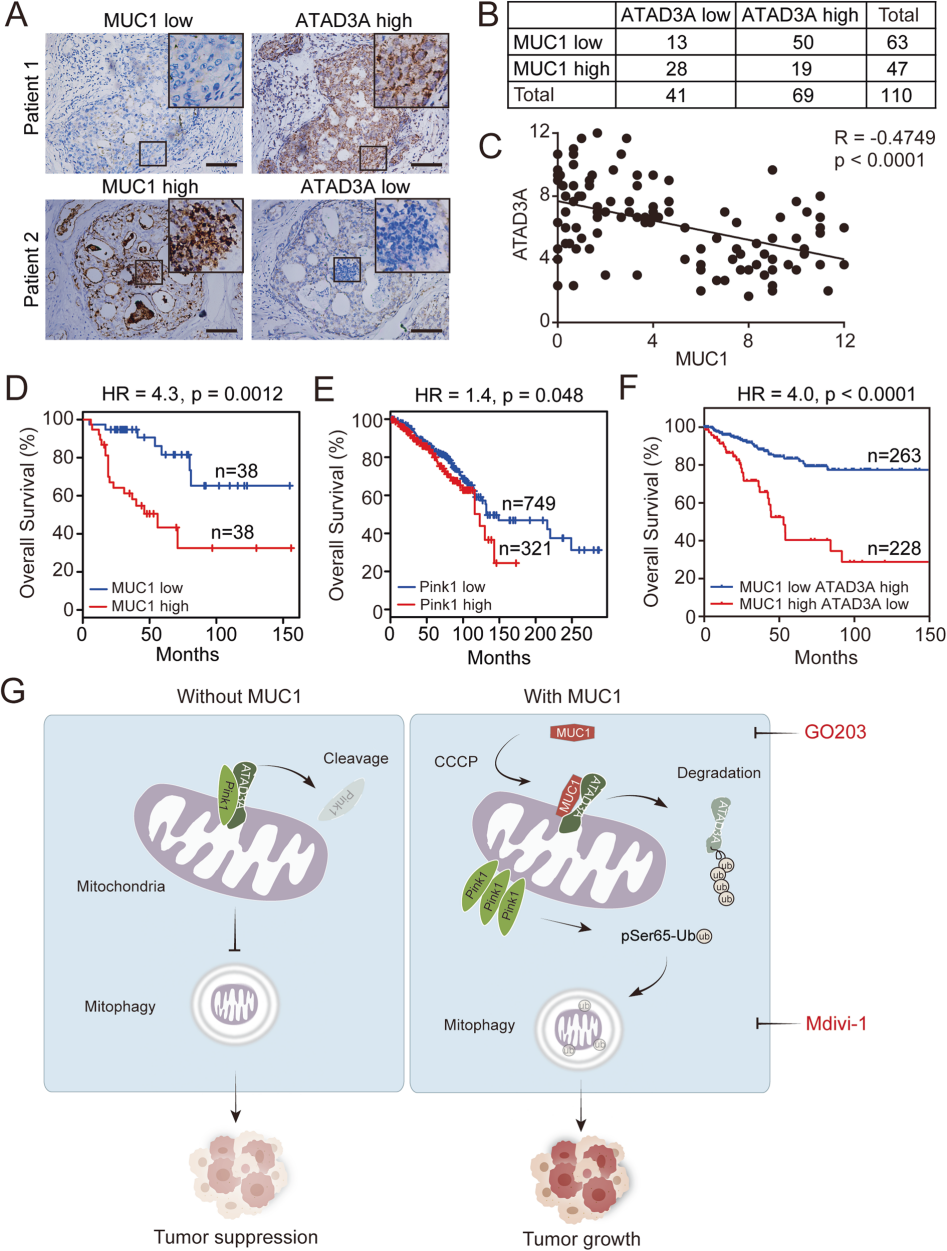
Negative relationship between MUC1 and ATAD3A in breast cancer patients. **A** Representative images of IHC analysis for MUC1 and ATAD3A in 110 breast cancer specimens. Bars: 100 μm. **B** A total of 110 patient samples were divided into four groups, as indicated in the table. **C** The correlation between MUC1 and ATAD3A expression in breast cancer specimens was plotted by Pearson correlation analysis. (**D**) Overall survival study of patients with high and low MUC1 expression was analyzed in breast cancer patients from TCGA datasets (*n* = 76). **E** Overall survival study of patients with high and low Pink1 expression was analyzed in breast cancer patients from TCGA datasets (*n* = 1070). **F** Overall survival study of MUC1 low-ATAD3A high and MUC1 high-ATAD3A low breast cancer patients was analyzed from TCGA datasets (*n* = 491). **G** Schematic overview of the work model. CCCP-induced mitochondrial impairment stimulates the translocation of MUC1 to mitochondria. In mitochondria, MUC1 binds to ATAD3A and promotes ATAD3A degradation through the ubiquitin-proteasome pathway, thereby protecting Pink1 from ATAD3A-mediated cleavage. Accumulation of Pink1 leads to increased pSer65-Ub and subsequently induces mitophagy to promote malignant properties of breast cancer. Targeting both MUC1 and mitophagy based on the combination of GO203 and Mdivi-1 essentially suppresses tumor growth.

To examine the clinical relevance of MUC1 and ATAD3A in breast cancers, we collected 110 samples from breast cancer patients. Immunohistochemical (IHC) staining analysis (Fig. 7A) revealed a negative correlation between MUC1 and ATAD3A not only in all breast cancer patients (Figs. 7B, C) but also in four subtypes of breast cancer patients—luminal A, luminal B, HER2 and TNBC (Fig. S7). These results are consistent with the notion that MUC1 destabilizes ATAD3A and promotes tumor growth in breast cancers. We further analyzed the correlation of MUC1 or Pink1 and the overall survival of breast cancer patients using The Cancer Genome Atlas (TCGA) databases and found that patients with high MUC1 expression (*n* = 38) were considerably associated with shorter overall survival compared to patients with low MUC1 expression (*n* = 38) (Fig. 7D). Patients with high Pink1 expression (*n* = 321) were also significantly associated with shorter overall survival compared to patients with low Pink1 expression (*n* = 749) (Fig. 7E). Notably, the data showed that MUC1 high-ATAD3A low patients (*n* = 228) were considerably associated with shorter overall survival compared to MUC1 low-ATAD3A high patients (*n* = 263) (Fig. 7F).

In conclusion, our work shows that CCCP-induced mitochondrial impairment stimulates the translocation of MUC1 to mitochondria. In mitochondria, MUC1 interacts with ATAD3A and promotes ATAD3A degradation, thereby protecting Pink1 from ATAD3A-mediated cleavage. As a result, the accumulation of full-length Pink1 on the mitochondrial outer membrane triggers mitophagy by inducing phosphorylation of Ub-Ser65, which augments breast cancer oncogenicity. We also show that combined inhibition of MUC1 and mitophagy could represent a novel therapeutic strategy for MUC1-positive breast cancers (Fig. 7G).

## Discussion

Previous studies have reported that MUC1 is localized to the mitochondrial outer membrane to attenuate the apoptotic response to DNA damage [33]. However, the role of MUC1 in mitochondrial quality control remains unknown. Here, for the first time, we reveal that MUC1 augments mitophagy and triggers cancer development.

The most commonly used methods in studying mitophagy in mammalian cells include western blotting, real-time quantitative PCR (RT-qPCR), TEM, the Mitophagy Detection Kit, and fluorescence assays [34]. Using these methods, we found that MUC1 dramatically decreased mitochondrial proteins and mtDNA levels with CCCP treatment, which is a reversible mitochondrial uncoupling factor of mitophagy. The length and number of mitochondria were significantly reduced in MUC1-expressing cells treated with CCCP. Consistently, MUC1 meaningfully improved CCCP-induced colocalization of Mtphagy dye (red) and lysosome dye (green) as well as the colocalization of mitochondrial protein (Tom20) and autophagy-associated protein (LC3B). Altogether, these results illustrate that MUC1 promotes mitophagy in cancer cells. We also observed drug-induced mitophagy in a dose-dependent manner, and a higher concentration of CCCP and O/A induced a higher level of mitophagy, which could be more easily detected by western blotting (Figs. S1G and S1H). Given that Ser65 phospho-ubiquitin is structurally unique and differentially interacts with ubiquitin-binding proteins, pSer65-Ub has been well established as a key event in triggering mitophagy [35]. In support of this notion, MUC1 dramatically augmented pSer65-Ub upon CCCP treatment.

Using LC-MS/MS analysis, we identified the mitochondrial protein ATAD3A that interacted with MUC1 in breast cancer cells. CCCP treatment induced the distribution of MUC1 to mitochondria and promoted the interaction between MUC1 and ATAD3A, indicating a role of MUC1 in responding to mitochondrial damage. After CCCP treatment for only 2 h, higher levels of MUC1 expression did not lead to ATAD3A degradation. MUC1 caused ATAD3A turnover through the ubiquitin‒proteasome pathway. Reliably, an inverse correlation between MUC1 and ATAD3A was found in tumor tissues of breast cancer patients. The CQC motif is essential for oligomerization of MUC1. This motif not only plays a potential role in targeting MUC1-C to mitochondria but also acts as a sensor of redox imbalance [36]. Consistent with these reports, we found that the CQC to AQA mutation markedly disrupted the interaction of MUC1 with ATAD3A and abolished the mitochondrial distribution of MUC1 upon CCCP treatment. Additionally, MUC1-AQA mutation eradicated MUC1-mediated mitophagy. These results validate that the interaction between MUC1 and ATAD3A is crucial for MUC1 translocation to mitochondria and induction of mitophagy. Whether MUC1-induced mitophagy is related to dimerization needs to be addressed in the future.

With the assistance of ATAD3A, Pink1 is imported into the mitochondrial inner membrane, where it is rapidly cleaved by the mitochondrial peptidases PARL and MPP-β [37]. Upon loss of mitochondrial membrane potential, Pink1 is stabilized on the outer mitochondrial membrane, where Pink1 phosphorylates ubiquitin at Ser65 to activate Parkin and Optineurin/NDP52 to mitochondria to trigger mitophagy [24]. Our data demonstrated that MUC1 prevented Pink1 from being cleaved by ATAD3A and consequently promoted Pink1-mediated pSer65-Ub and mitophagy. In addition to the Parkin-dependent pathway, Pink1 can directly activate mitophagy by recruiting calcium binding, coiled-coil domain 2 (NDP52/CALCOCO2), and Optineurin [38]. Several E3 ligases trigger mitophagy in cancer cells in a Pink1-dependent but Parkin-independent manner, such as Ariadne RBR E3 Ub protein ligase 1 (ARIH1) [39] and seven in absentia homolog 1 (SIAH-1) [40]. In support with this point, Parkin is downregulated in multiple cancer cell lines and primary tumors [41, 42]. HeLa cells also lack detectable Parkin expression [43]. Consistent with these reports, we found that MUC1 promoted mitophagy via a Parkin-independent pathway. The factors downstream of the MUC1-Pink1 pathway need to be investigated in the future.

Mitophagy plays controversial roles in tumors, probably depending on tumor type, stage, or metabolic activity [2]. Our results suggested that MUC1/ATAD3A/Pink1 axis-mediated mitophagy improved cancer cell proliferation, mammosphere formation, and tumor growth, suggesting a tumor-promoting role in breast cancers. Several studies have shown that long-term treatment (48 h) with a mitophagy inducer (CCCP or FCCP) induces the depletion of the antioxidant glutathione in mitochondria to increase ROS production with subsequent cell death [44, 45]. However, a short-term treatment with CCCP or FCCP prompts cells to develop protective mechanisms against apoptosis, such as the activation of an antioxidant response [46]. Consistent with this finding, our results showed that short-term (2 h) treatment with CCCP slightly elevated ROS levels and stimulated mitophagy to promote cancer cell proliferation rather than apoptosis in MUC1-positive cells but not in MUC1-negative cells. Similarly, there is no consensus about the role of ATAD3A in cancer. Silencing ATAD3A can promote tumorigenesis in a hepatocellular carcinoma xenograft model, whereas high-ATAD3A levels suggest a better prognosis [47]. Other studies report that silencing ATAD3A reduces the colony formation and invasion ability of tumor cells [48], suggesting that ATAD3A acts as an oncoprotein. Herein, we found that ATAD3A overexpression inhibited cancer cell proliferation, tumor-initiating cell self-renewal potential, and tumorigenesis, supporting a tumor suppressor function of ATAD3A.

Mitophagy often leads to resistance to common chemotherapeutic drugs [49, 50]. The inhibitor of BCL-2/BCL-XL induces mitophagy that causes resistance to cisplatin in ovarian cancer cells [51]. An E3 ubiquitin ligase (ARIH1) that triggers mitophagy protects cells from cisplatin- and etoposide-induced death in lung cancer [39]. The PIWI-like RNA-mediated gene silencing 1 (PIWIL1) protein activates mitophagy and induces doxorubicin resistance in multiple myeloma cells [52]. Consistent with these findings, multiple lines of evidence suggest that inhibition of mitophagy could improve the sensitivity of cancer cells to chemotherapy and targeted therapy [50]. Pink1 downregulation promotes cisplatin-induced apoptosis in non-small cell lung cancer cells [53]. Depletion of FUN14 domain-containing protein 1 (FUNDC1) significantly enhances cell sensitivity to cisplatin and ionizing radiation in cervical cancer [54]. Mdivi-1, a mitophagy inhibitor, has been reported to enhance cisplatin-induced apoptosis in hepatocellular carcinoma but cannot further inhibit tumor volume in vivo [55]. Our data indicate that cosuppression of mitophagy and MUC1 by combining Mdivi-1 and GO203 remarkably eliminated the malignancy characteristics of breast cancer cells in vitro and in vivo. GO203 inhibits MUC1 function by blocking the MUC1-CQC motif, which has been reported to effectively hinder cell proliferation and xenograft tumor growth [56]. Our previous paper [15, 57] and other papers [33, 58] revealed that MUC1 confers resistance to anticancer agents in various cancers and represents be an important target for cancer therapy. Whether MUC1-induced mitophagy contributes to chemoresistance needs to be addressed in the future. However, our data indicate that the combination of mitophagy inhibitors and chemotherapeutic drugs represents an encouraging method to overcome chemoresistance in several MUC1-expressing cancer types.

In summary, the present study reports a novel model in which MUC1/ATAD3A/Pink1 axis-mediated mitophagy plays a crucial role in maintaining the malignant properties of cancer cells, providing a novel therapeutic target for the management of MUC1-positive breast cancers.

## Materials and methods

### Cell culture

T47D human breast cancer cells and HEK293T cells (ATCC) were grown in DMEM (Corning, 10-013-CV) supplemented with 10% fetal bovine serum (FBS) (Gibco, 10099-141C, United States of America) in a 5% CO_2_ incubator at 37 °C. BT549 and SKBR3 human breast cancer cells and HeLa229 human cervical cancer cells were maintained in RPMI1640 (Corning, 10-040-CV, United States of America) supplemented with 10% FBS in a 5% CO_2_ incubator at 37 °C. MDA-MB-468 human breast cancer cells were cultured in L15 medium (BasalMedia, L620KJ, China) supplemented with 10% FBS in a non-CO_2_ incubator at 37 °C. All cell lines were authenticated by short tandem repeat profiling and were free of mycoplasma.

### Drugs and antibodies

The following drugs were used in our experiments: doxorubicin (DOX) (Sigma-Aldrich, D1515, United States of America), dimethylsulfoxide (DMSO) (DingGuo ChangSheng Biotech, DH105-9, China), CCCP (MedChemExpress, HY-100941, United States of America), oligomycin (MedChemExpress, HY-1404-19-9, United States of America), antimycin A1 (MedChemExpress, HY-642-15-9, United States of America), CP2 and GO203 peptides (JRDUN, N/A), cycloheximide (CHX) (Sigma-Aldrich, 66-81-9, United States of America), CQ (Sigma-Aldrich, C6628, United States of America), Mdivi-1 (TargetMol, 338967-87-6, China), NH_4_Cl (Sigma-Aldrich, A0171, United States of America), MG132 (TargetMol, 133407-82-6, China) and BTZ (TargetMol, 179324-69-7, China).

The following antibodies were used in our experiments: anti-MUC1-C (Thermo Scientific, MA5-11202, United States of America) for co-IP and IHC; anti-MUC1-C (Cell Signaling Technology, 16564, United States of America) for western blot and IHC; anti-GFP (Cell Signaling Technologies, 2956, United States of America), anti-Parkin (Cell Signaling Technologies, 4211, United States of America), and anti-LC3 (Proteintech Group, 14600-1-AP, United States of America) for western blot; anti-LC3B (Cell Signaling Technologies, 3868, United States of America) for immunofluorescence assays; anti-Tom20 (Proteintech Group, 11802-1-AP, United States of America) for western blot; anti-Tom20 (BD Transduction Laboratories, 612278, United States of America) for immunofluorescence assays; anti-Tim23 (Proteintech Group, 11123-1-AP, United States of America), anti-COX2 (Proteintech Group, 55070-1-AP, United States of America), anti-COX4 (Proteintech Group, 66110-1-Ig, United States of America), anti-Pink1 (Proteintech Group, 23274-1-AP, United States of America), and anti-ATAD3A (Novus, NBP1-76586, United States of America) for western blot; and anti-ATAD3A (Santa Cruz, sc-376185, United States of America), anti-Ub (Santa Cruz, sc-166553, United States of America), anti-α-tubulin (Sigma-Aldrich, Y-5168, United States of America), anti-V5 (Proteintech Group, 14440-1-AP, United States of America), anti-β-actin antibody (Proteintech Group, 66009-1-Ig, United States of America), and pSer65 ubiquitin (Millipore, ABS1513-I, United States of America) for co-IP.

### Plasmids and transfection

MUC1 deficiency was established using CRISPR/Cas9 systems in MDA-MB-468, BT549, and HeLa229 cells. The MUC1 gRNA sequences included 5′-GCTGCTCCTCACAGTGC-3′ targeting the first exon. After 48 h of viral vector transfection with medium containing 0.8 μg/ml polybrene (Beyotime, C0351, China), cells were selected with 2 μg/ml puromycin (BasalMedia S250J0, China) (HeLa229), 4 μg/ml puromycin (BT549) or 8 μg/ml puromycin (MDA-MB-468). To construct the MUC1-expressing plasmid, full-length MUC1 with an HA tag at the C-terminus was cloned into the pIRESpuro2 vector [14]. To construct GFP-MUC1-CD truncated mutant plasmids, MUC1-CD, MUC1-CD (AQA), CD1-20, CD1-45, CD1-72, and CD46-72 were cloned into the EGFP vector. To construct GST-MUC1-CD-WT and GST-MUC1-CD-AQA mutant plasmids, MUC1-CD and MUC1-CD (AQA) were cloned into the pGEX-4T-1 plasmid [57]. To construct the pSumo3-ATAD3A plasmid, full-length ATAD3A was cloned into the pSumo3 plasmid. Transient transfections were performed according to the manufacturer’s instructions. For MUC1-induced expression, full-length MUC1 with an HA tag at the C-terminus was cloned into the pINDUCER vector. HeLa229/g-MUC1 cells were transfected with the MUC1-pINDUCER vector and selected with 500 μg/ml G418 (BasalMedia, S150J7, China). DOX (1 μg/ml) was used to induce MUC1 expression. shRNA targeting ATAD3A was ordered from the GIPZ Human Rest of Genome shRNA Library. The ATAD3A target sequences are as follows: shATAD3A-1, 5′-CCAGCACCGACTACTACCA-3′; shATAD3A-2, 5′-GAAATGTTTTTGCAGATTT-3′. shRNA targeting Pink1 was ordered from the GIPZ Human Protein Kinase Gene Family Library. The Pink1 target sequences are as follows: shPink1-1, 5′-CCAAGAGAGGTCCCAAGCA-3′; shPink1-2, 5′-CCAAGAGAGGTCCCAAGCA-3′. The control shRNA (shCTL) sequence was 5′-CGCTTACCGATTCAGAATGG-3′. For ATAD3A overexpression, the cells were transfected with the pLX304-Blast-V5/ATAD3A virus vector. Short hairpin RNA or overexpression plasmids and packaging plasmids (pMD2. G and psPAX2) were cotransfected into HEK293T cells. The viral supernatants were collected 48 h after transfection. Transient transfections were performed with Nano293T (NCM, C500T-1, China) or Hilymax DNA Transfection Reagent (Dojindo, H357, Japan) according to the manufacturer’s instructions. pCDNA3.1-3xHA-Parkin (Caemiaolingbio, P5227, China) was purchased from Miaolingbio, Inc.

### Western blot

Cells were collected by trypsinization and resuspended in NETN150 lysis buffer (NaCl 150 mM, NP40 0.5%, Tris 20 mM pH 8.0, EDTA 1 mM) with protease inhibitor cocktail (1:100) (TargetMol, C0001, China). The proteins were quantified by Quick Start Bradford 1x Dye Reagent (Bio-Rad, 5000205, United States of America). The proteins (20 μg) were separated by SDS-PAGE and transferred to a nitrocellulose membrane (Millipore, United States of America). After blocking in 5% nonfat milk for 1 h at room temperature, the membrane was incubated sequentially with primary antibodies overnight at 4 °C and HRP-linked secondary antibodies for 45 min at room temperature. Chemiluminescence detection was performed with ECL (NCM, P10200, China).

### RT-qPCR

As instructed, total RNA was extracted using TRIzol reagent (Invitrogen, 15596018, United States of America). Complementary DNA was synthesized using the M-MLV Reverse Transcriptase synthesis kit (Promega, Madison, M1701, United States of America).

Total DNA was extracted using a TIANamp Genomic DNA Kit according to the manufacturer’s protocol (TIANGEN BIOTECH, DP304-02, China). The amounts of nuclear DNA (ncDNA) and mtDNA were quantified by RT-qPCR for both the beta-2-microglobulin (B2M) gene of ncDNA and the cytochrome-c-oxidase 1 (COX1) gene of mtDNA. RT-qPCR was performed with a Power SYBR Green PCR Master mix kit according to the manufacturer’s instructions (Applied Biosystems, A25742, China). Amplifications were performed in an ABI PRISM 7500 Sequence Detection System (Applied Biosystems, Warrington, UK). Relative transcript quantities were calculated using the ΔΔCt method. Each result was repeated thrice. The primer sequences used are listed in Table S2.

### Immunofluorescence assay

The immunofluorescence assay was performed according to our recent report [15]. The cells were rinsed with PBS, fixed with 4% paraformaldehyde at room temperature for 10 min, and then permeabilized with 0.05% Triton X-100 for 10 min. After 5% GSA blocking at room temperature for 1 h, the cells were incubated sequentially with the specific primary antibodies overnight at 4 °C and fluorescent secondary antibodies in 5% bovine serum albumin for 1 h at 37 °C. Nuclei were stained with DAPI (Vector Laboratories, 28718-90-3, Italy). A confocal microscope (Nikon, Tokyo, Japan) was used to observe all stained slices. Colocalization analysis was performed with Manders’ colocalization coefficients using ImageJ software.

### Analysis of mitophagy

A Mitophagy Detection Kit (Dojindo, MD0, Kumamoto, Japan) was used to detect mitophagy. Then, 100 nmol/l Mtphagy Dye (containing 100 nmol/l MitoBright Deep Red) was added to each group, and the cells were incubated for 30 min. Then, the cells were washed twice with DMEM. After different treatments, the cells were incubated with 1 μmol/l lysosome dye for 30 min. After washing with DMEM twice, the cells were observed by confocal microscopy (Nikon, Tokyo, Japan).

### Co-IP

Protein samples were lysed in NETN150 lysis buffer. The protein lysates were quantified. Proteins (500 μg) in 800 μl lysis buffer were precleared with 30 μl protein A/G plus agarose beads (Santa Cruz, sc-2003, United States of America) at 4 °C for 2 h. The supernatant was incubated with the indicated antibody overnight at 4 °C with rotation. Protein A/G plus agarose beads were added to cell lysates and incubated for 2 h at 4 °C with rotation. The immunoprecipitates were collected by centrifugation and washed with NETN150 buffer thrice. Immunoprecipitated proteins were resolved by SDS-PAGE.

### GST pull-down assay

The plasmids for GST-MUC1-CD-WT, GST-MUC1-CD-AQA, and ATAD3A were transfected into BL21(DE3) cells (Weidi, EC1002). The fusion proteins were prepared. Approximately 60 μg of ATAD3A and 4 μg of GST fusion protein were immobilized in 25 μl of glutathione agarose and equilibrated before being incubated overnight at 4 °C with gentle rocking motion followed by washing thrice with NETN150 buffer (containing 3 mM β-mercaptoethanol). The supernatant was discarded. The bound proteins were resolved by SDS-PAGE. This experiment was implemented in the presence of 3 mM β-mercaptoethanol to eliminate the effect of dimerization.

### Preparation of mitochondrial fractions

A total of 3 × 10^7^ cells were harvested, and the whole-cell lysates were preserved. Then, mitochondrial fractions were prepared using a commercial Cell Mitochondria Isolation Kit (Beyotime, C3601, China). Mitochondrial fractions were dissolved in loading buffer, and proteins were analyzed by immunoblotting.

### Cell viability assay

A total of 3000 cells per well were seeded in 96-well plates, and cell viability was determined using a cell counting kit 8 (CCK8) (NCM, C6005, China) according to the manufacturer’s protocol. CCK8 was added and incubated for another 2 h, and then the absorbance at a wavelength of 450 nm was measured by Multiskan FC (Thermo Scientific, USA) simultaneously every day. Each experiment was repeated thrice with three samples.

### Mammosphere formation assay

A total of 3000 cells per well were plated in ultralow attachment plates (Corning, 3473, United States of America) in DMEM/F12 (BasalMedia, L310KJ, China) serum-free medium containing 0.4% bovine serum albumin (Basal Medium, S476T7, China) supplemented with 20 ng/ml epithelial growth factor (PerpoTech, 500-P45), 20 ng/ml basic fibroblast growth factor (PerpoTech, 500-P18, United States of America), 50 μg/ml insulin (LABLEAD, I5500, China) and 1 × B27 (Gibco, 17504044, United States of America), which was described previously [15]. Cells were incubated for 4–6 days, and mammospheres (>50 μm in diameter) were counted under a microscope (Nikon Eclipse Ti, Tokyo, Japan).

### ROS assay

A Reactive Oxygen Species Assay Kit (Beyotime, S0033S, China) was used to detect ROS levels. The principal component is DCFH-DA, which is easily oxidized to fluorescent dichlorofluorescein (DCF) by intracellular ROS. After different treatments, the cells were washed twice with medium without FBS. Then, 10 μm/l DCFH-DA was added to each group, and the cells were incubated for 20 min. After washing twice with medium without FBS, the cells were measured at 488 nm excitation and 525 nm emission by a fluorescence microscope (Nikon Eclipse Ti, Tokyo, Japan).

### TEM imaging

After DMSO or CCCP treatment for 3 h, HeLa229 g-CTL/g-MUC1 cells were fixed in a mixture of 2% glutaraldehyde in PBS (4 °C, pH 7.4, 0.1 M) for 2 h. The cells were fixed using buffered 1% osmium tetroxide for 2 h. Then, the samples were dehydrated using ethyl alcohol and replaced by acetone. The blocks were sectioned using an ultramicrotome (LKB V, LKB, Sweden). The slides were double-stained with uranyl acetate and lead citrate. Finally, the slides were visualized and photographed using TEM (H-7650, Hitachi, Japan).

### Mass spectrometry

HeLa229 cells were lysed in NETN150 lysis buffer with protease inhibitor cocktails and phosphatase inhibitor cocktails (Sigma-Aldrich, United States of America). The lysates were immunoprecipitated using anti-MUC1-C antibody or IgG agarose overnight at 4 °C with rotation. Then, Protein A/G plus agarose beads were added to the cell lysates and incubated for 2 h at 4 °C with rotation. The beads were washed five times. Immunoprecipitated proteins were separated by SDS-PAGE and stained with Coomassie blue. The band excised from the gel was subjected to reduction, carbamidomethylation and tryptic digestion. Peptide sequences were determined by mass spectrometry using an Orbitrap Fusion LUMOS mass spectrometer (Thermo Scientific, United States of America) connected to an Easy-nLC 1200 via an Easy Spray (Thermo Scientific, United States of America).

### Xenograft experiments

Animal protocols were performed in accordance with the Shanghai Medical Experimental Animal Care Guidelines. The research was approved by the Institutional Animal Care and Use Committee of Shanghai Jiao Tong University School of Medicine. Tumor cells were injected subcutaneously into the ventral flanks of 6-week-old female BALB/c nude mice. Mice bearing tumors (4 mm × 4 mm) were randomly and blindly divided into four groups of six mice each and treated intraperitoneally (i) with vehicle control each day for two weeks or (ii) with 15 mg/kg GO203 each day for two weeks or (iii) with 50 mg/kg Mdivi-1 every three days for two weeks or (iv) with GO203 and Mdivi-1 combination as in (ii) and (iii). Mdivi-1 was formulated in 6% DMSO/30% PEG300/3% Tween-80. Mice injected with 5 × 10^6^ MDA-MB-468 g-CTL, g-MUC1, g-CTL/ATAD3A, and g-MUC1/ATAD3A cells were blindly assigned to four groups. Tumor growth was monitored by caliper rule every two days. The volume was calculated according to the formula: *V* = 0.52 × length × width^2^. At the end of treatment, the animals were euthanized, and the tumors were isolated and weighed. The animals without tumors were weighed.

### Human breast cancer specimens and IHC

A total of 110 patients who underwent breast cancer surgery from October 2010 to May 2015 at Xiangya Hospital of Central South University were randomly selected. Slides were reviewed, and blocks were identified based on the presence of adequate tumors and the representative nature of the overall tumor. Patient age ranged from 22-74 years old, and all cases were confirmed by pathology after the operation.

After paraffin sections were dewaxed and hydrated, endogenous peroxidase activity was blocked using 3% hydrogen peroxide. The mouse anti-human monoclonal ATAD3A primary antibody (Santa Cruz, sc-376185) was used at a dilution of 1:50, and the rabbit anti-human monoclonal MUC1 primary antibody (Cell Signaling Technology, 16564, United States of America) was used at a dilution of 1:200 overnight at 4 °C. After incubation with a streptavidin enzyme conjugate, the complex was visualized by color development with DAB (ZSGB Biological, China), and the slides were counterstained with hematoxylin.

According to the positive cell proportion and the staining intensity, IHC staining was blindly scored as low or high and rated on a range of 0-12 (0: ≤5%, 1: <25%, 2: <50%, 3: <75%, and 4: ≥75%) and (0: negative, 1: weak, 2: intermediate and 3: strong). This study was compliant with the related ethical regulations regarding research involving human participants.

### Statistical analysis

All statistical analyses were performed by GraphPad Prism 6 (GraphPad Software, La Jolla, CA, United States of America). The results are shown as the means of three independent experiments (*n* = 3). Differences between linked groups were evaluated by a two-tailed Student’s *t* test. *P < 0.05; **P < 0.01; and ***P < 0.001; NS indicates not significant.

## Supplementary information

Supplement

Original western blots

Reproducibility checklist

## Data Availability

All data generated or analyzed during this study are included in this published article and its supplementary information files. Additional data are available from the corresponding author upon reasonable request.
