# Supplementary material for: The oncoprotein MUC1 facilitates breast cancer progression by promoting Pink1-dependent mitophagy via ATAD3A destabilization
Source: Cell Death Dis. 2022 Oct 26;13(10):899. doi: 10.1038/s41419-022-05345-z (PMC9606306; doi:10.1038/s41419-022-05345-z)
Supplement: Supplementary file 1 — Supplement [file 41419_2022_5345_MOESM1_ESM.docx]

**Supplementary figures**

**
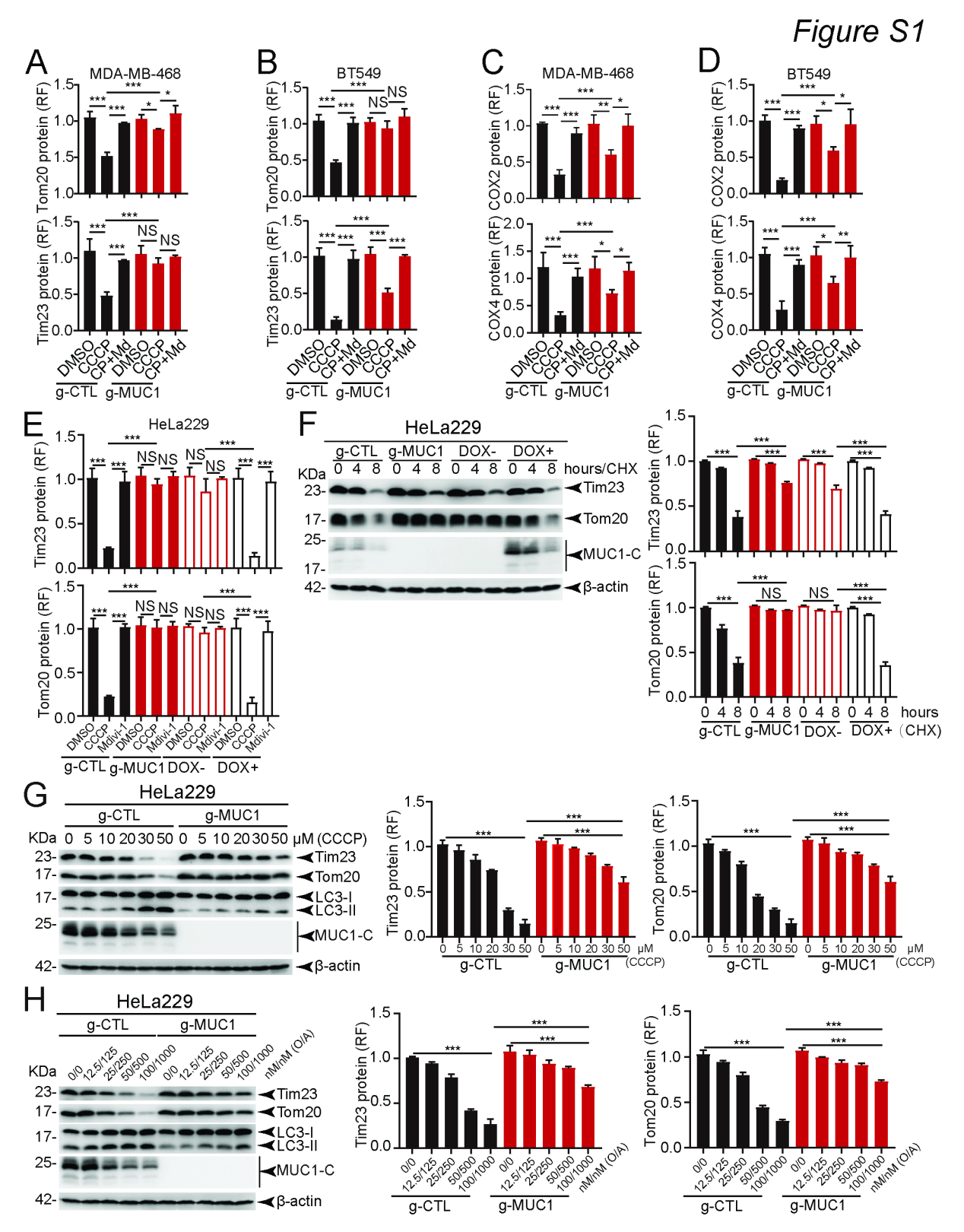
**

**
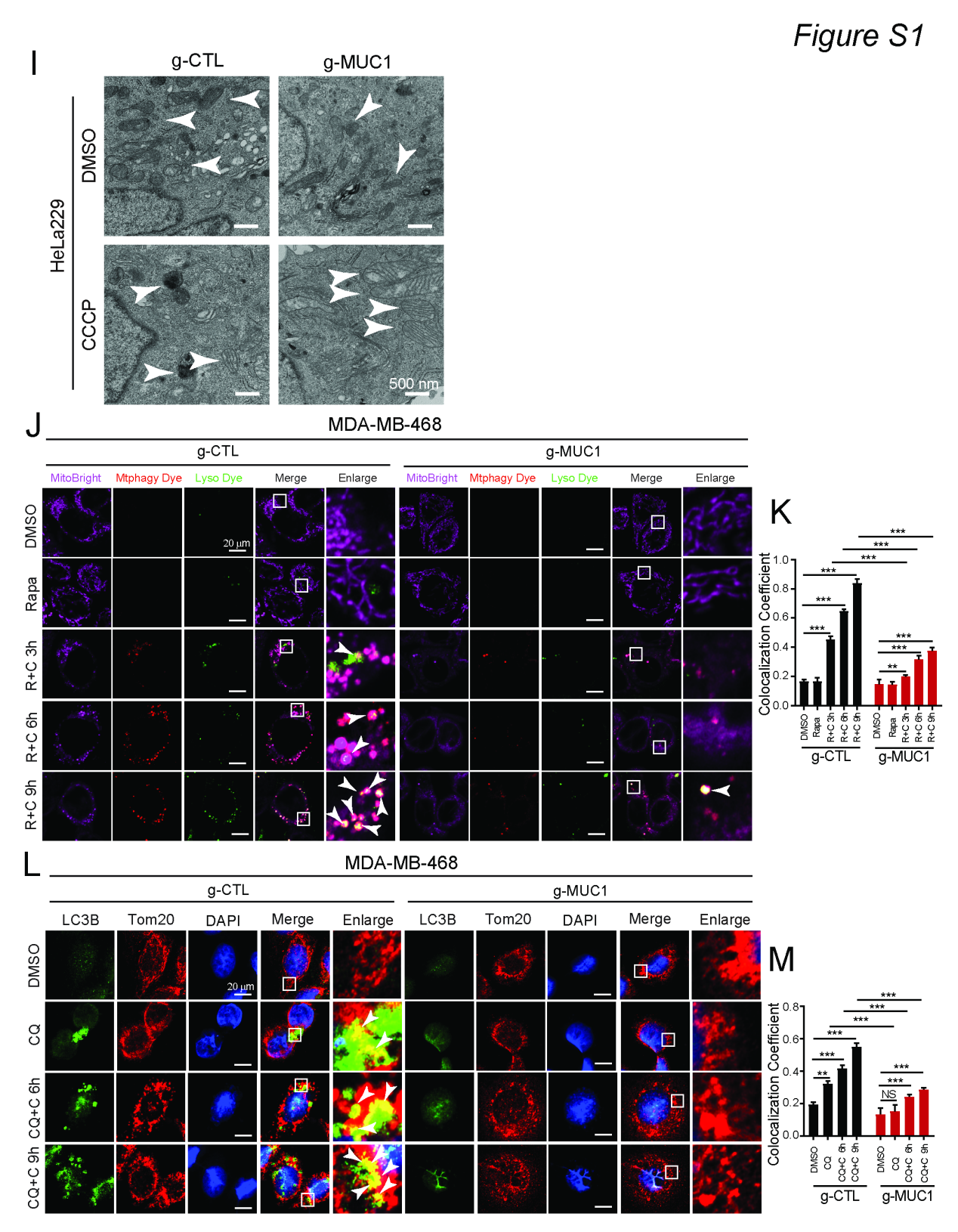
**

**Fig. S1 MUC1 enhances CCCP-induced mitophagy.**

(S1A, S1C) Western blotting was performed to detect proteins as indicated in MDA-MB-468/g-CTL and MDA-MB-468/g-MUC1 cells treated with DMSO, 5 μM CCCP (24 h) or 5 μM CCCP (24 h) + 10 μM Mdivi-1 (24 h). Quantification of mitochondrial proteins was calculated in (Figs. 1A and 1C).

(S1B, S1D) Western blotting was performed to detect proteins as indicated in BT549/g-CTL and BT549/g-MUC1 cells treated with DMSO, 10 μM CCCP (24 h) or 10 μM CCCP (24 h) + 10 μM Mdivi-1 (24 h). Quantification of mitochondrial proteins was calculated in (Figs. 1B and 1D).

(S1E) After treatment with DMSO, 30 μM CCCP (24 h) or 30μM Mdivi-1 (24 h), western blotting was performed to detect proteins with the indicated antibodies in HeLa229/g-CTL, HeLa229/g-MUC1, and HeLa229/g-MUC1 treated with 1 μg/ml DOX for 48 h to induce MUC1 expression (DOX+) and without DOX treatment (DOX-) as a negative control. Quantification of mitochondrial proteins was calculated in (Fig. 1E).

(S1F) After treatment with 10 μg/ml CHX (0 h, 4 h, 8 h), western blotting was performed to detect proteins with the indicated antibodies in four cell lines--HeLa229/g-CTL, HeLa229/g-MUC1, HeLa229/g-MUC1 treated with 1 μg/ml DOX for 48 h to induce MUC1 expression (DOX+) and without DOX treatment (DOX-) as a negative control. Quantification of mitochondrial proteins was calculated.

(S1G) HeLa229/g-CTL and HeLa229/g-MUC1 cells were treated with different concentration of CCCP (0, 5, 10, 20, 30, 50 (μM)) for 24 h. Western blotting was performed to detect proteins as indicated. Quantification of mitochondrial proteins was calculated.

(S1H) HeLa229/g-CTL and HeLa229/g-MUC1 cells were treated with different concentration of O/A (0/0, 12.5/125, 25/250, 50/500, 100/1000 (nM/nM)) for 24 h. Western blotting was performed to detect proteins as indicated. Quantification of mitochondrial proteins was calculated. O/A, Oligomycin and Antimycin A1.

(S1I) Representative TEM micrographs of HeLa229/g-CTL and HeLa229/g-MUC1 cells treated with DMSO or CCCP (30 μM) for 3 h. Arrows indicated mitochondria. Bars: 500 nm.

(S1J, S1K) Representative fluorescence images with Mtphagy Dye (Red), Lysosome Dye (Green) and MitoBright (Purple) staining in MDA-MB-468 cells stimulated with DMSO, 5 μM CCCP (3 h, 6 h, 9 h) and 500 nM Rapamycin (12 h) for indicated time (S1J). Arrow indicated colocalization pots. Frequency of co-localized Mtphagy Dye (Red) and Lysosome Dye (Green) in MDA-MB-468/g-CTL and MDA-MB-468/g-MUC1 cells was calculated (S1K). Rapa: Rapamycin. R+C: Rapamycin + CCCP. Bars: 20 μm.

(S1L, S1M) MDA-MB-468/g-CTL and MDA-MB-468/g-MUC1 cells were treated with DMSO, 5 μM CCCP (6 h, 9 h) and 100 μM CQ (12 h). Immunofluorescence of the co-localization of Tom20 (Red) and LC3B (Green) was analyzed by confocal microscope (S1L). Arrow indicated colocalization pots. Frequency of co-localized Tom20 and LC3B in MDA-MB-468/g-CTL and MDA-MB-468/g-MUC1 cells was calculated (S1M). Nuclei were stained with DAPI (Blue). CQ+C: CQ + CCCP. Bars: 20 μm. The data represent the mean ± SD from three independent experiments. Differences between linked groups were evaluated by a two-tailed Student's t test. * p < 0.05, ** P < 0.01; *** P < 0.001; NS, not significant.

**
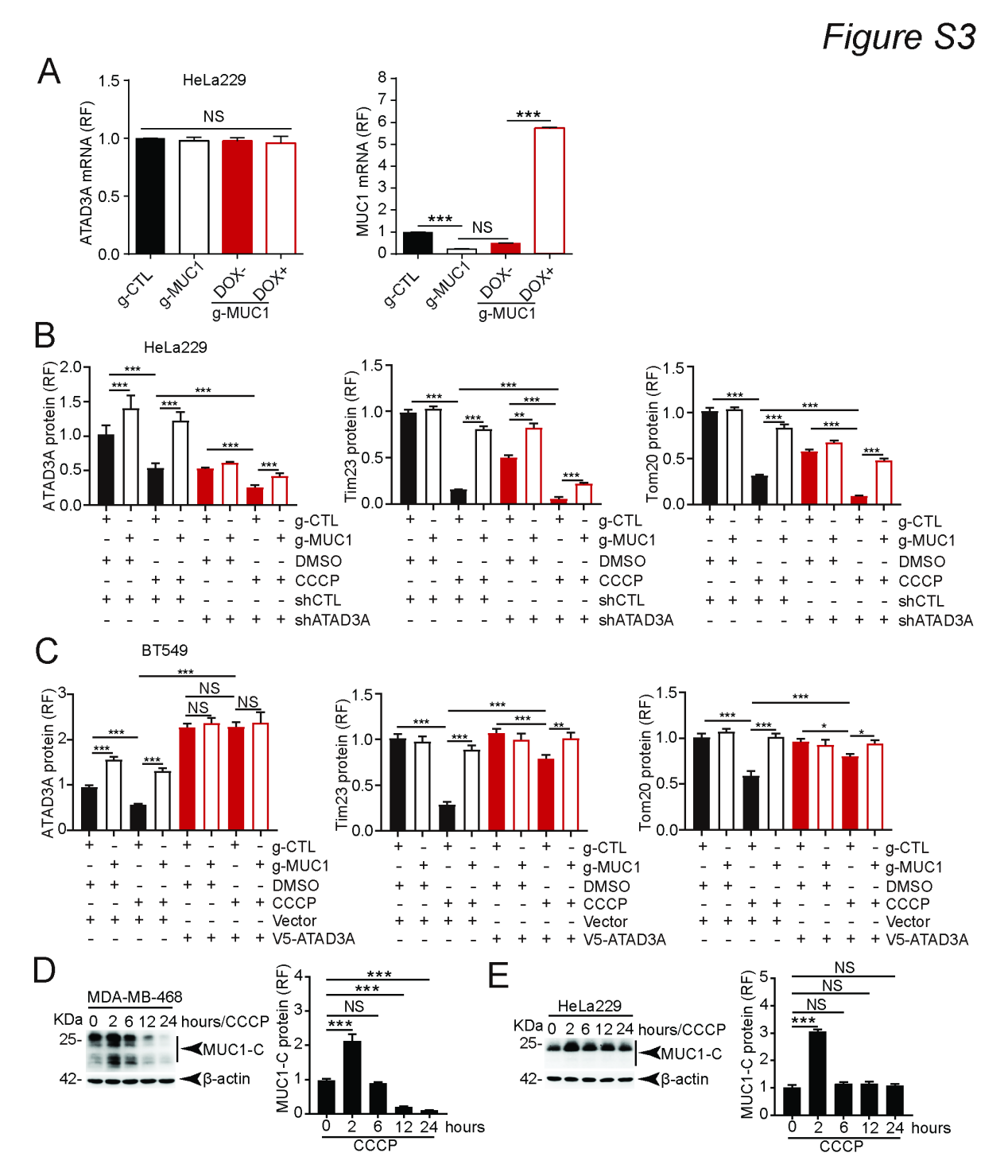
**

**Fig. S3 MUC1 downregulates ATAD3A, and ATAD3A inhibits MUC1-mediated mitophagy.**

(S3A) RT-qPCR was performed to detect ATAD3A mRNA levels (left) and MUC1 mRNA levels (right) in four cell lines--HeLa229/g-CTL, HeLa229/g-MUC1, and HeLa229/g-MUC1 cells treated with 1 μg/ml DOX for 48 h to induce MUC1 expression (DOX+) and without DOX treatment (DOX-) as a negative control.

(S3B) HeLa229/g-CTL and HeLa229/g-MUC1 cells were transfected with shCTL and shATAD3A lentiviral vectors then treated with DMSO or 30 μM CCCP (24 h). Western blotting was performed using antibodies as indicated. Quantification of mitochondrial proteins was calculated in (Fig. 3I).

(S3C) BT549/g-CTL and BT549/g-MUC1 cells were transfected with Vector and ATAD3A-V5 lentiviral vectors, then treated with DMSO or 10 μM CCCP (24 h). Western blotting was performed using the indicated antibodies. Quantification of mitochondrial proteins was calculated in (Fig. 3J).

(S3D) HeLa229 cells were treated with 30 μM CCCP for 2 h, 6 h, 12 h and 24 h. Then western blotting was performed to detect MUC1-C expression. Quantification of MUC1-C proteins was calculated.

(S3E) MDA-MB-468 cells were treated with 5 μM CCCP for 2 h, 6 h, 12 h and 24 h. Then western blotting was performed to detect MUC1-C expression. Quantification of MUC1-C proteins was calculated. The data represent the mean ± SD from three independent experiments. Differences between linked groups were evaluated by a two-tailed Student's t test. * p < 0.05, ** P < 0.01; *** P < 0.001; NS, not significant.

**
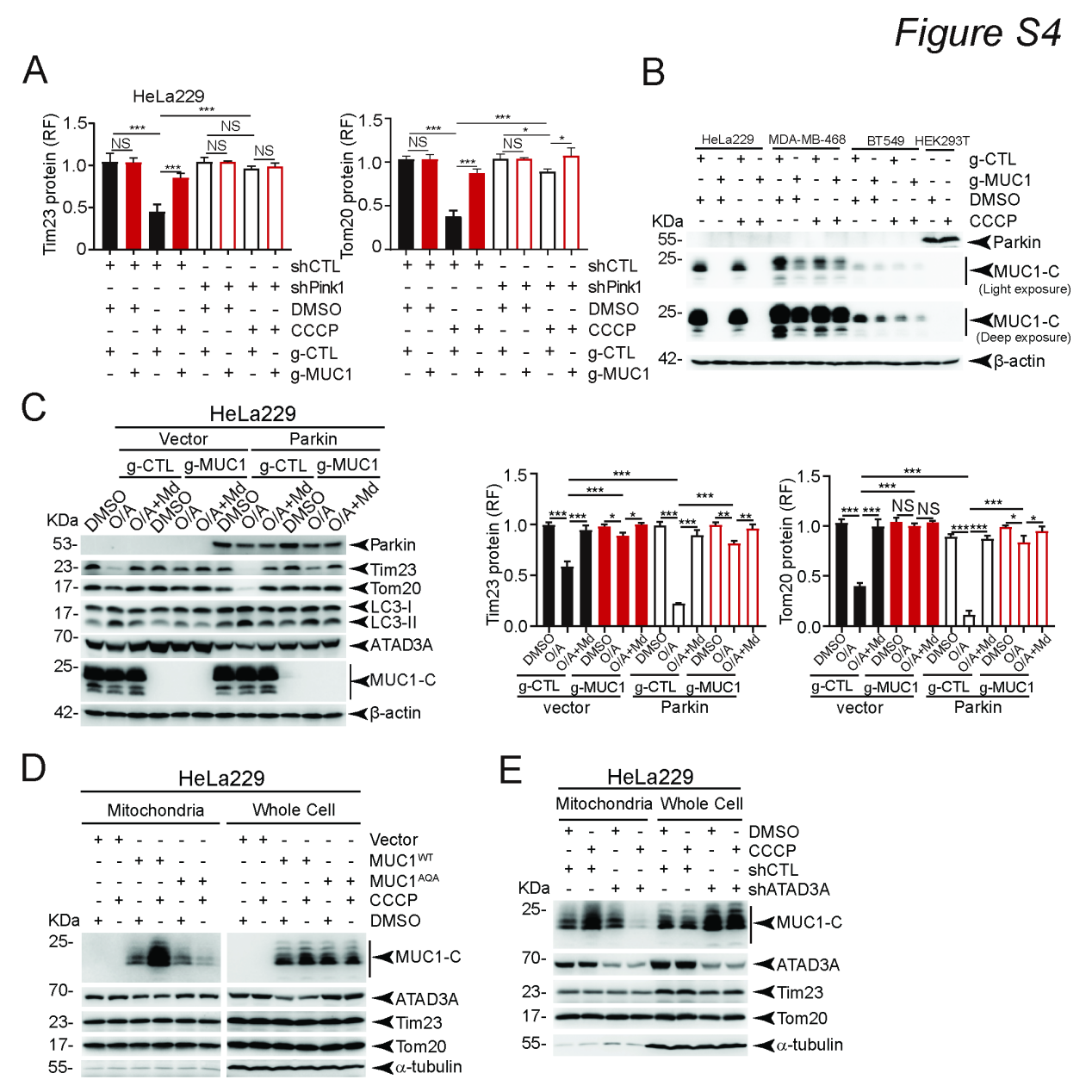
**

**Fig. S4 MUC1 translocates to mitochondria to protect Pink1 from ATAD3A-mediated cleavage.**

(S4A) HeLa229/g-CTL and HeLa229/g-MUC1 cells were transfected with shCTL and shPink1 lentiviral vectors, then treated with DMSO or 30 μM CCCP (24 h). Western blotting was performed using antibodies as indicated. The relative quantification of protein levels was analyzed in (Fig. 4E).

(S4B) HeLa229/g-CTL, HeLa229/g-MUC1, MDA-MB-468/g-CTL, MDA-MB-468/g-MUC1, BT549/g-CTL, BT549/g-MUC1 and HEK293T cells were treated with CCCP for 24h. Western blotting was performed to detect Parkin protein levels. HEK293T acted as a positive control of Parkin expression.

(S4C) HeLa229/g-CTL and HeLa229/g-MUC1 cells were transfected with Vector and Parkin-HA plasmids for 48 h and then treated with DMSO, 25 nM / 250 nM O/A (24 h) or a combination of 25 nM / 250 nM O/A (24 h) and 30 μM Mdivi-1 (24 h). Western blotting was performed as indicated. Quantification of mitochondrial proteins was calculated and the relative statistical analysis. O/A, Oligomycin and Antimycin A1. O/A + Md: O/A + Mdivi-1.

(S4D) HeLa229/g-MUC1 cells were transfected with Vector, MUC1-CD-WT and MUC1-CD-AQA plasmids and then treated with DMSO and 30 μM CCCP (2 h). Sediment from mitochondrial fraction were detected by western blotting. Tim23, Tom20 and ATAD3A were used as mitochondrial markers and α-tubulin was used as a cytosolic marker.

(S4E) HeLa229 cells were transfected with shCTL and shATAD3A lentiviral vectors and then treated with DMSO and 30 μM CCCP (2 h). Sediment from mitochondrial fraction was detected by western blotting. Tim23, Tom20 and ATAD3A were used as mitochondrial markers, and α-tubulin was used as a cytosolic marker. The data represent the mean ± SD from three independent experiments. Differences between linked groups were evaluated by a two-tailed Student's t test. * p < 0.05, ** P < 0.01; *** P < 0.001; NS, not significant.


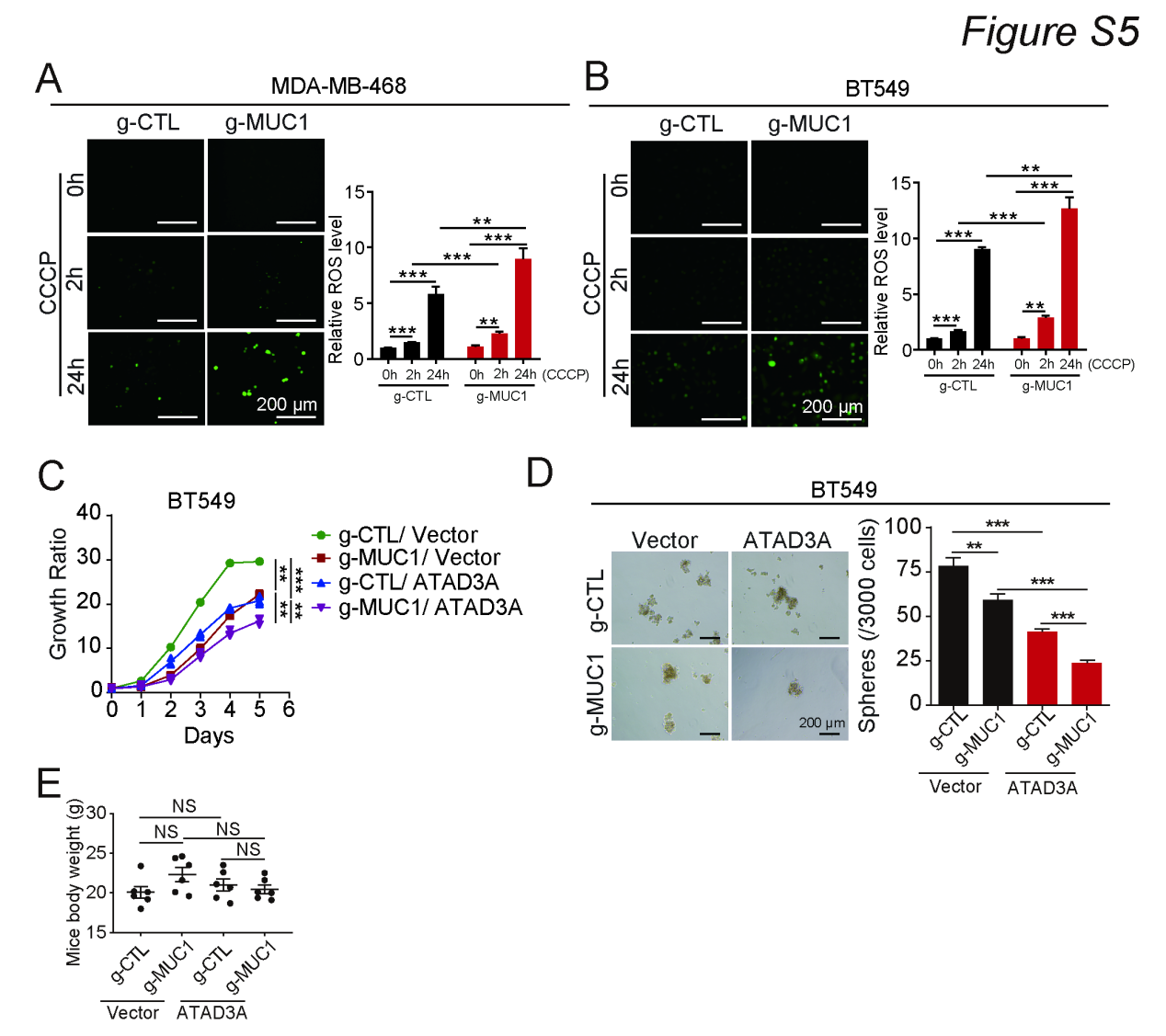


**Fig. S5 MUC1-mediated mitophagy promotes tumor cell malignancy.**

(S5A) Representative fluorescence images with DCFH-DA (Green) staining using ROS Assay Kit in MDA-MB-468/g-CTL and MDA-MB-468/g-MUC1 cells stimulated with DMSO, 5 μM CCCP (2 h), and 5 μM CCCP (24 h). The relative ROS levels were calculated. Bars: 200 μm.

(S5B) Representative fluorescence images with DCFH-DA (Green) staining using ROS Assay Kit in BT549/g-CTL and BT549/g-MUC1 cells stimulated with DMSO, 10 μM CCCP (2 h), and 10 μM CCCP (24 h). The relative ROS levels were calculated. Bars: 200 μm.

(S5C) BT549/g-CTL and BT549/g-MUC1 cells were transfected with Vector and ATAD3A-V5 lentiviral vectors, then cells were subjected to viability assay.

(S5D) BT549/g-CTL and BT549/g-MUC1 cells were transfected with Vector and ATAD3A-V5 lentiviral vectors, then cells were subjected to the mammospheres formation assay. Representative mammospheres > 50 μm in diameter are shown. The number of mammospheres was calculated. Bars: 200 μm. The data represent the mean ± SD from three independent experiments.

(S5E) The weights of mice injected with the indicated MDA-MB-468 cells (g-CTL/g-CTL-ATAD3A, g-MUC1/g-MUC1-ATAD3A) were compared. Differences between linked groups were evaluated by a two-tailed Student's t test. * P < 0.05, ** P < 0.01; *** P < 0.001; NS, not significant.

**
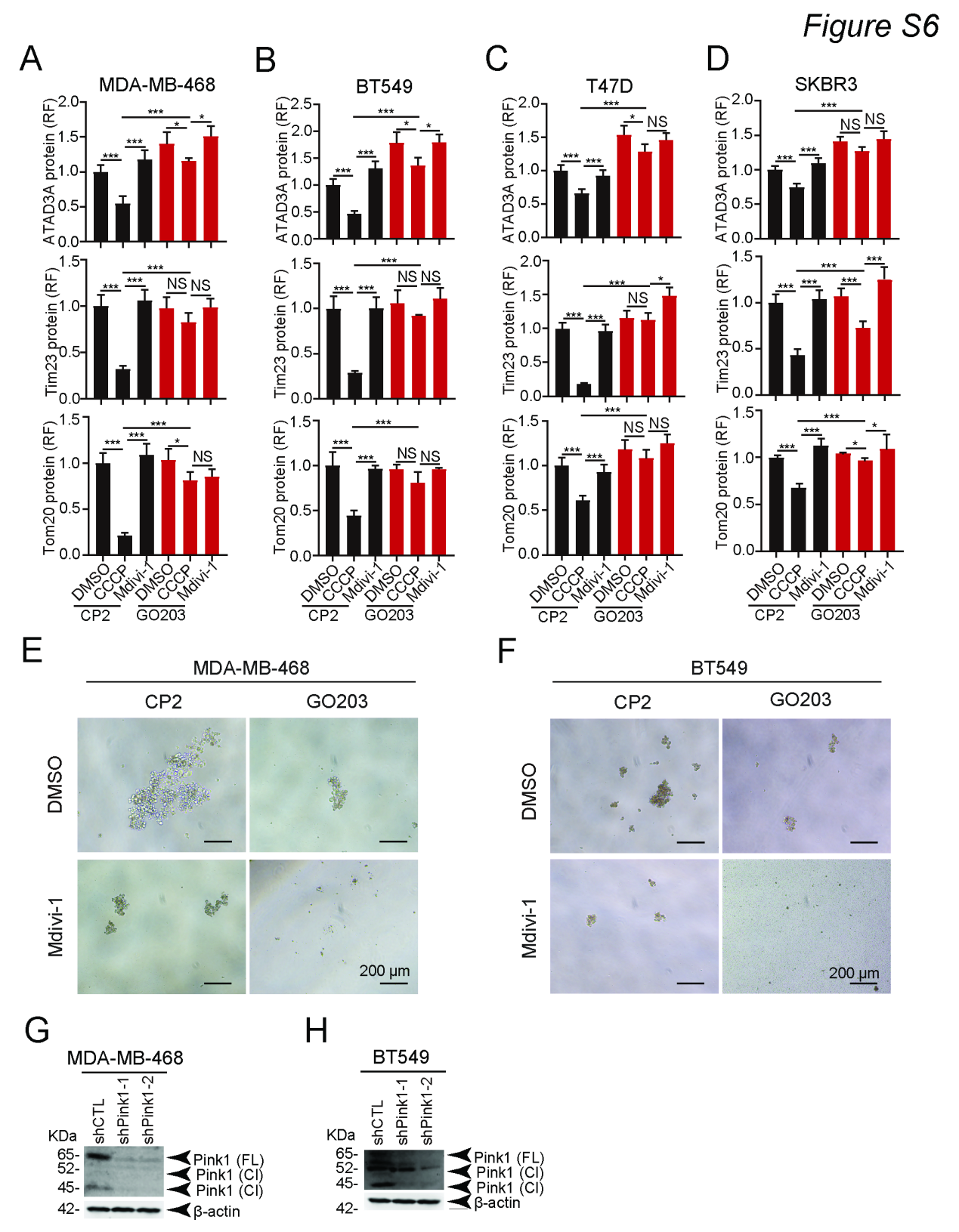
**

**
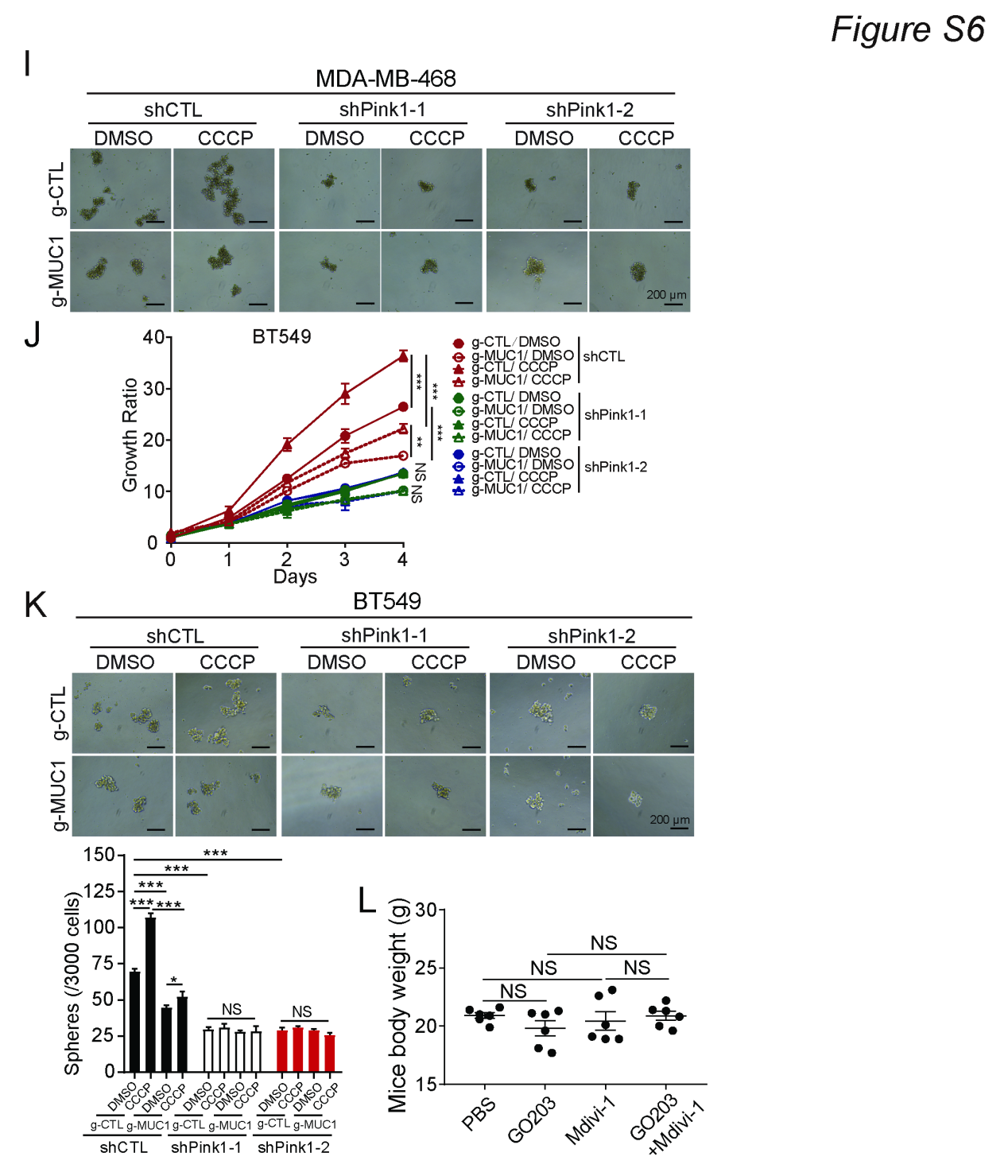
**

**Fig. S6 Targeting MUC1-mitophagy signaling inhibits tumor malignancy.**

(S6A) MDA-MB-468 cells were treated with 5 μM CP2 (48 h) and 5 μM GO203 (48 h) then treated with DMSO, 5 μM CCCP (24 h) or 10 μM Mdivi-1 (24 h). Western blotting was performed to detect proteins as indicated. The relative quantification of protein levels was analyzed in (Fig. 6A).

(S6B) BT549 cells were treated with 5 μM CP2 (48 h) and 5 μM GO203 (48 h) and then treated with DMSO, 10 μM CCCP (24 h) or 10 μM Mdivi-1 (24 h). Western blotting was performed to detect proteins as indicated. The relative quantification of protein levels was analyzed in (Fig. 6B).

(S6C) T47D cells were treated with 10 μM CP2 (48 h) and 10 μM GO203 (48 h) then treated with DMSO, 10 μM CCCP (24 h) or 10 μM Mdivi-1 (24 h). Western blotting was performed to detect proteins as indicated. The relative quantification of protein levels was analyzed in (Fig. 6C).

(S6D) SKBR3 cells were treated with 10 μM CP2 (48 h) and 10 μM GO203 (48 h) then treated with DMSO, 10 μM CCCP (24 h) or 10 μM Mdivi-1 (24 h). Western blotting was performed to detect proteins as indicated. The relative quantification of protein levels was analyzed in (Fig. 6D).

(S6E) The mammospheres formation assay was performed in MDA-MB-468 cells treated with 5 μM GO203 and 10 μM Mdivi-1 alone or in combination. Representative mammospheres > 50 μm in diameter are shown. Bars: 200 μm.

(S6F) The mammospheres formation assay was performed in BT549 cells treated with 5 μM GO203 and 10 μM Mdivi-1 alone or in combination. Representative mammospheres > 50 μm in diameter are shown. Bars: 200 μm.

(S6G) MDA-MB-468 cells were transfected with shCTL, shPink1-1 and shPink1-2 lentiviral vector for 48 h. Western blotting was performed to detect Pink1 protein levels as indicated.

(S6H) BT549 cells were transfected with shCTL, shPink1-1 and shPink1-2 lentiviral vector for 48 h. Western blotting was performed to detect Pink1 protein levels as indicated.

(S6I) MDA-MB-468/g-CTL and MDA-MB-468/g-MUC1 cells were transfected with shCTL, shPink1-1 and shPink1-2 lentiviral vector for 48 h, then treated with DMSO and 5 μM CCCP (2 h). Cells were subjected to the mammospheres formation assay. Representative images are shown. Bars: 200 μm. The number of mammospheres > 50 μm in diameter was calculated.

(S6J) BT549/g-CTL and BT549/g-MUC1 cells were transfected with shCTL, shPink1-1, and shPink1-2 lentiviral vector for 48 h and then treated with DMSO and 10 μM CCCP (2 h). The cell viability assay was performed.

(S6K) BT549/g-CTL and BT549/g-MUC1 cells were transfected with shCTL, shPink1-1, and shPink1-2 lentiviral vector for 48 h and then treated with DMSO and 10 μM CCCP (2 h). Cells were subjected to the mammospheres formation assay. Representative images are shown. Bars: 200 μm. The number of mammospheres > 50 μm in diameter was calculated. The data represent the mean ± SD from three independent experiments.

(S6L) The weights of mice receiving four different treatments (PBS, GO203, Mdivi-1 and G0203 / Mdivi-1) were compared. Differences between linked groups were evaluated by a two-tailed Student's t test. * p < 0.05, ** P < 0.01; *** P < 0.001; NS, not significant.


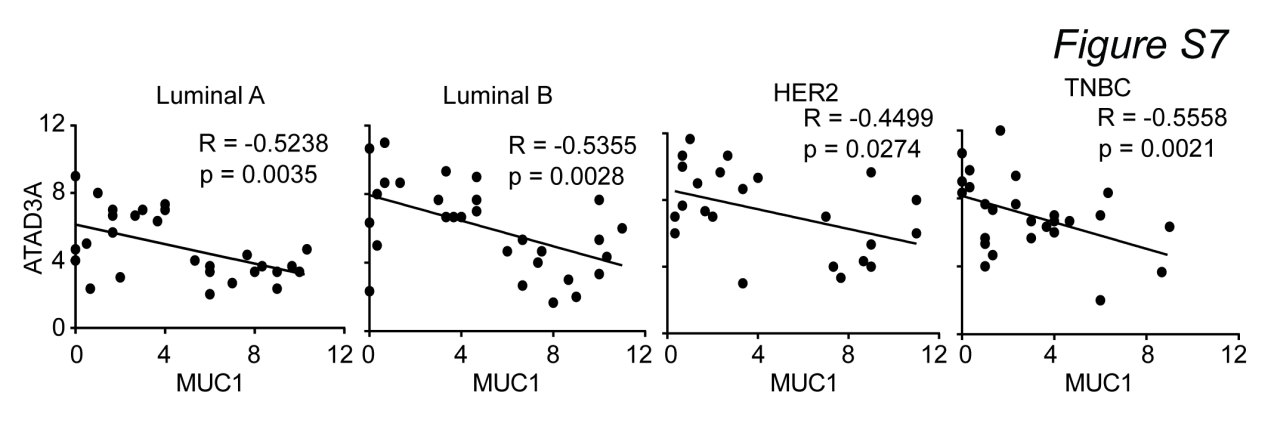


**Fig. S7 Negative relationship of MUC1 and ATAD3A in breast cancer patients.**

The correlation between MUC1 and ATAD3A expression in four subtypes (Luminal A, Luminal B, HER2 and TNBC) of breast cancer specimens respectively was plotted by Pearson correlation analysis.

**Supplementary tables**

| **Table S1. MUC1 interacting proteins** | | | |
| --- | --- | --- | --- |
| Accession Number | Identified Proteins | Molecular Weight (KDa) | Score |
| sp\|Q9NVI7\|ATAD3A_HUMAN | ATPase family AAA domain-containing protein 3A | 71 | 204.41 |
| sp\|D6RHI9\|RNASET2_HUMAN | Ribonuclease T2 | 29 | 106.11 |
| sp\|Q5T9A4\|ATAD3B_HUMAN | ATPase family AAA domain-containing protein 3B | 73 | 105.10 |
| sp\|P07437\|TUBB_HUMAN | Tubulin beta chain | 50 | 26.55 |
| sp\|P67809\|YBX1_HUMAN | Nuclease-sensitive element-binding protein 1 | 36 | 26.50 |
| sp\|P68371\|TUBB4B_HUMAN | Tubulin beta-4B chain | 50 | 21.11 |
| sp\|P06733\|ENO1_HUMAN | Alpha-enolase | 47 | 14.74 |
| sp\|P04259\|KRT6B_HUMAN | Keratin, type II cytoskeletal 6B | 60 | 14.43 |
| sp\|P06576\|ATP5B_HUMAN | ATP synthase subunit beta, mitochondrial | 57 | 11.05 |
| sp\|P04075\|ALDOA_HUMAN | Fructose-bisphosphate aldolase A | 45 | 10.13 |
| sp\|P04899\|GNAI2_HUMAN | Guanine nucleotide-binding protein G(i) subunit alpha-2 | 40 | 10.03 |
| sp\|Q16576\|RBBP7_HUMAN | Histone-binding protein RBBP7 | 52 | 9.36 |
| sp\|Q13509\|TUBB3_HUMAN | Tubulin beta-3 chain | 50 | 9.35 |
| sp\|Q9Y3A4\|RRP7A_HUMAN | Ribosomal RNA-processing protein 7 homolog A | 32 | 9.21 |
| sp\|P04350\|TUBB4A_HUMAN | Tubulin beta-4A chain | 50 | 8.93 |
| sp\|P62701\|RPS4X_HUMAN | 40S ribosomal protein S4, X isoform | 30 | 8.56 |
| sp\|P46783\|RPS10_HUMAN | 40S ribosomal protein S10 | 19 | 7.28 |
| sp\|Q9BUF5\|TUBB6_HUMAN | Tubulin beta-6 chain | 50 | 6.80 |
| sp\|Q09028\|RBBP4_HUMAN | Histone-binding protein RBBP4 | 46 | 6.35 |
| sp\|Q5M775\|SPECC1_HUMAN | Cytospin-B | 119 | 6.35 |
| sp\|Q9Y3F4\|STRAP_HUMAN | Serine-threonine kinase receptor-associated protein | 40 | 6.26 |
| sp\|Q01130\|SRSF2_HUMAN | Serine/arginine-rich splicing factor 2 | 25 | 6.08 |
| sp\|P62266\|RPS23_HUMAN | 40S ribosomal protein S23 | 16 | 6.05 |
| sp\|P07195\|LDHB_HUMAN | L-lactate dehydrogenase B chain | 37 | 5.62 |
| sp\|Q9UDY4\|DNAJB4_HUMAN | DnaJ homolog subfamily B member 4 | 38 | 5.62 |
| sp\|Q15233\|NONO_HUMAN | Non-POU domain-containing octamer-binding protein | 54 | 5.32 |
| sp\|Q03701\|CEBPZ_HUMAN | CCAAT/enhancer-binding protein zeta | 121 | 5.28 |
| sp\|Q99613\|EIF3C_HUMAN | Eukaryotic translation initiation factor 3 subunit C | 105 | 5.24 |
| sp\|O43164\|PJA2_HUMAN | E3 ubiquitin-protein ligase Praja-2 | 76 | 5.18 |
| sp\|P18085\|ARF4_HUMAN | ADP-ribosylation factor 4 | 21 | 5.09 |
| sp\|F8VRH0\|PCBP2_HUMAN | Poly(rC)-binding protein 2 | 32 | 5.07 |
| sp\|A8MVZ9\|ALDOC_HUMAN | Fructose-bisphosphate aldolase | 36 | 5.07 |
| sp\|P61962\|DCAF7_HUMAN | DDB1- and CUL4-associated factor 7 | 39 | 5.07 |
| sp\|P35579\|MYH9_HUMAN | Myosin-9 | 227 | 5.07 |
| sp\|P41091\|EIF2S3_HUMAN | Eukaryotic translation initiation factor 2 subunit 3 | 51 | 4.62 |
| sp\|Q8N163\|CCAR2_HUMAN | Cell cycle and apoptosis regulator protein 2 | 103 | 4.61 |
| sp\|P26640\|VARS2_HUMAN | Valine--tRNA ligase | 140 | 4.61 |
| sp\|P15927\|RPA2_HUMAN | Replication protein A 32 kDa subunit | 29 | 4.58 |
| sp\|F8WCF6\|ARPC4_HUMAN | Actin-related protein 2/3 complex subunit 4 | 21 | 4.26 |
| sp\|Q86UY0\|TXNDC5_HUMAN | TXNDC5 protein | 40 | 4.25 |
| sp\|P35249\|RFC4_HUMAN | Replication factor C subunit 4 | 40 | 4.14 |
| sp\|A8MUB1\|TUBA4A_HUMAN | Tubulin alpha-4A chain | 48 | 4.05 |
| sp\|G8JLB6\|HNRNPH1_HUMAN | Heterogeneous nuclear ribonucleoprotein H | 51 | 4.05 |
| sp\|C9J2Y9\|POLR2B_HUMAN | DNA-directed RNA polymerase subunit beta | 133 | 4.03 |
| sp\|P55795\|HNRNPH2_HUMAN | Heterogeneous nuclear ribonucleoprotein H2 | 49 | 3.83 |
| sp\|Q15382\|RHEB_HUMAN | GTP-binding protein Rheb | 20 | 3.58 |
| sp\|P23284\|PPIB_HUMAN | Peptidyl-prolyl cis-trans isomerase B | 24 | 3.58 |
| sp\|P62241\|RPS8_HUMAN | 40S ribosomal protein S8 | 24 | 3.58 |
| sp\|Q5VU58\|TPM3_HUMAN | Tropomyosin alpha-3 chain | 29 | 3.58 |
| sp\|P62753\|RPS6_HUMAN | 40S ribosomal protein S6 | 29 | 3.58 |

| **Table S2. Primer sequences for RT-PCR** | |
| --- | --- |
| COX1 forward | 5’-TTTTGCCAAGGAGTGCTAAAGA-3’ |
| COX1 reverse | 5’-AACCCTCTGCACCCAGTTTTC-3’ |
| B2M forward | 5’-GACTTTAAGGGTTACCTGGGTTG-3’ |
| B2M reverse | 5’-TCACATGCGCCTTGATGTCTG-3’ |
| MUC1 forward | 5’ TGTCAGTGCCGCCGAAAGAA 3’ |
| MUC1 reverse | 5’ CTACAAGTTGGCAGAAGTGG 3’ |
| ATAD3A forward | 5’-GAGCAGGCACCCCAGTTAAT-3’ |
| ATAD3A reverse | 5’-ATTTGTGGTTGGGTCAGGGG-3’ |
| 18S rRNA forward | 5’-GTAACCCGTTGAACCCCATT-3’ |
| 18S rRNA reverse | 5’-CCATCCAATCGGTAGTAGCG-3’ |
