# Supplementary material for: The oncoprotein MUC1 facilitates breast cancer progression by promoting Pink1-dependent mitophagy via ATAD3A destabilization
Source: Cell Death Dis. 2022 Oct 26;13(10):899. doi: 10.1038/s41419-022-05345-z (PMC9606306; doi:10.1038/s41419-022-05345-z)

# Full length uncropped original western blot

**Figure 1**

**Fig. 1A**

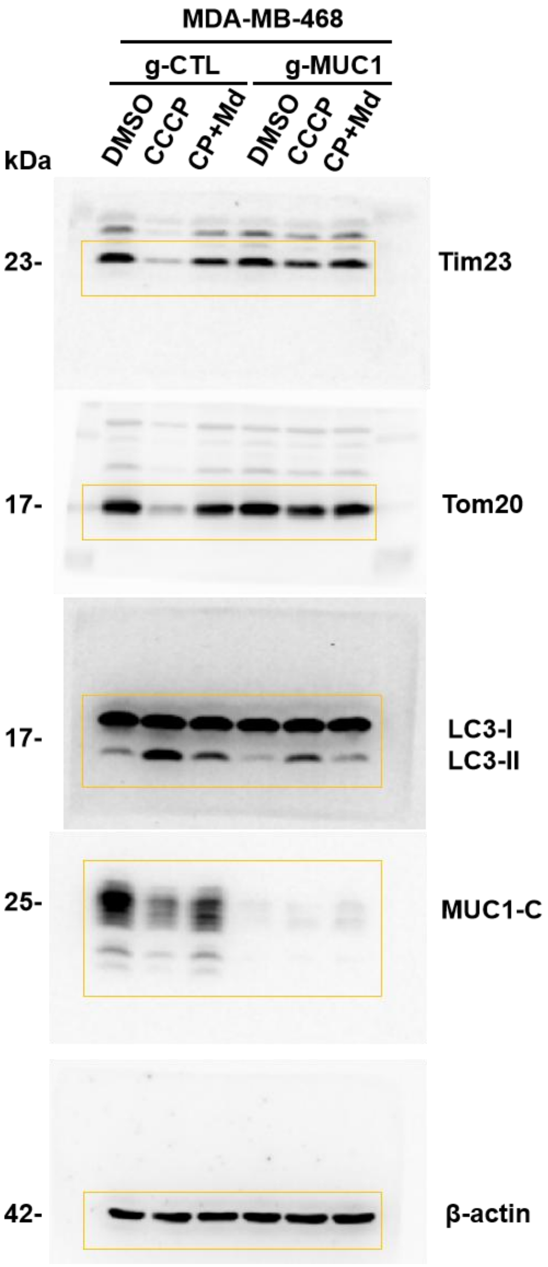

Fig. 1B

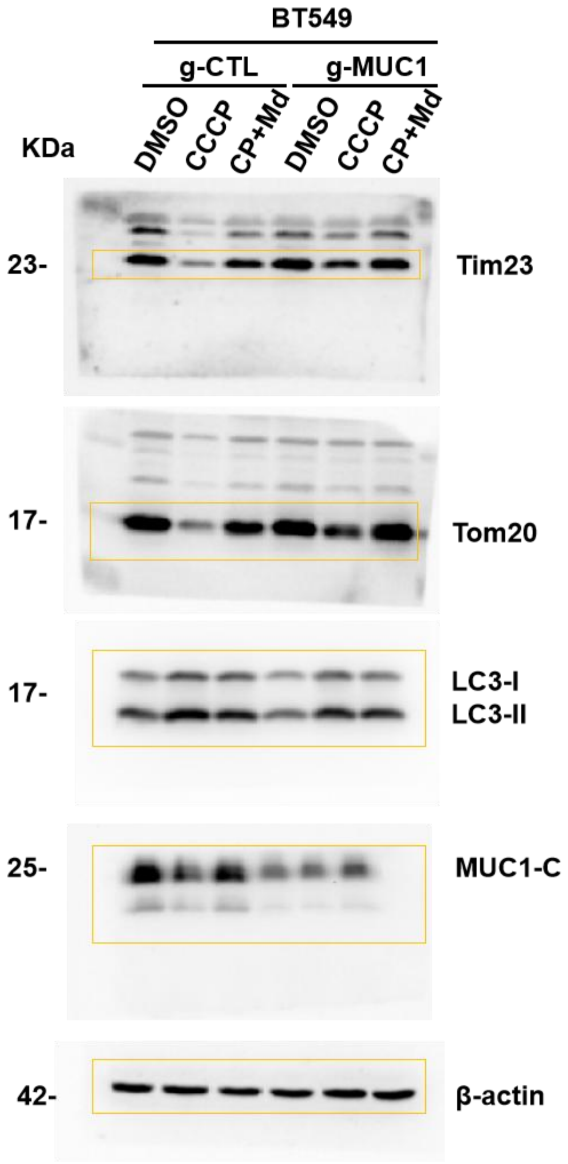

BT549
g-CTL
g-MUC1
DMSO
CCCP
CP+Md
DMSO
CCCP
CP+Md
KDa
23-
Tim23
17-
Tom20
17-
LC3-I
LC3-II
25-
MUC1-C
42-
β-actin

Fig. 1C

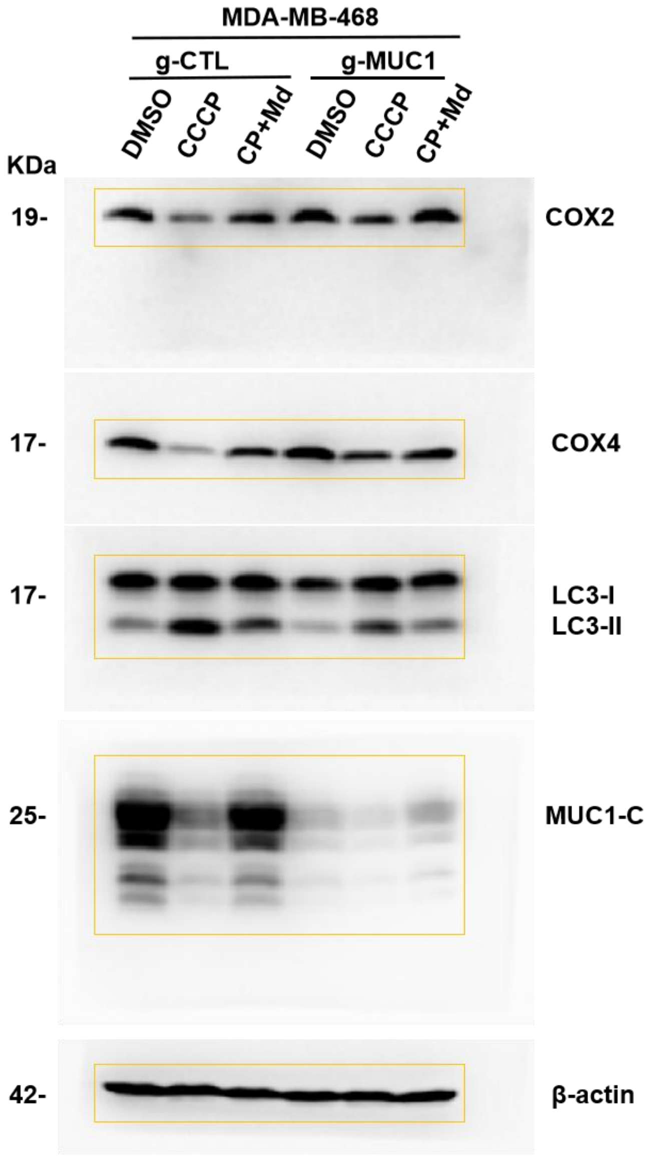
MDA-MB-468
g-CTL
g-MUC1
DMSO
CCCP
CP+Md
DMSO
CCCP
CP+Md
KDa
19-
COX2
17-
COX4
17-
LC3-I
LC3-II
25-
MUC1-C
42-
β-actin

Fig. 1D

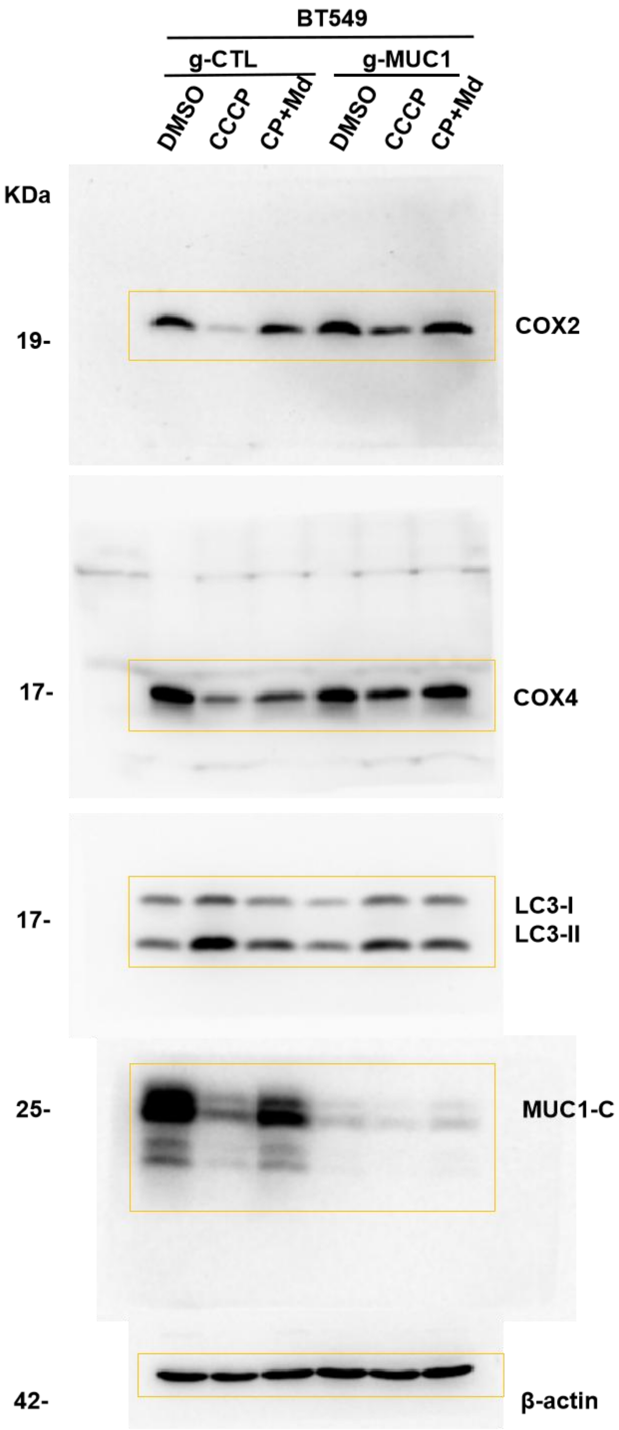
BT549
g-CTL
g-MUC1
DMSO
CCCP
CP+Md
DMSO
CCCP
CP+Md
KDa
19-
COX2
17-
COX4
17-
LC3-I
LC3-II
25-
MUC1-C
42-
β-actin

Fig. 1E

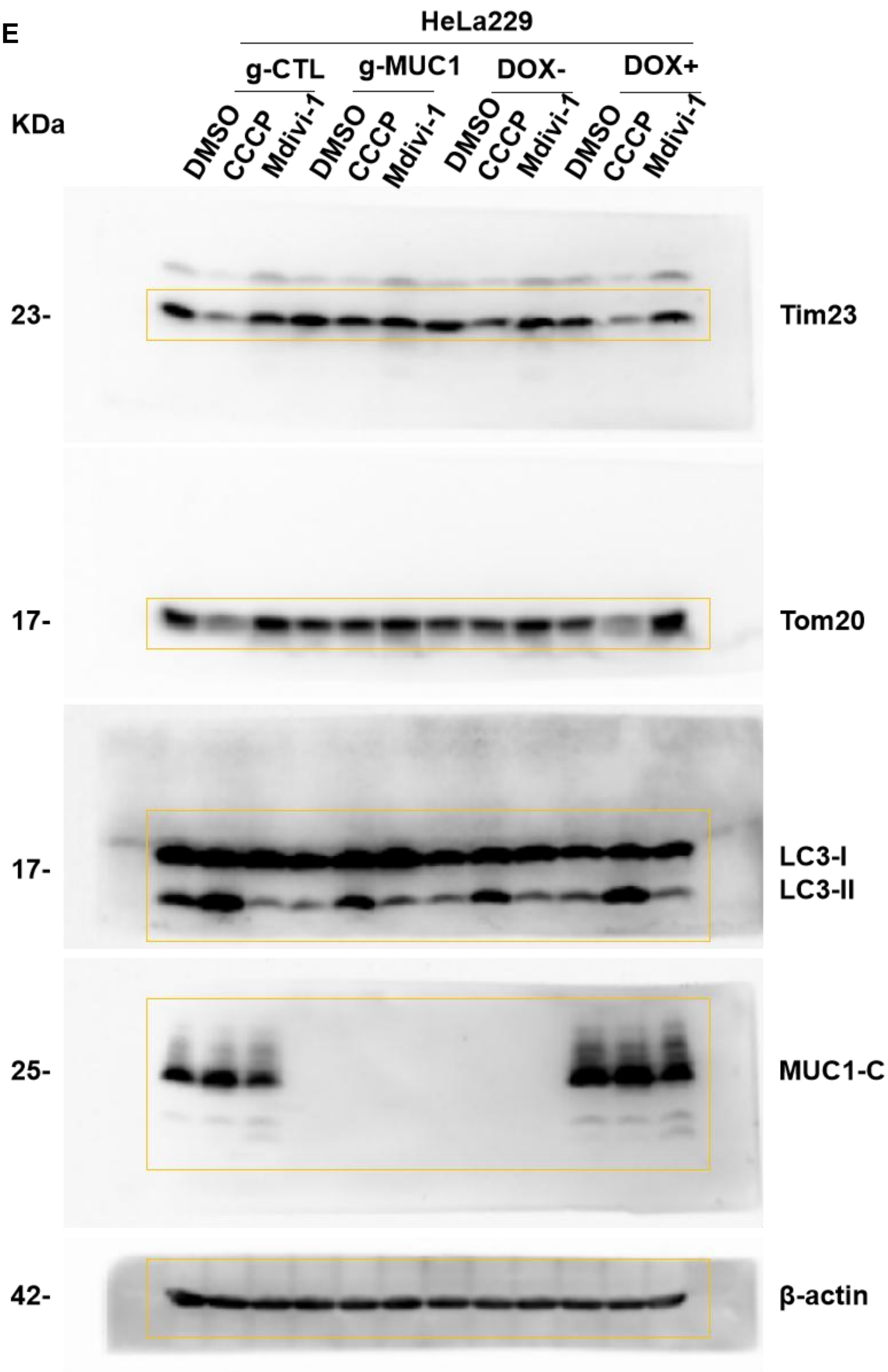
HeLa229
g-CTL
g-MUC1
DOX-
DOX+
KDa
DMSO
CCCP
Mdivi-1
DMSO
CCCP
Mdivi-1
DMSO
CCCP
Mdivi-1
DMSO
CCCP
Mdivi-1
23-
Tim23
17-
Tom20
17-
LC3-I
LC3-II
25-
MUC1-C
42-
β-actin

Fig. 1L

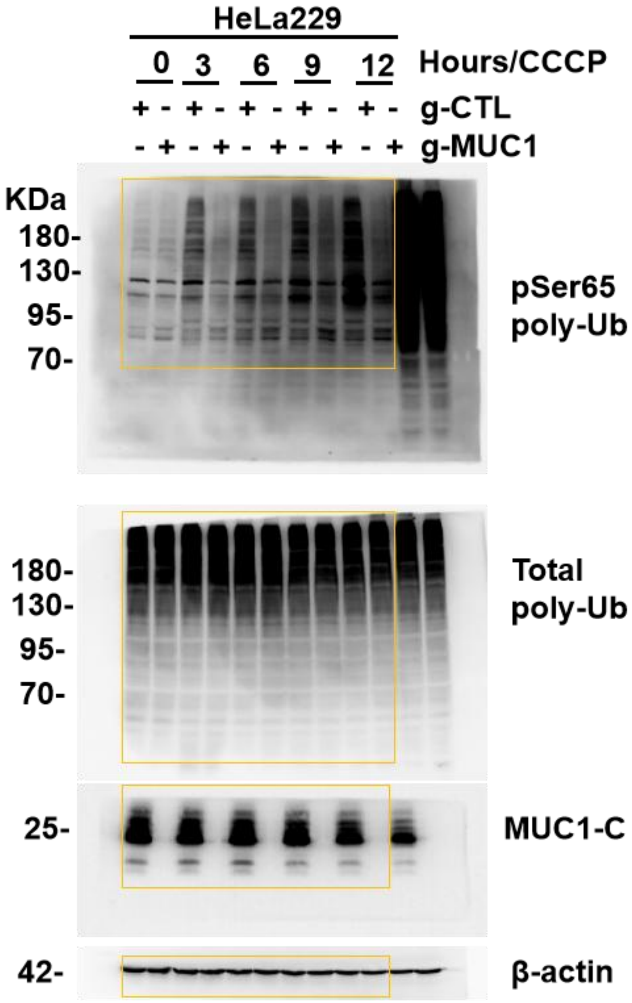
HeLa229
0 3 6 9 12 Hours/CCCP
+ - + - + - + - + - g-CTL
- + - + - + - + - + g-MUC1
KDa
180-
130-
95-
70-
pSer65 poly-Ub
180-
130-
95-
70-
Total poly-Ub
25-
MUC1-C
42-
β-actin

**Figure 2**

**Fig. 2B**

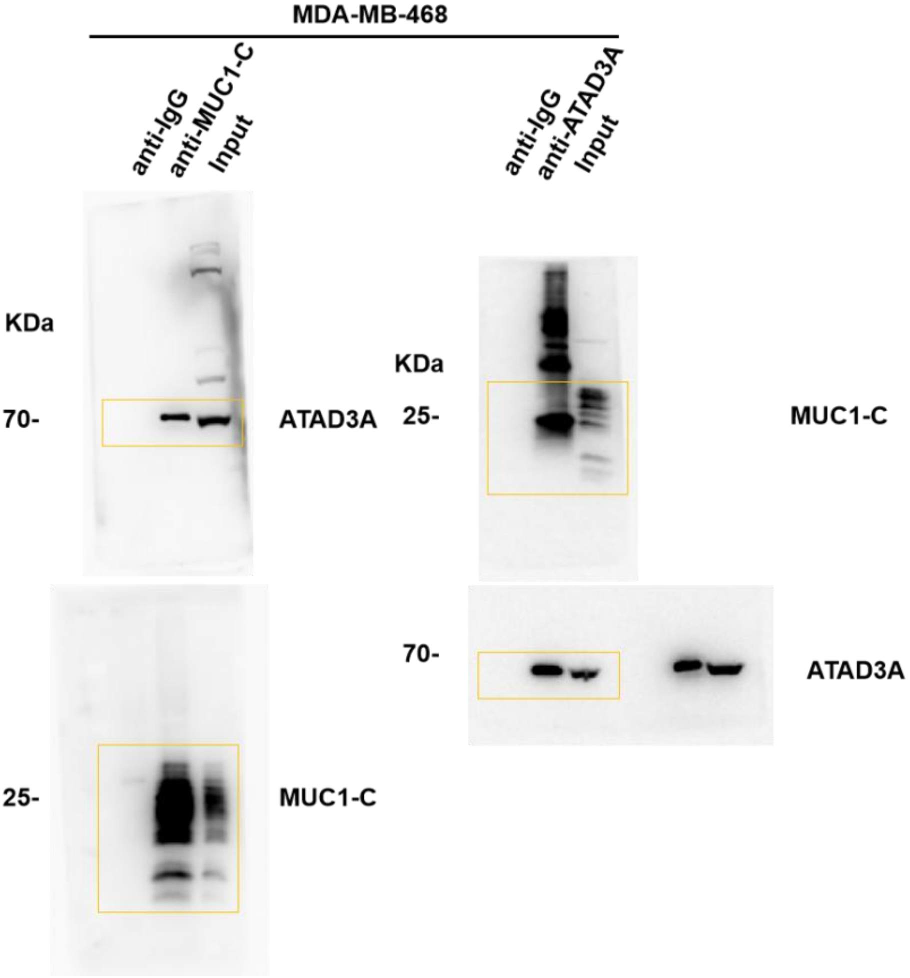

Fig. 2C

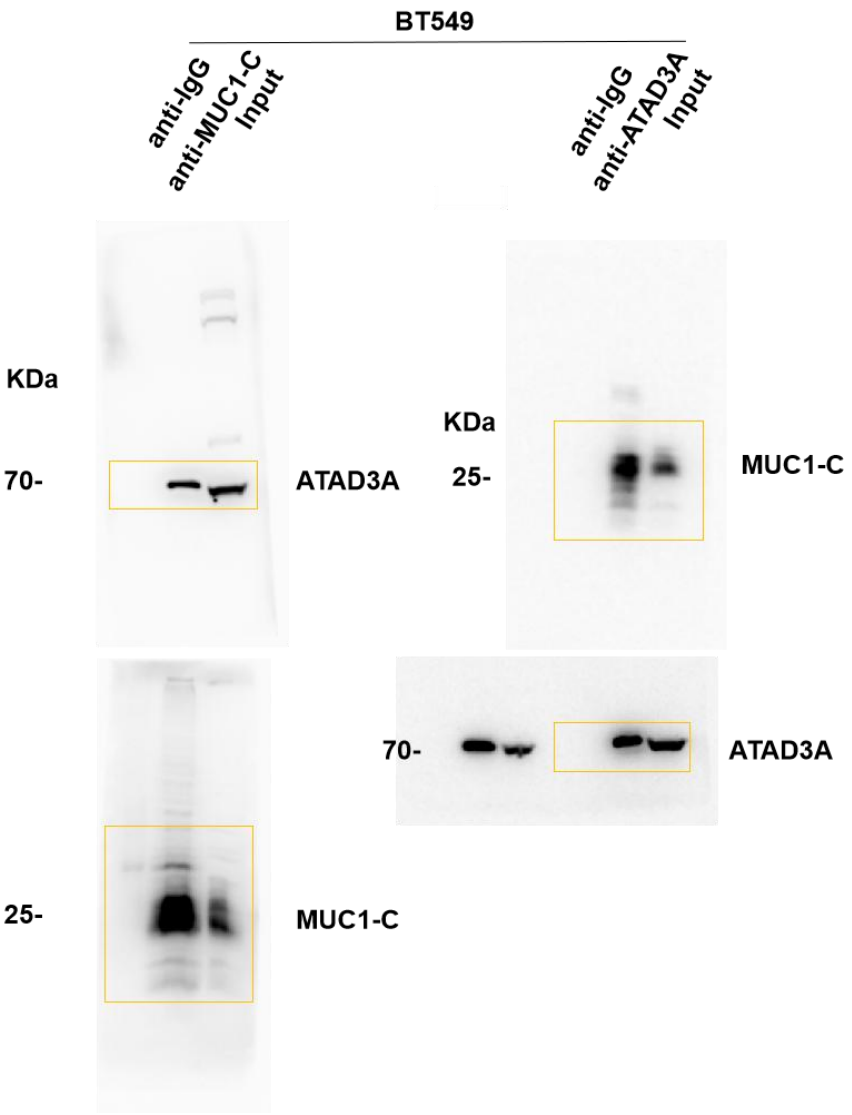
BT549
anti-IgG
anti-MUC1-C
Input
anti-IgG
anti-ATAD3A
Input
KDa
70-
ATAD3A
KDa
25-
MUC1-C
70-
ATAD3A
25-
MUC1-C

Fig. 2D

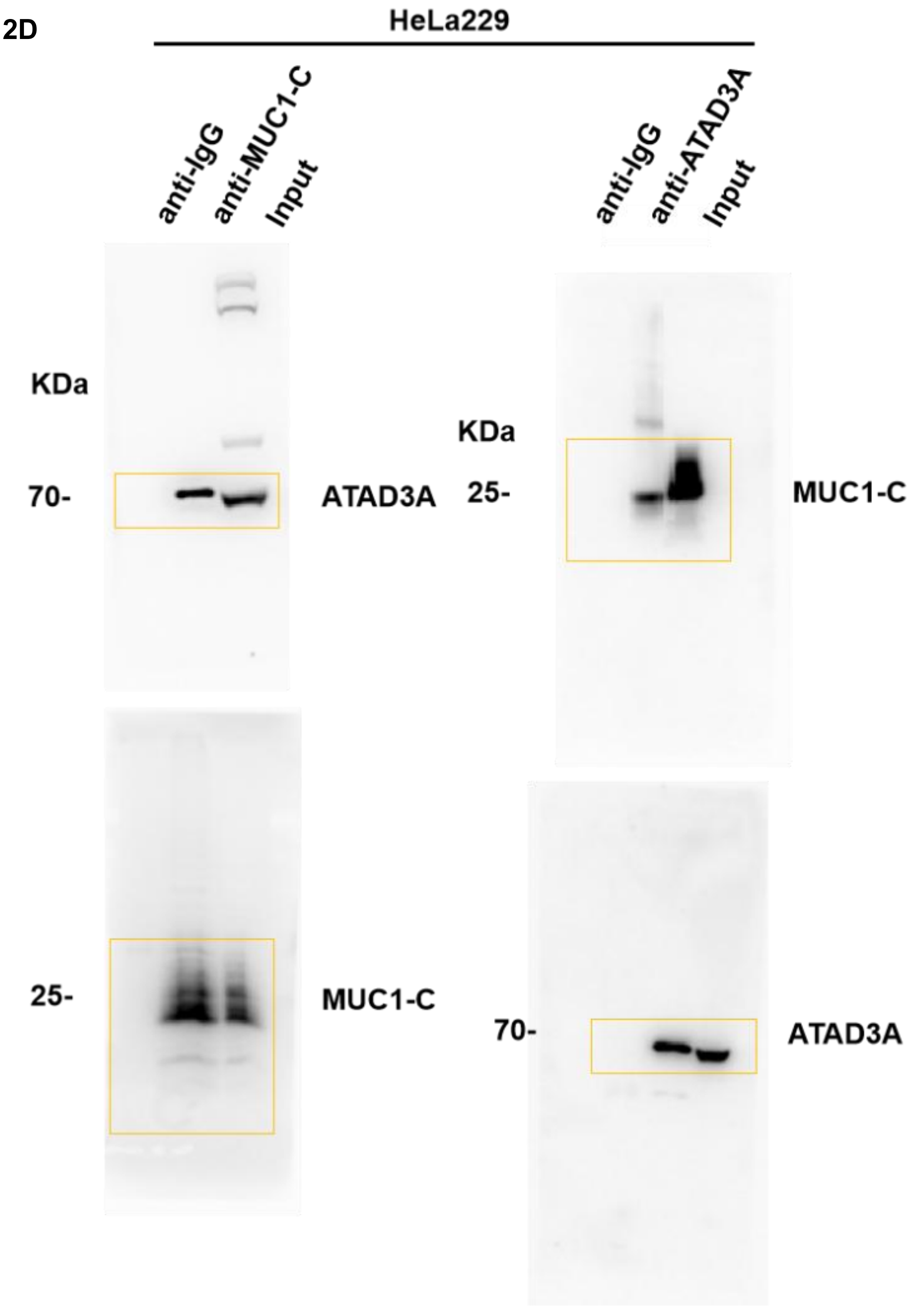
HeLa229
anti-IgG
anti-MUC1-C
Input
anti-IgG
anti-ATAD3A
Input
KDa
70-
ATAD3A
KDa
25-
MUC1-C
25-
MUC1-C
70-
ATAD3A

Fig. 2E

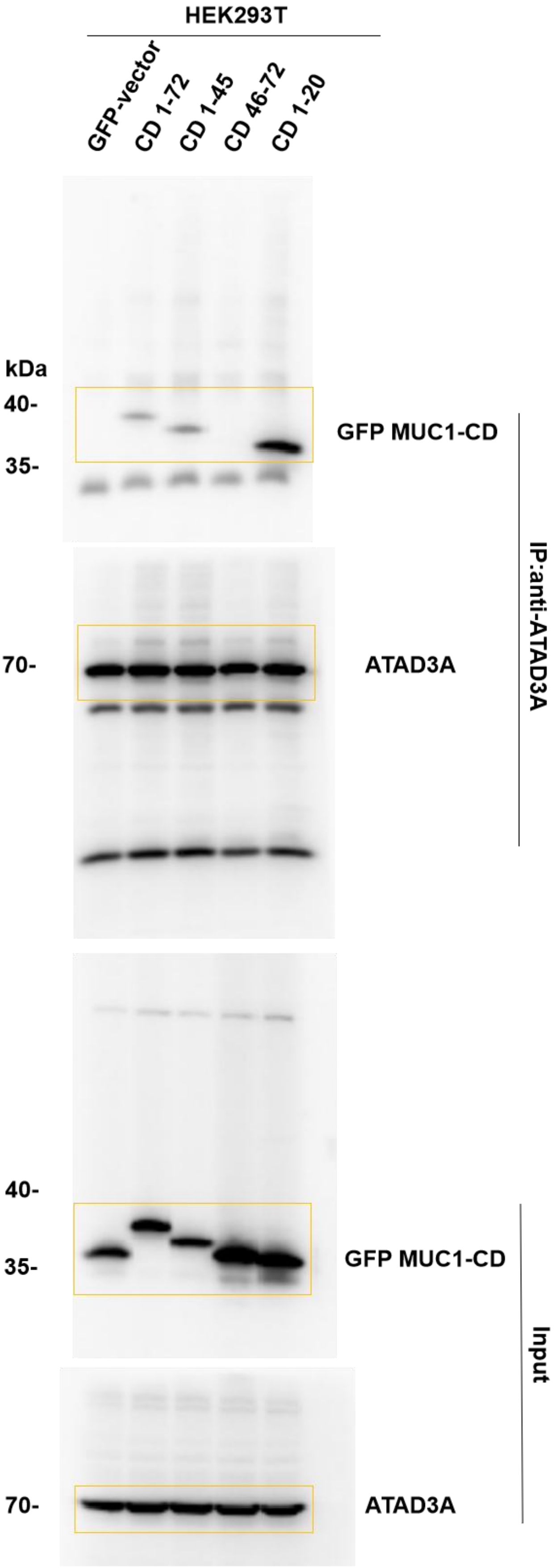
HEK293T
GFP-vector
CD 1-72
CD 1-45
CD 46-72
CD 1-20
kDa
40-
35-
GFP MUC1-CD
70-
ATAD3A
IP:anti-ATAD3A
40-
35-
GFP MUC1-CD
70-
ATAD3A
Input

Fig. 2F

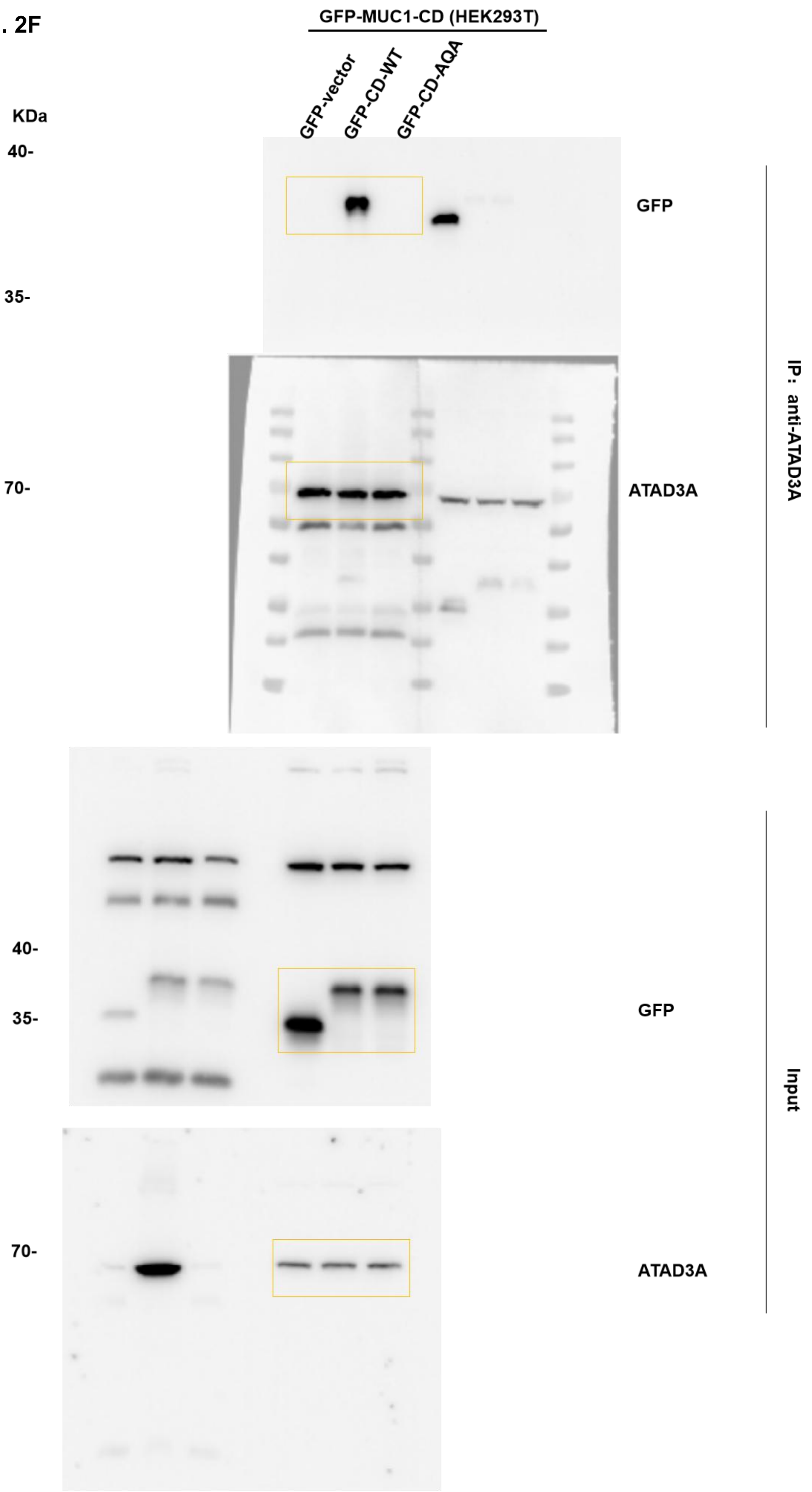
GFP-MUC1-CD (HEK293T)
GFP-vector
GFP-CD-WT
GFP-CD-AQA
KDa
40-
35-
GFP
70-
ATAD3A
IP: anti-ATAD3A
40-
35-
GFP
70-
ATAD3A
Input

Fig. 2G

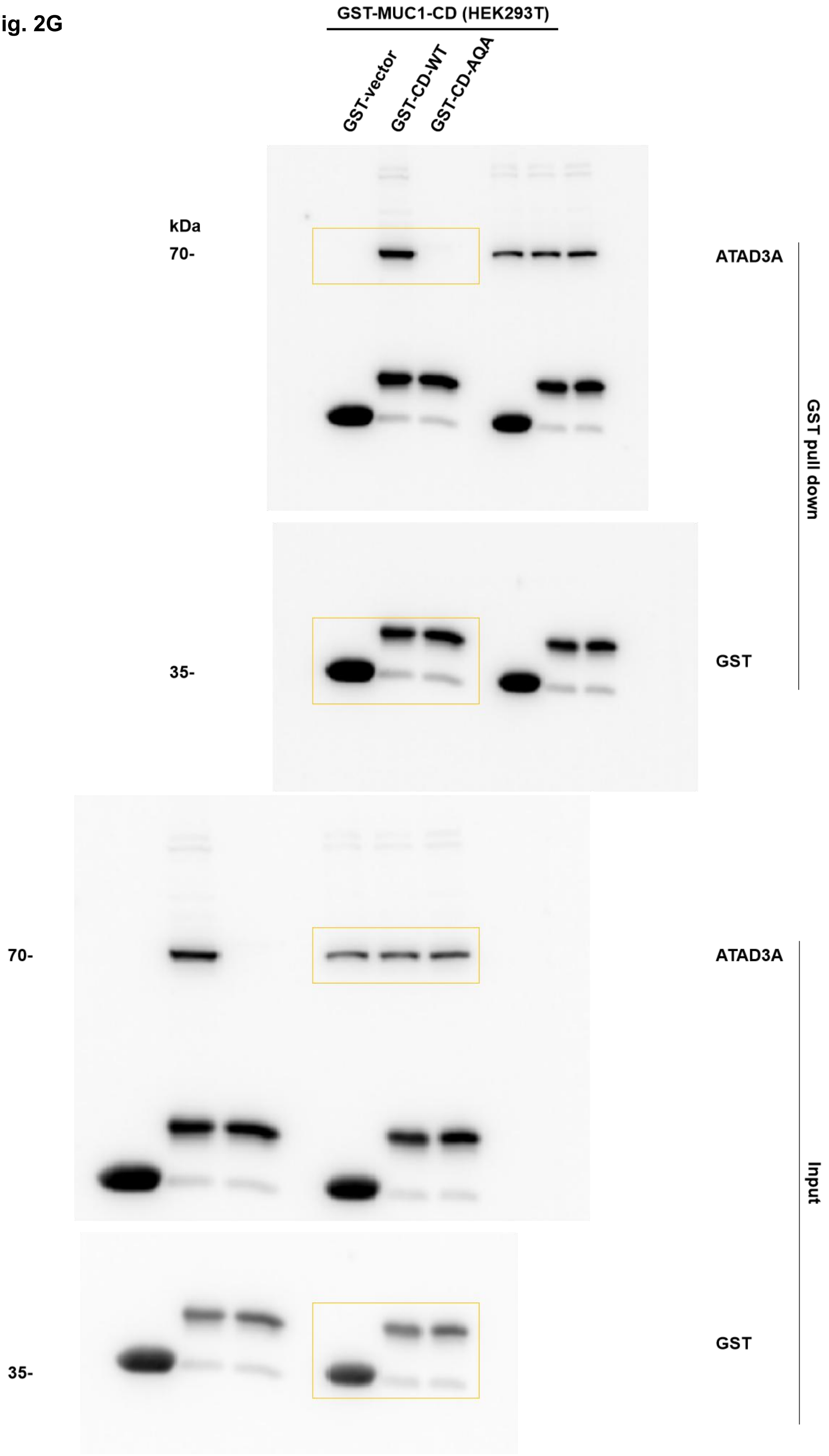
GST-MUC1-CD (HEK293T)
GST-vector
GST-CD-WT
GST-CD-AQA
kDa
70-
35-
70-
35-
ATAD3A
GST
ATAD3A
GST
GST pull down
Input

Fig. 2H

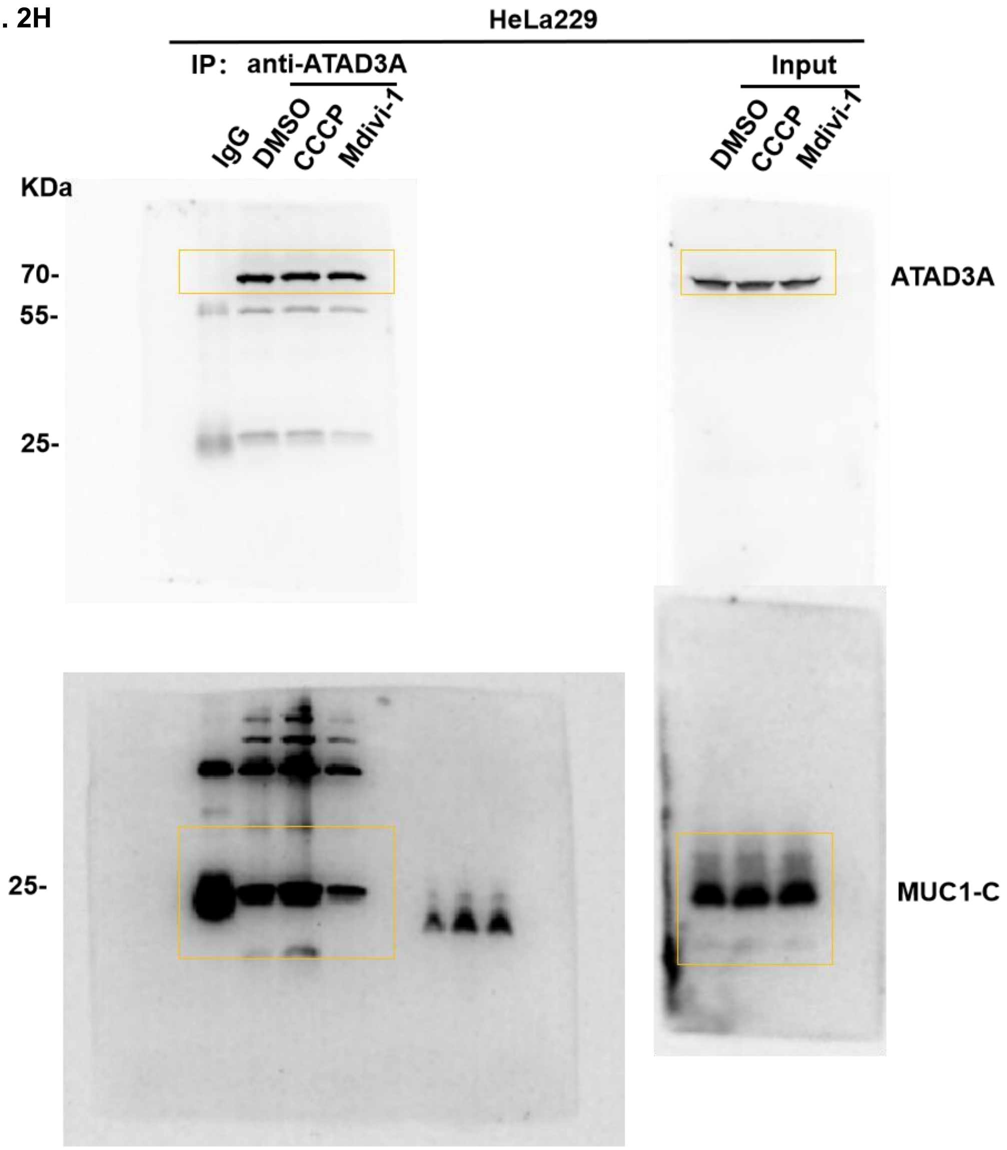
HeLa229
IP： anti-ATAD3A
Input
IgG
DMSO
CCCP
Mdivi-1
DMSO
CCCP
Mdivi-1
KDa
70-
55-
25-
ATAD3A
25-
MUC1-C

Fig. 2I

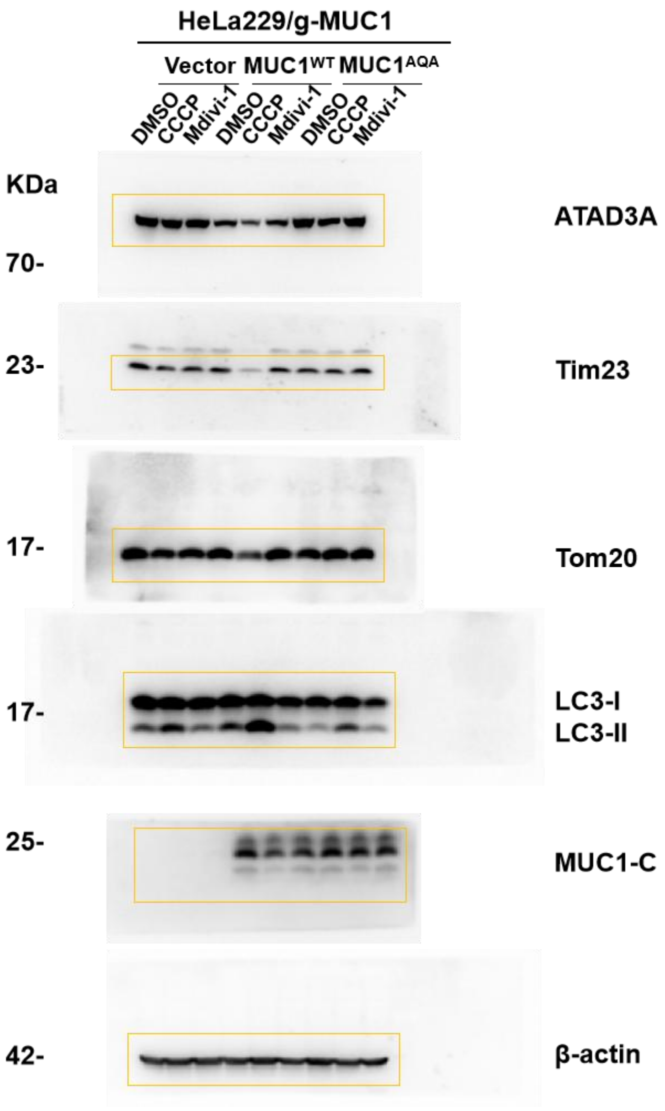
HeLa229/g-MUC1
Vector
MUC1WT
MUC1AQA
DMSO
CCCP
Mdivi-1
DMSO
CCCP
Mdivi-1
DMSO
CCCP
Mdivi-1
KDa
70-
ATAD3A
23-
Tim23
17-
Tom20
17-
LC3-I
LC3-II
25-
MUC1-C
42-
β-actin

**Figure 3**

**Fig. 3A**

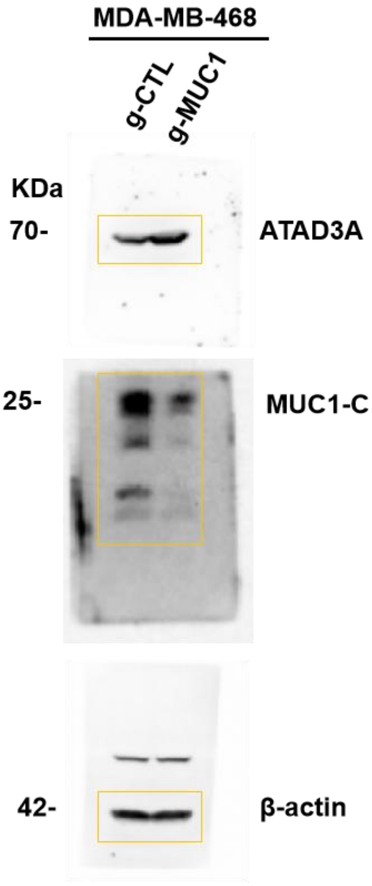


**Fig. 3B**

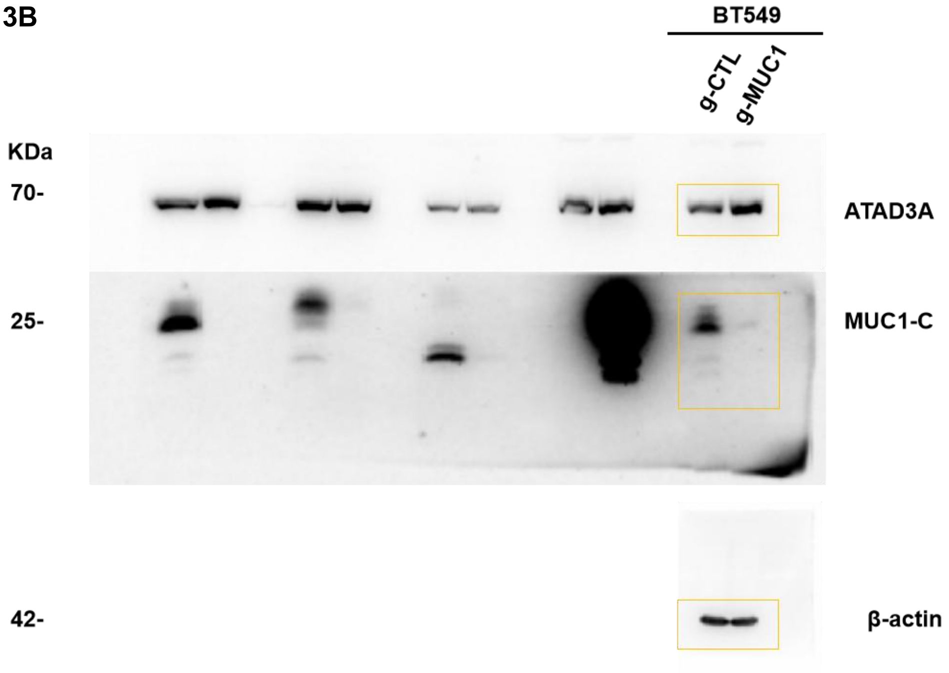

Fig. 3C

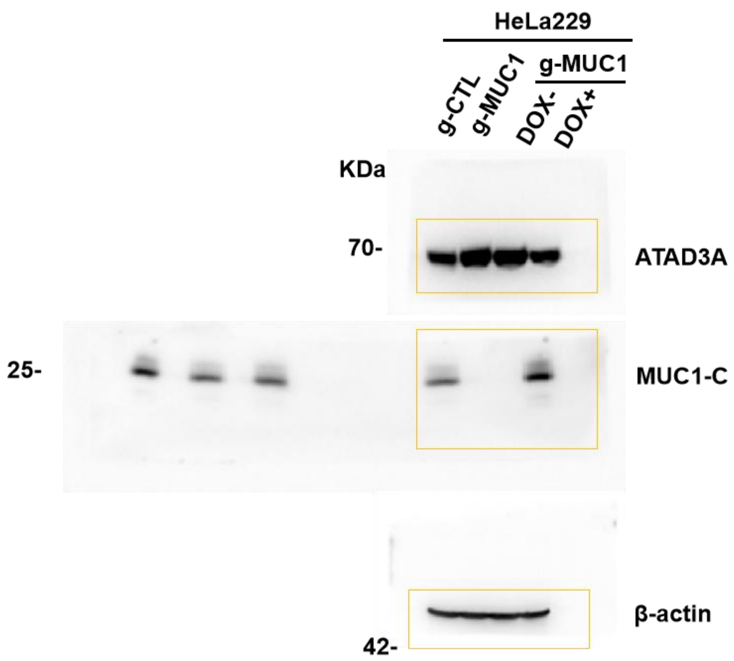
HeLa229
g-MUC1
g-CTL
g-MUC1
DOX-
DOX+
KDa
70-
ATAD3A
25-
MUC1-C
β-actin
42-

Fig. 3D

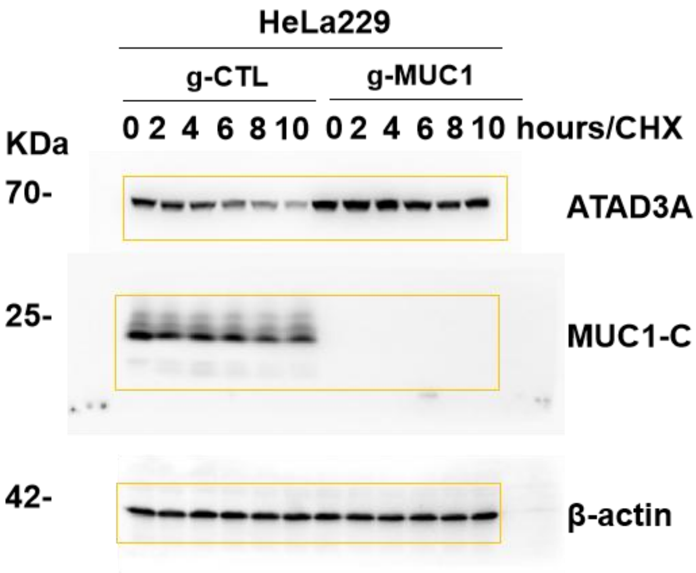
HeLa229
g-CTL
g-MUC1
0 2 4 6 8 10 0 2 4 6 8 10 hours/CHX
KDa
70-
ATAD3A
25-
MUC1-C
42-
β-actin

Fig. 3E

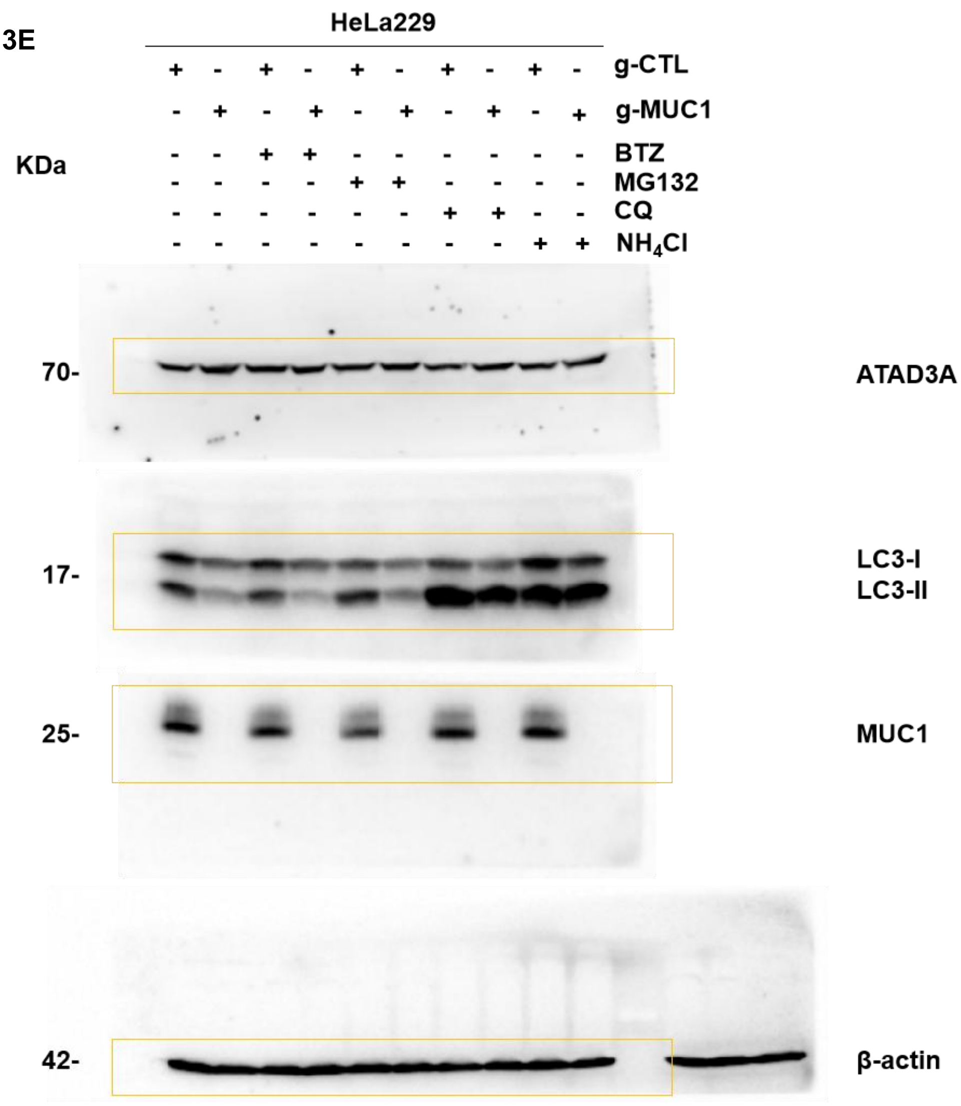
HeLa229
+ - + - + - + - + - g-CTL
- + - + - + - + - + g-MUC1
KDa
- - + + - - - - - - BTZ
- - - - + + - - - - MG132
- - - - - - + + - - CQ
- - - - - - - - + + NH4Cl
70-
ATAD3A
17-
LC3-I
LC3-II
25-
MUC1
42-
β-actin

Fig. 3F

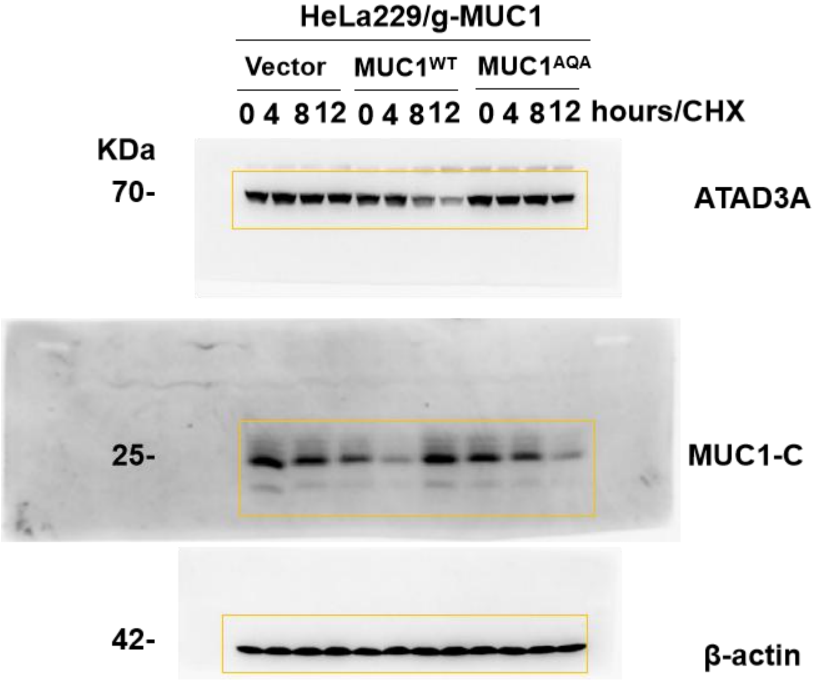
HeLa229/g-MUC1
Vector
MUC1WT
MUC1AQA
0 4 8 12 0 4 8 12 0 4 8 12 hours/CHX
KDa
70-
ATAD3A
25-
MUC1-C
42-
β-actin

Fig. 3G

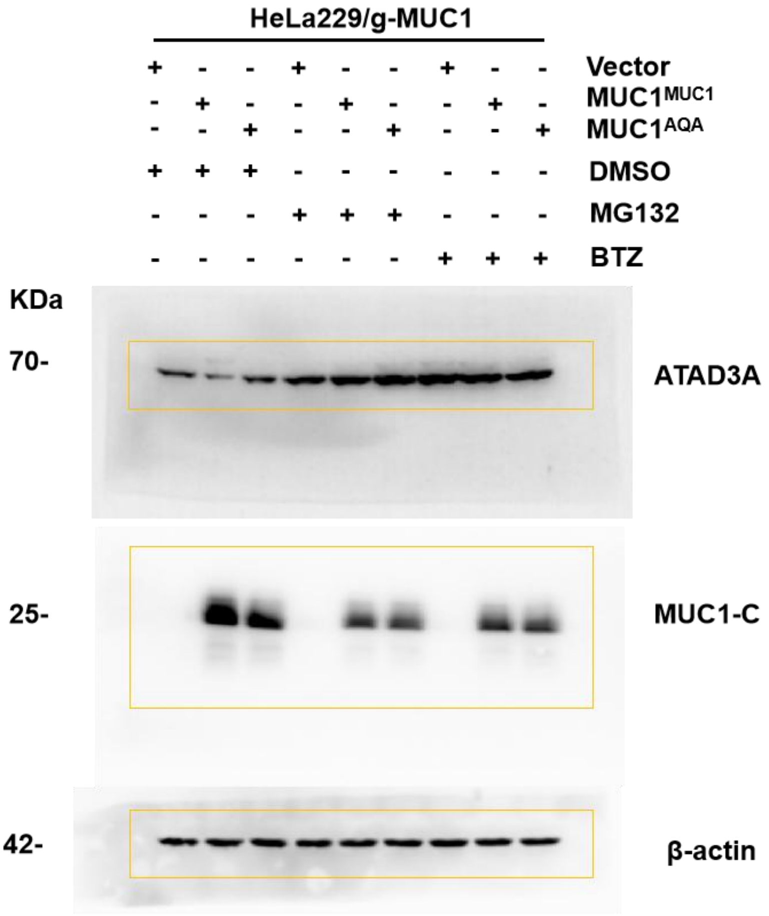

HeLa229/g-MUC1
+ - - + - - + - - Vector
- + - - + - - + - MUC1MUC1
- - + - - + - - + MUC1AQA
+ + + - - - - - - DMSO
- - - + + + - - - MG132
- - - - - - + + + BTZ
KDa
70-
ATAD3A
25-
MUC1-C
42-
β-actin

Fig. 3H

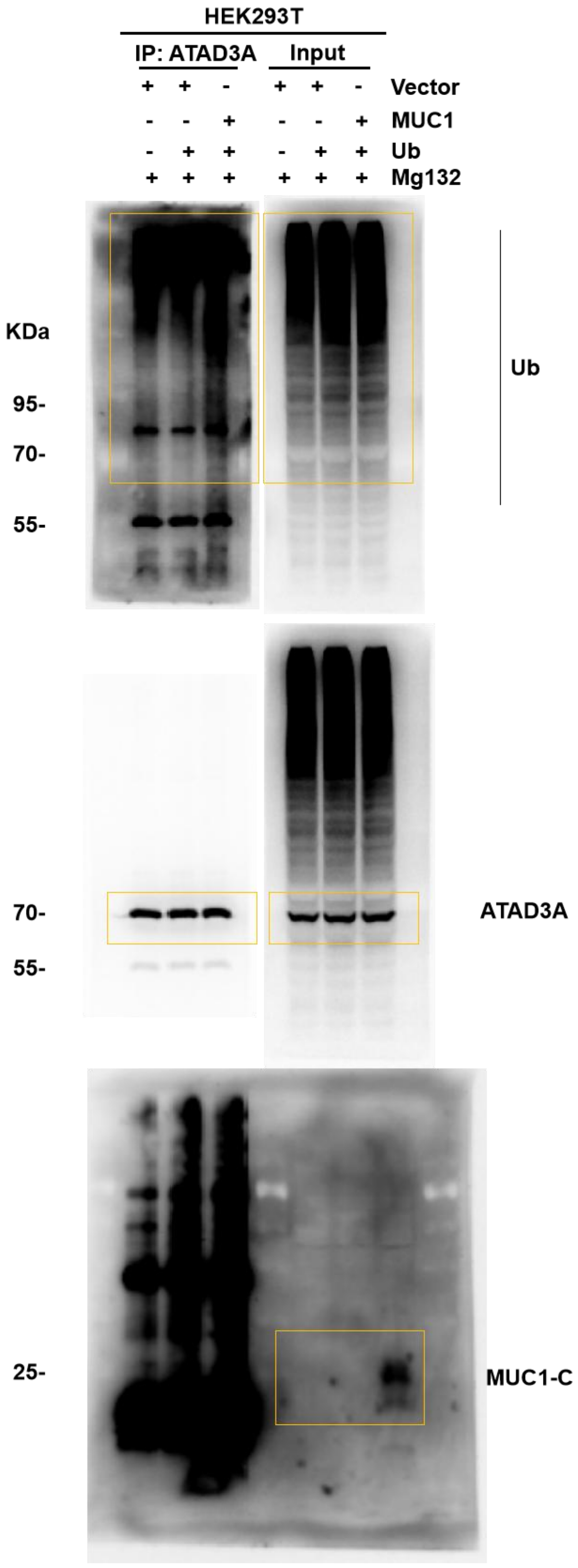
HEK293T
IP: ATAD3A
Input
+ + - + + - Vector
- - + - - + MUC1
- + + - + + Ub
+ + + + + + Mg132
KDa
95-
70-
55-
Ub
70-
55-
ATAD3A
25-
MUC1-C

Fig. 3I

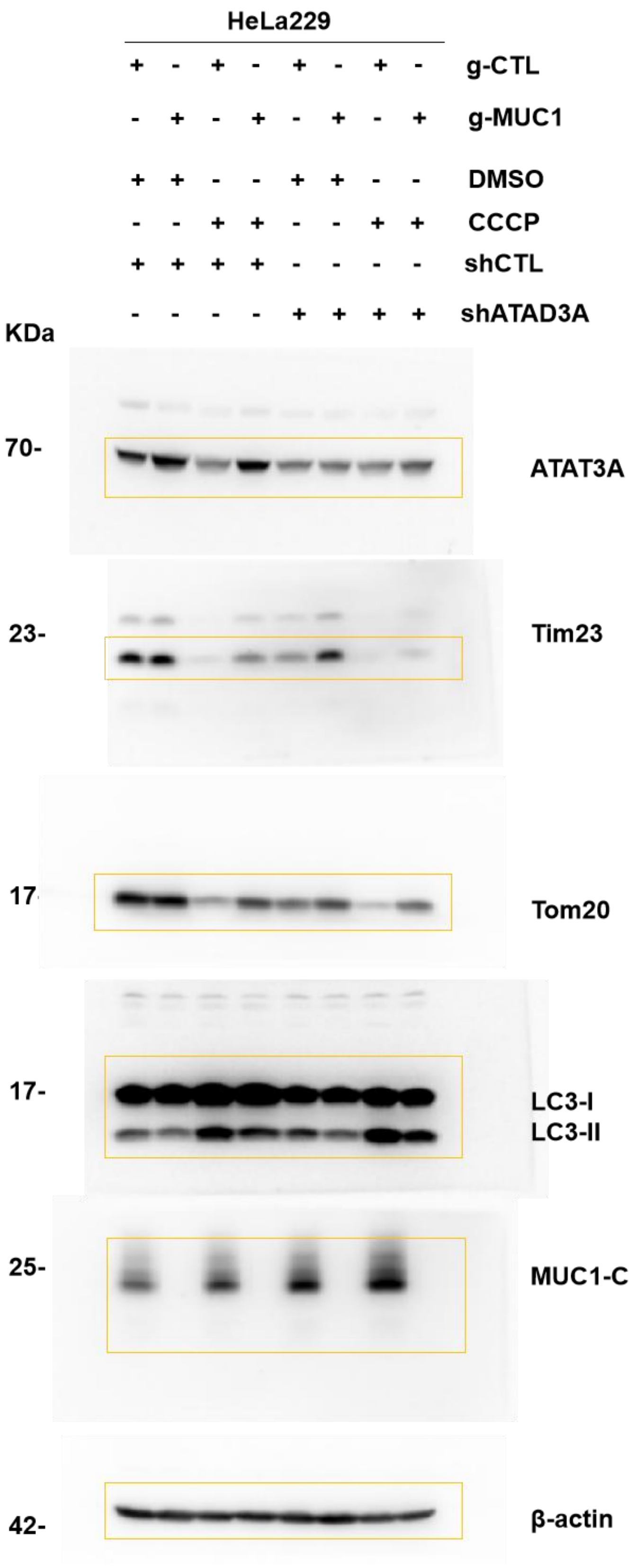
HeLa229
+ - + - + - + - g-CTL
- + - + - + - + g-MUC1
+ + - - + + - - DMSO
- - + + - - + + CCCP
+ + + + - - - - shCTL
- - - - + + + + shATAD3A
KDa
70-
ATAT3A
23-
Tim23
17-
Tom20
17-
LC3-I
LC3-II
25-
MUC1-C
42-
β-actin

Fig. 3J

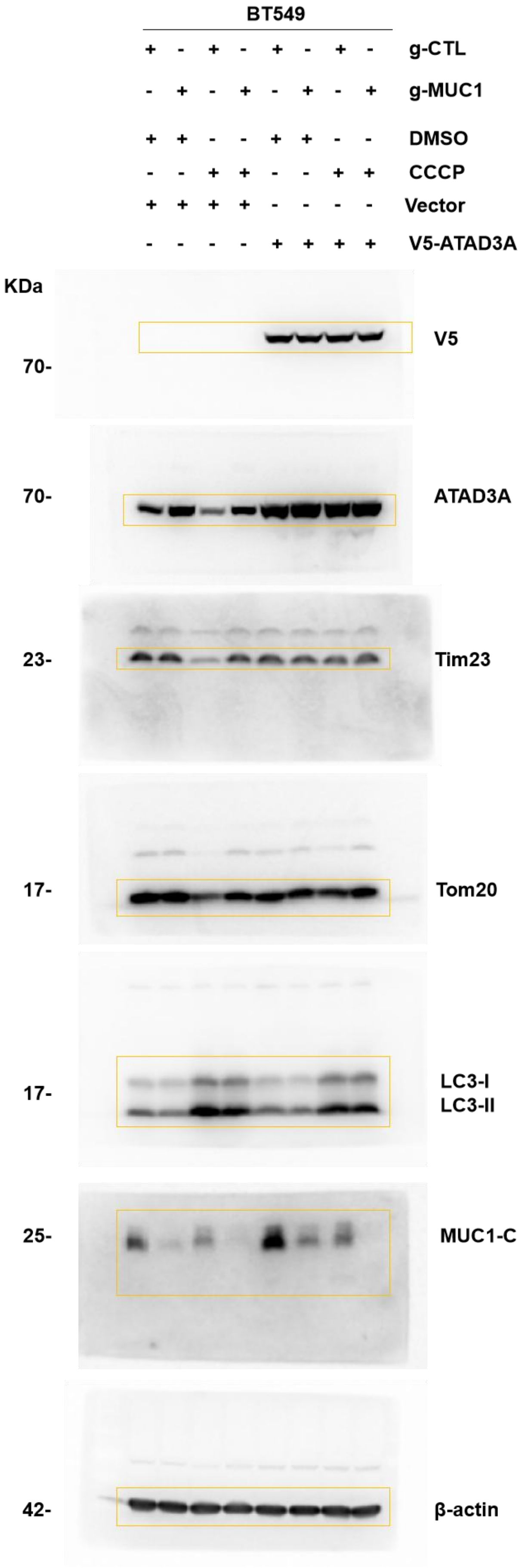

BT549
+ - + - + - + - g-CTL
- + - + - + - + g-MUC1
+ + - - + + - - DMSO
- - + + - - + + CCCP
+ + + + - - - - Vector
- - - - + + + + V5-ATAD3A
KDa
70-
V5
70-
ATAD3A
23-
Tim23
17-
Tom20
17-
LC3-I
LC3-II
25-
MUC1-C
42-
β-actin

## Figure 4

Fig. 4A

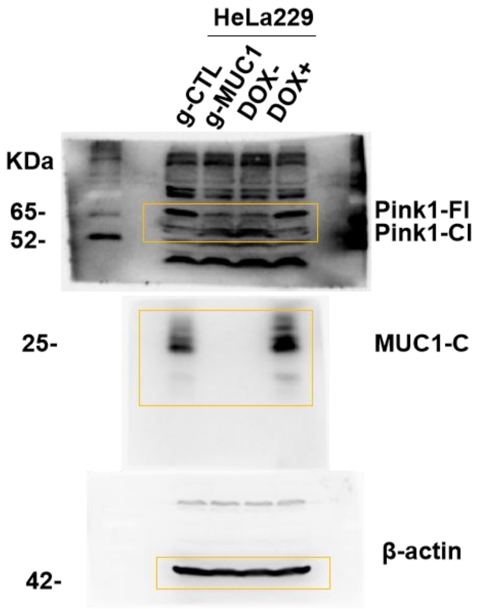


Fig. 4B

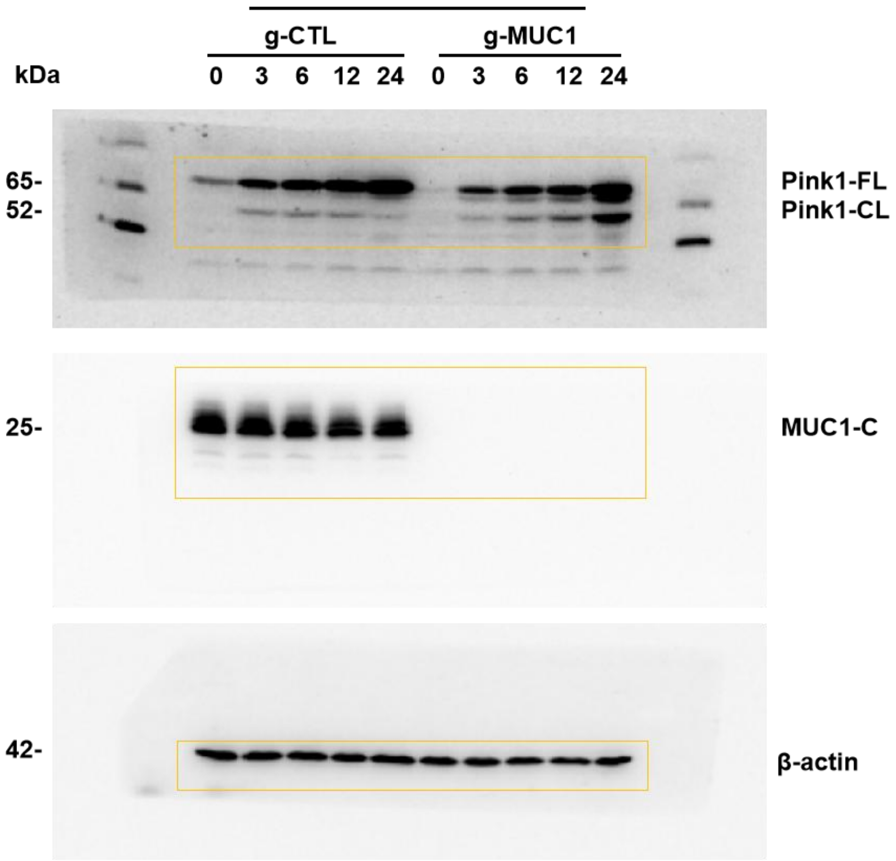

Fig. 4C

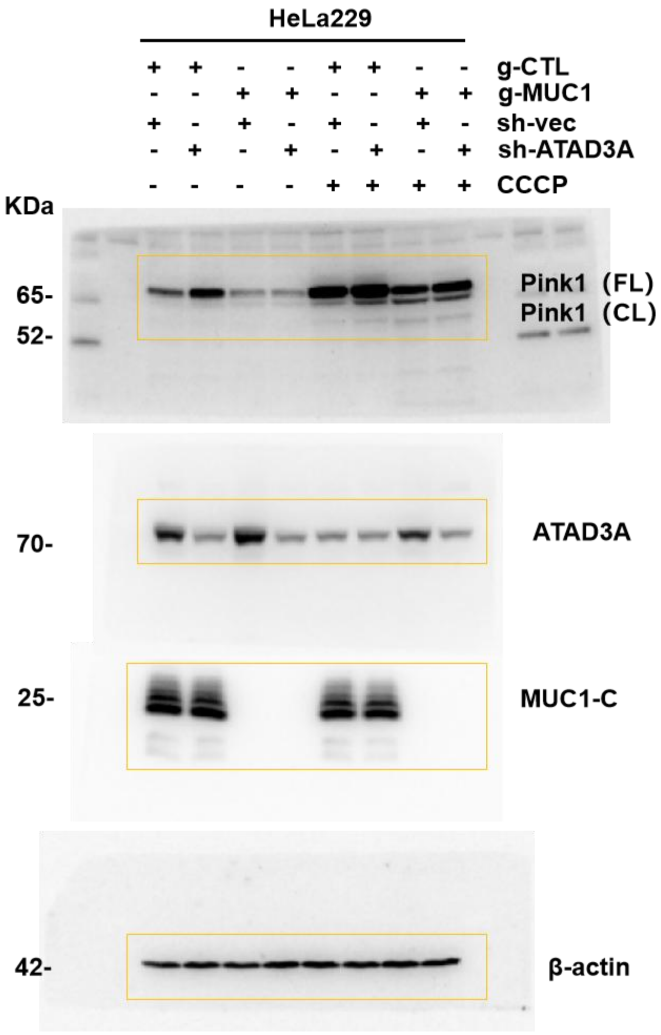

HeLa229
g-CTL
g-MUC1
sh-vec
sh-ATAD3A
CCCP
KDa
65-
52-
Pink1 (FL)
Pink1 (CL)
70-
ATAD3A
25-
MUC1-C
42-
β-actin

Fig. 4D

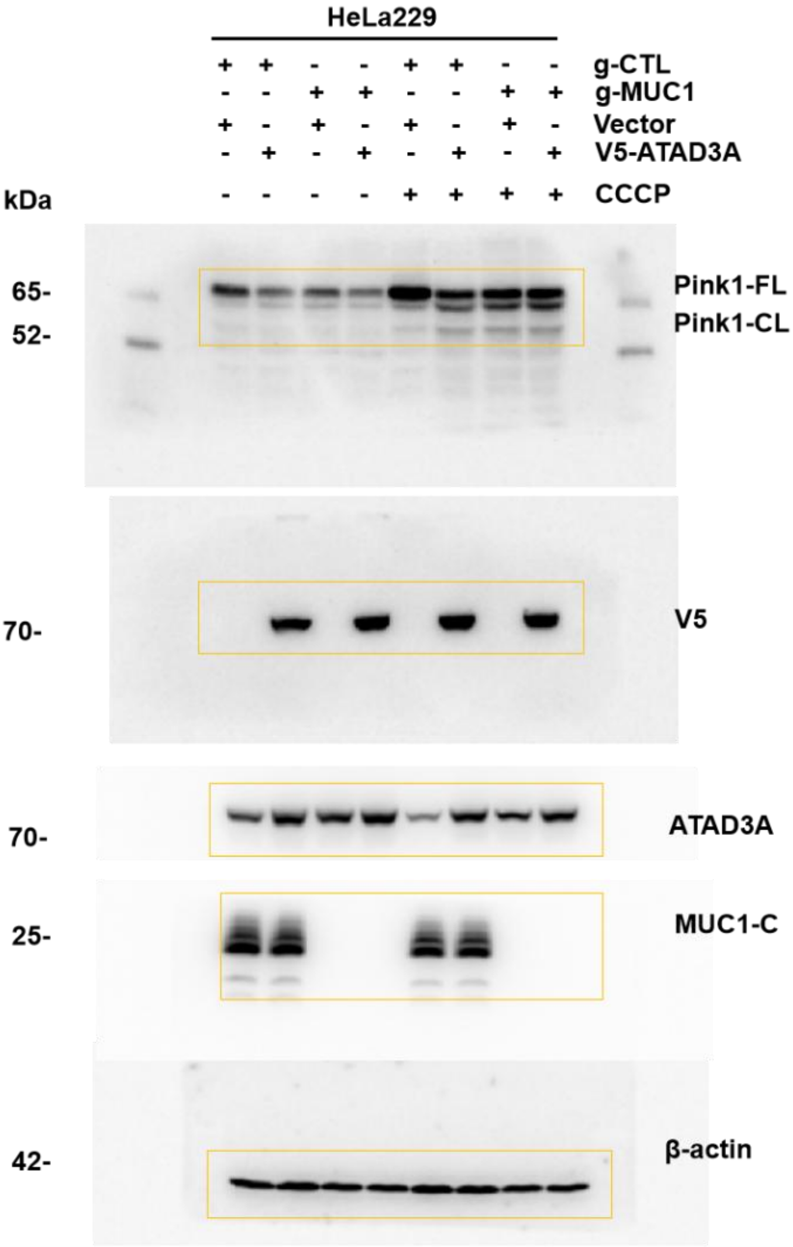
HeLa229
g-CTL
g-MUC1
Vector
V5-ATAD3A
CCCP
kDa
65-
52-
Pink1-FL
Pink1-CL
70-
V5
70-
ATAD3A
25-
MUC1-C
42-
β-actin

Fig. 4E

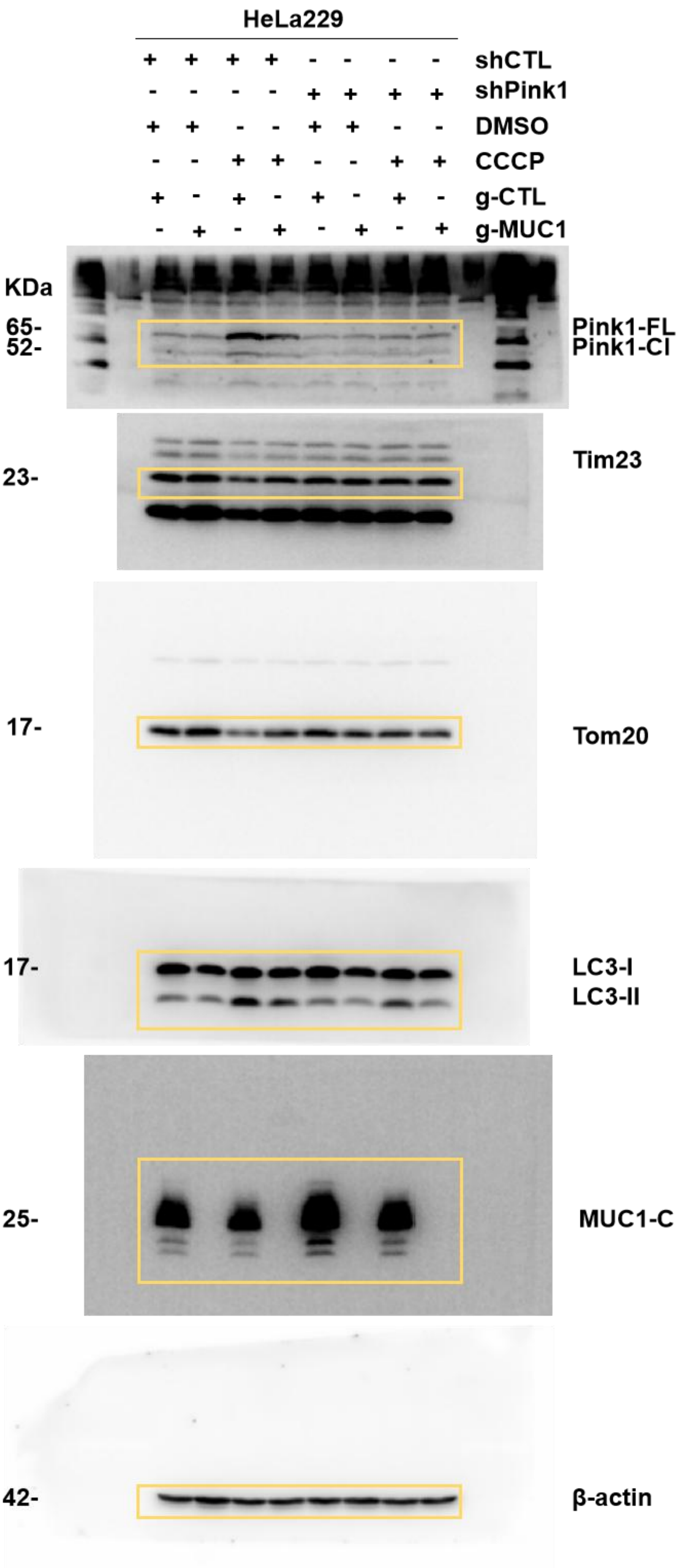
HeLa229
+ + + + - - - - shCTL
- - - - + + + + shPink1
+ + - - + + - - DMSO
- - + + - - + + CCCP
+ - + - + - + - g-CTL
- + - + - + - + g-MUC1
KDa
65-
52-
Pink1-FL
Pink1-Cl
23-
Tim23
17-
Tom20
17-
LC3-I
LC3-II
25-
MUC1-C
42-
β-actin

Fig. 4F

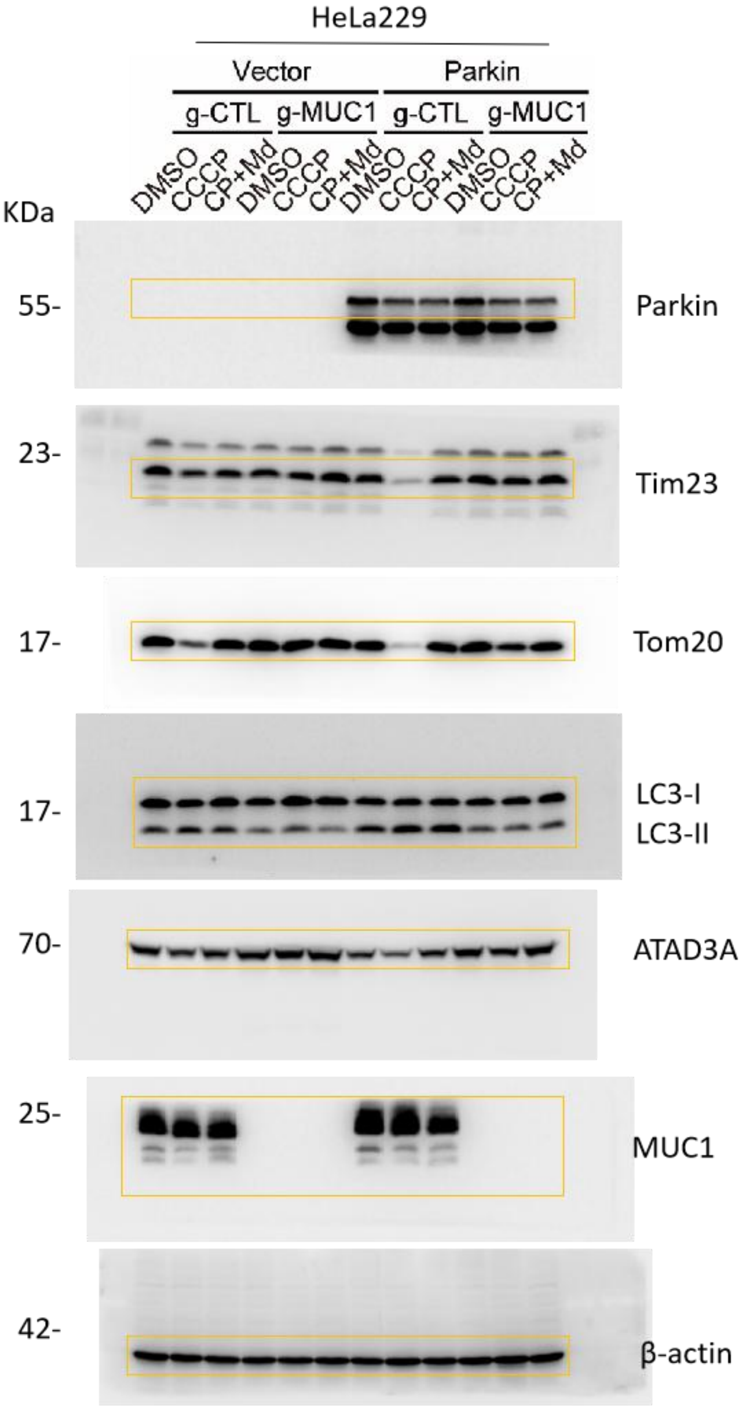
HeLa229
Vector
Parkin
g-CTL
g-MUC1
g-CTL
g-MUC1
DMSO
CCCP
CP+Md
DMSO
CCCP
CP+Md
DMSO
CCCP
CP+Md
DMSO
CCCP
CP+Md
KDa
55-
Parkin
23-
Tim23
17-
Tom20
17-
LC3-I
LC3-II
70-
ATAD3A
25-
MUC1
42-
β-actin

Fig. 4G

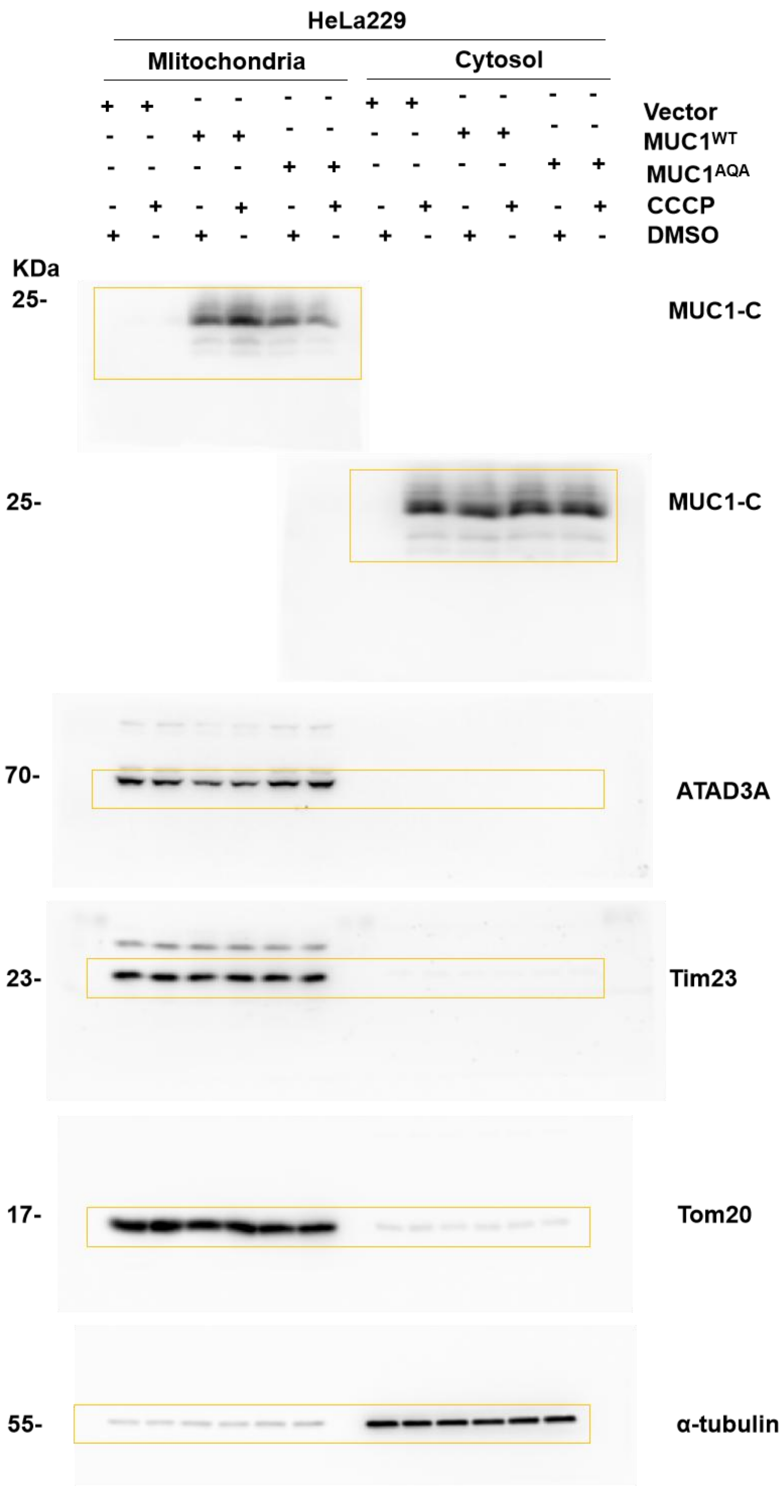
HeLa229
Mlitochondria
Cytosol
Vector
MUC1WT
MUC1AQA
CCCP
DMSO
KDa
25-
MUC1-C
25-
MUC1-C
70-
ATAD3A
23-
Tim23
17-
Tom20
55-
α-tubulin

Fig. 4H

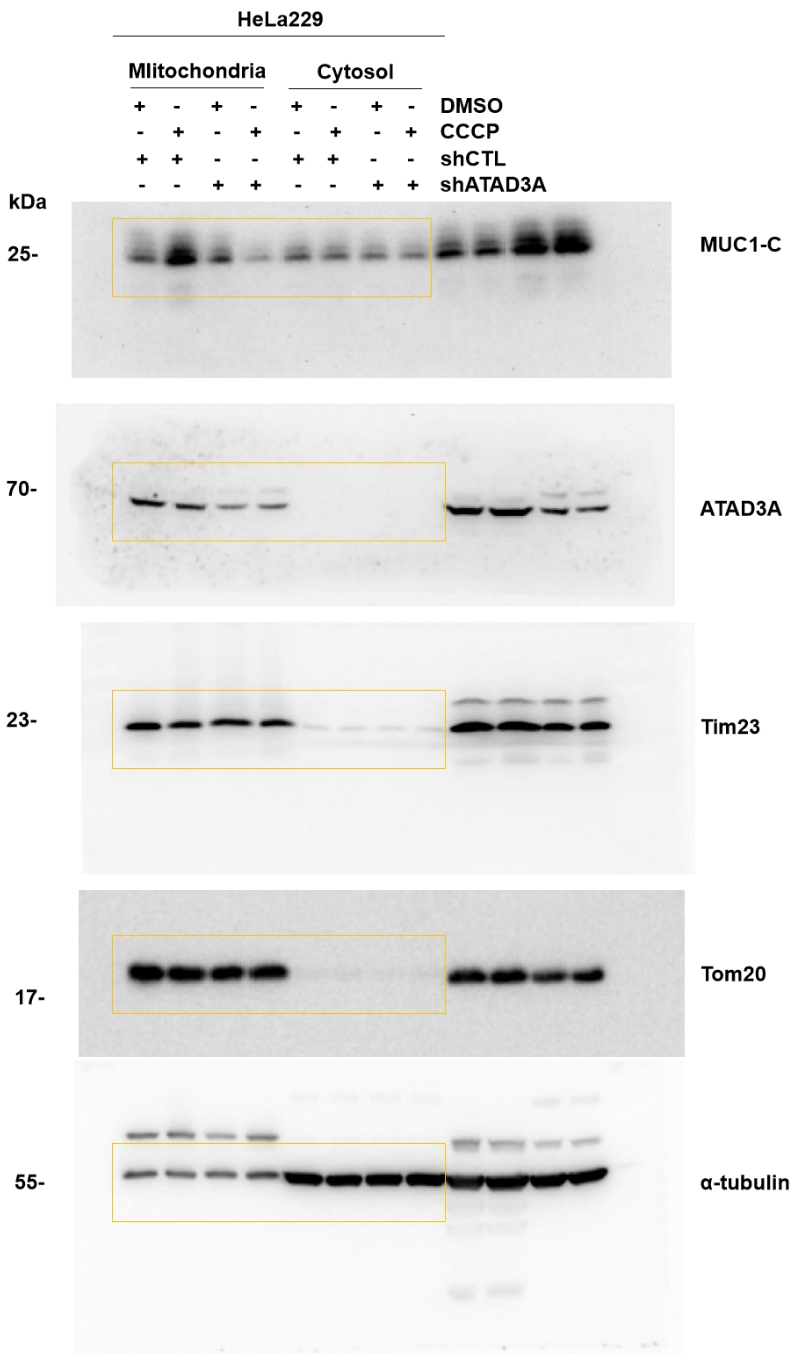
HeLa229
Mlitochondria
Cytosol
DMSO
CCCP
shCTL
shATAD3A
kDa
25-
70-
23-
17-
55-
MUC1-C
ATAD3A
Tim23
Tom20
α-tubulin

Figure 6

Fig. 6A

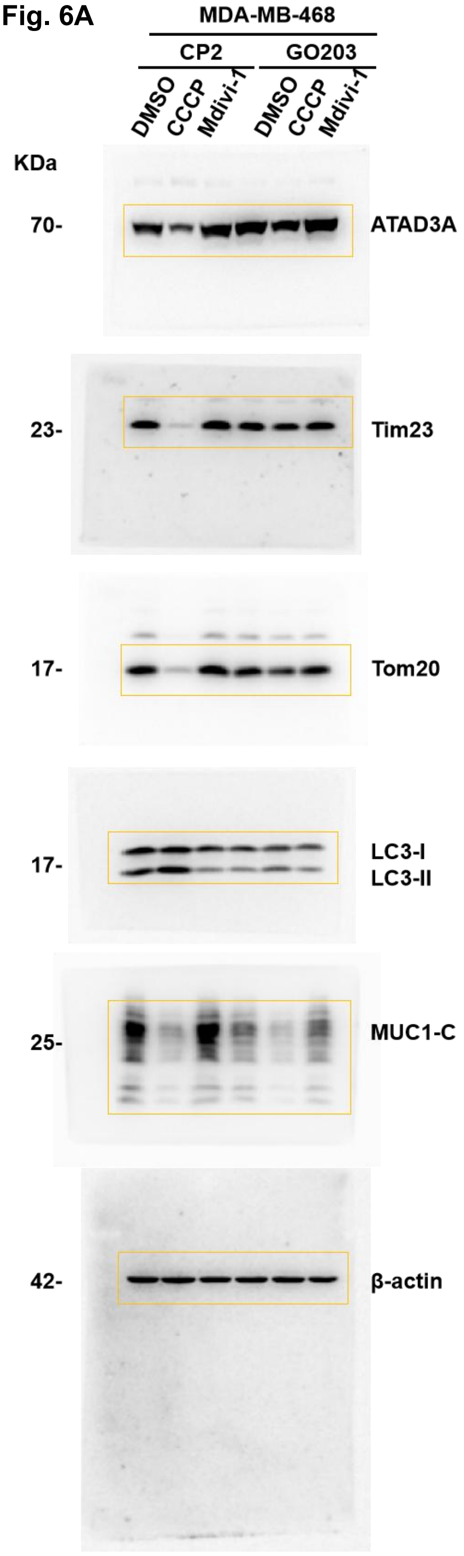


Fig. 6B

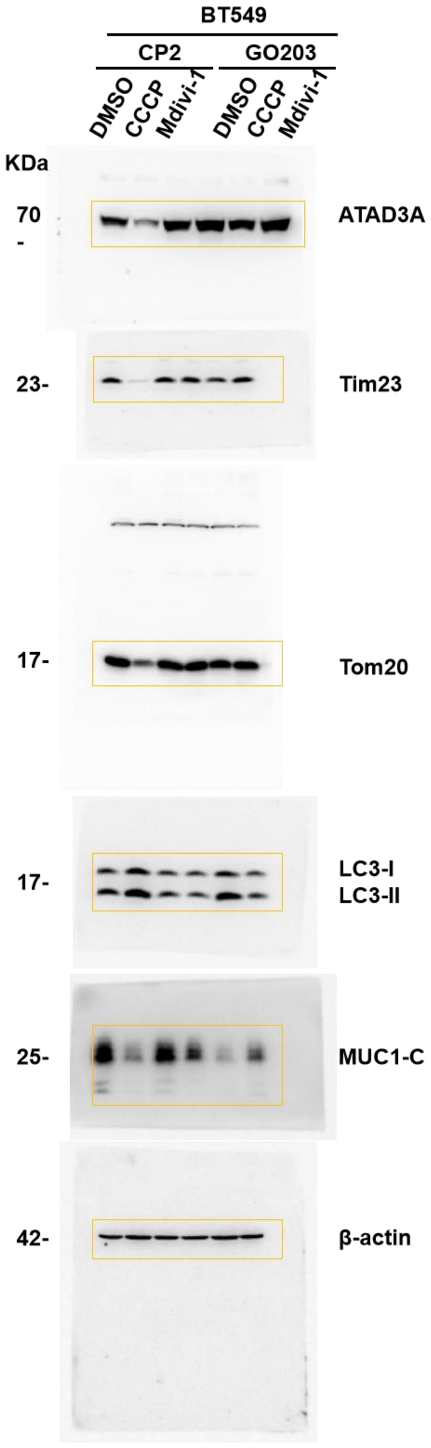

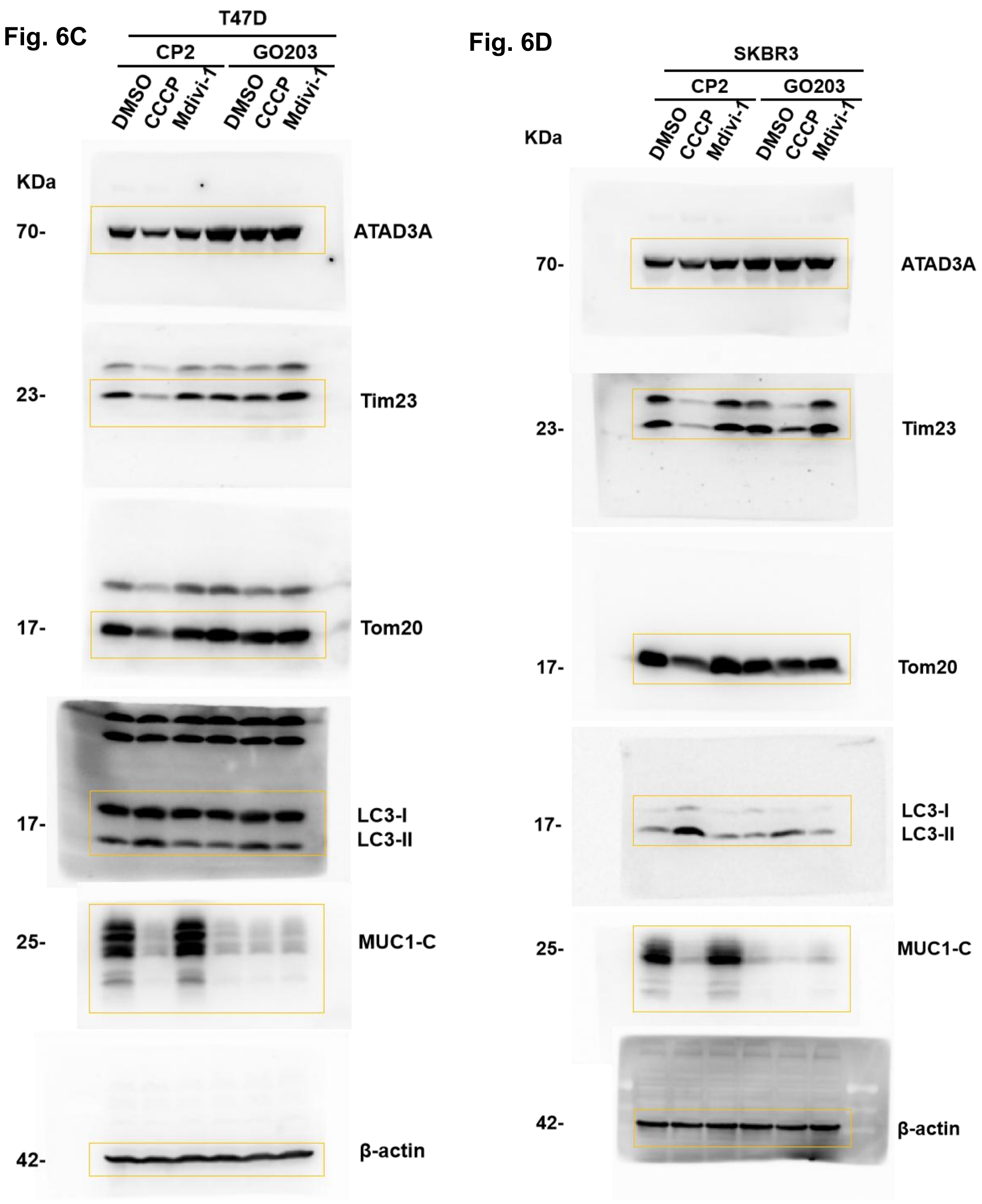
Fig. 6C
T47D
CP2
GO203
DMSO
CCCP
Mdivi-1
DMSO
CCCP
Mdivi-1
KDa
70-
ATAD3A
23-
Tim23
17-
Tom20
17-
LC3-I
LC3-II
25-
MUC1-C
42-
β-actin
Fig. 6D
SKBR3
CP2
GO203
KDa
DMSO
CCCP
Mdivi-1
DMSO
CCCP
Mdivi-1
70-
ATAD3A
23-
Tim23
17-
Tom20
17-
LC3-I
LC3-II
25-
MUC1-C
42-
β-actin

Figure S1

Fig. S1F

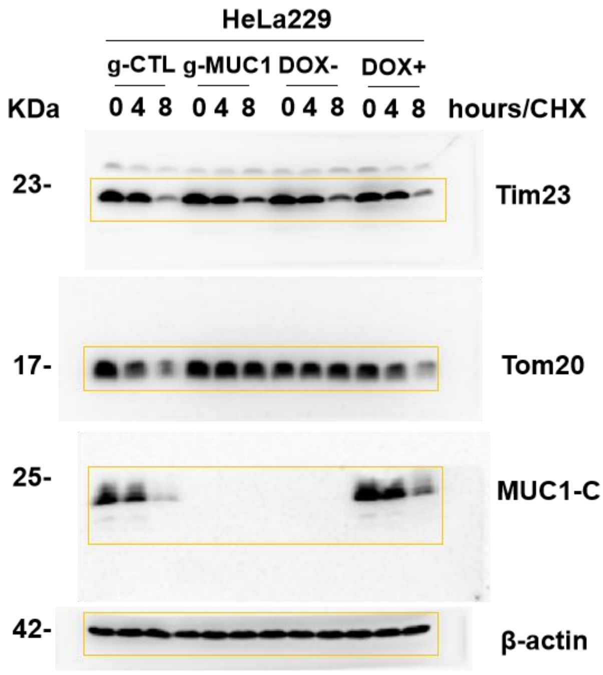


Fig. S1G

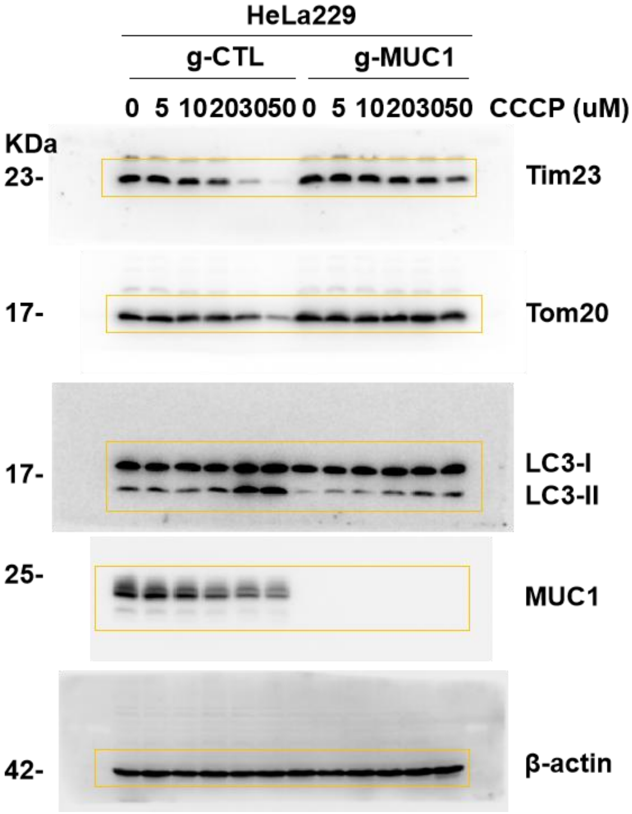

Fig. S1H

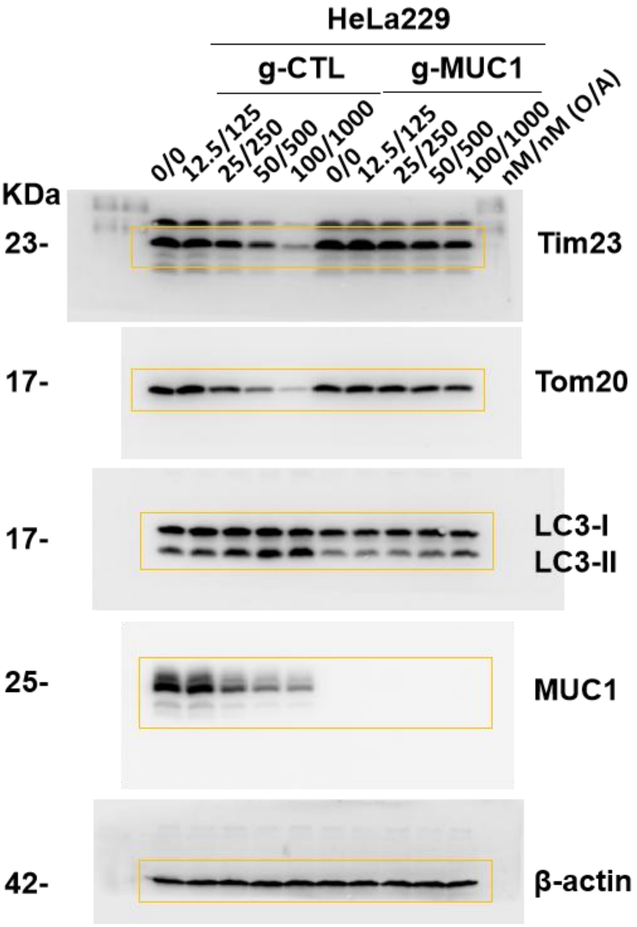
HeLa229
g-CTL
g-MUC1
0/0
12.5/125
25/250
50/500
100/1000
0/0
12.5/125
25/250
50/500
100/1000
nM/nM (O/A)
KDa
23-
Tim23
17-
Tom20
17-
LC3-I
LC3-II
25-
MUC1
42-
β-actin

Figure S3

Fig. S3D

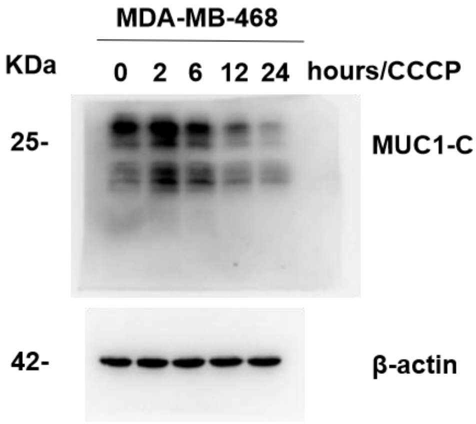


Fig. S3E

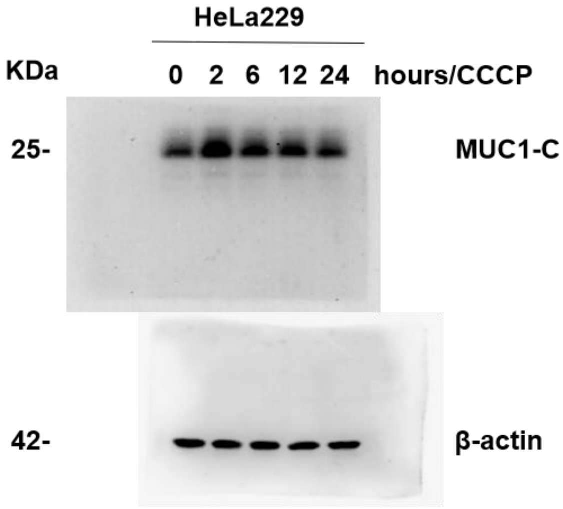

**Figure S4**

**Fig. S4B**

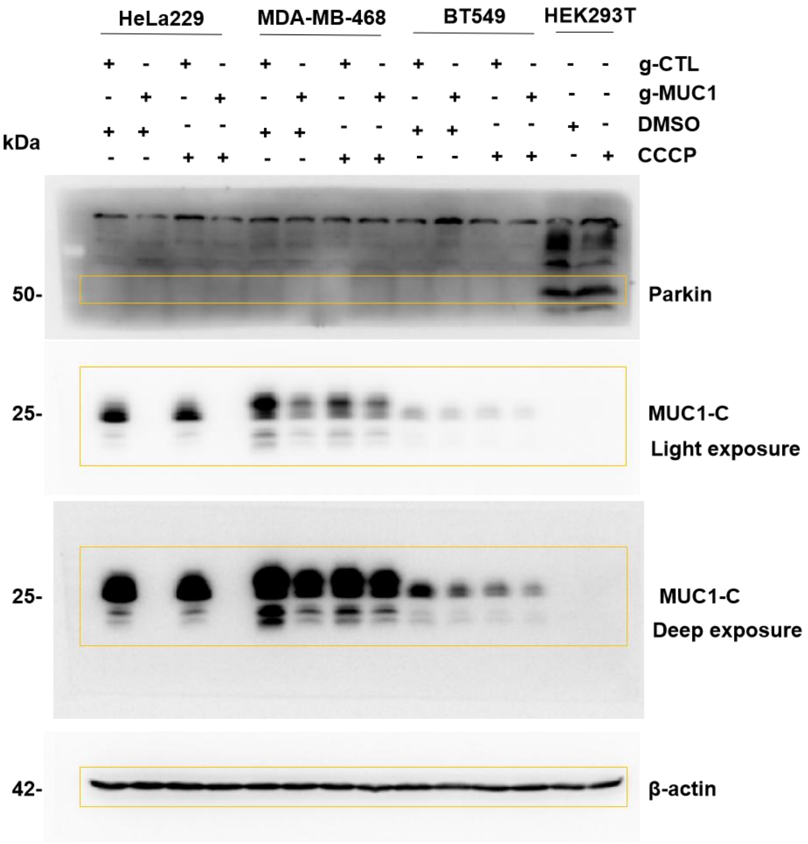

Fig. S4C

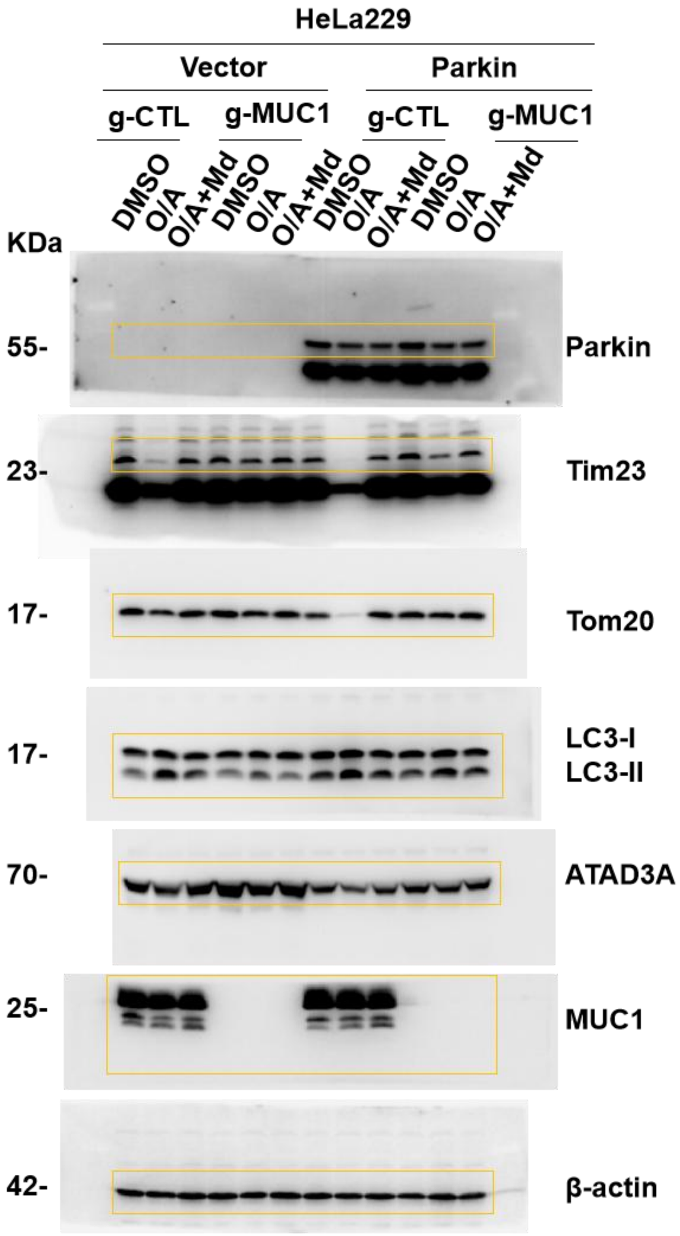
HeLa229
Vector
Parkin
g-CTL
g-MUC1
g-CTL
g-MUC1
DMSO
O/A
O/A+Md
DMSO
O/A
O/A+Md
DMSO
O/A
O/A+Md
DMSO
O/A
O/A+Md
KDa
55-
Parkin
23-
Tim23
17-
Tom20
17-
LC3-I
LC3-II
70-
ATAD3A
25-
MUC1
42-
β-actin

**Fig. S4D**

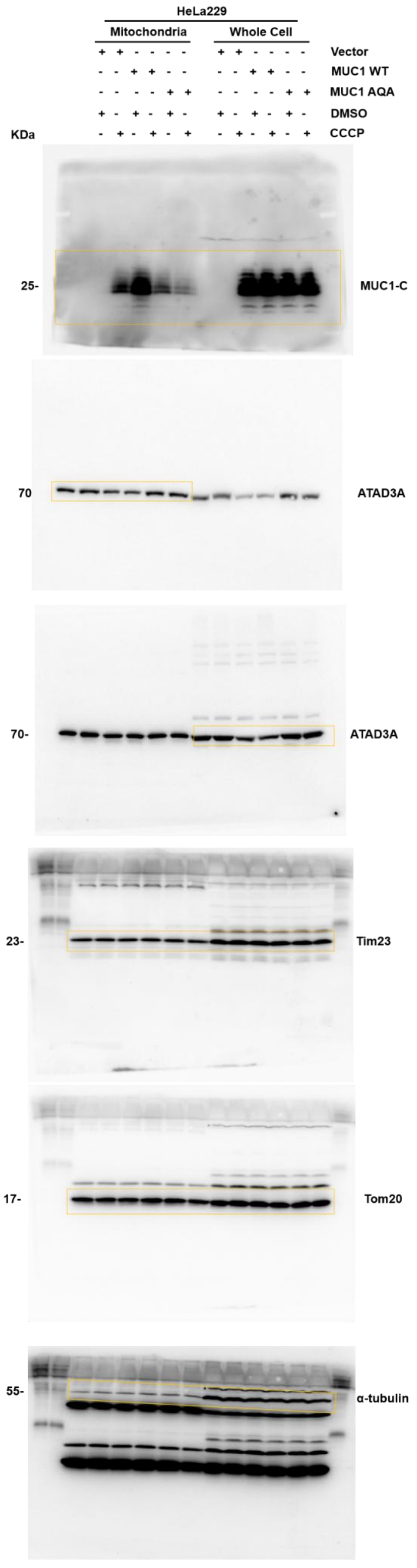
HeLa229
Mitochondria
Whole Cell
Vector
MUC1 WT
MUC1 AQA
DMSO
CCCP
KDa
25-
MUC1-C
70
ATAD3A
70-
ATAD3A
23-
Tim23
17-
Tom20
55-
α-tubulin

Fig. S4E

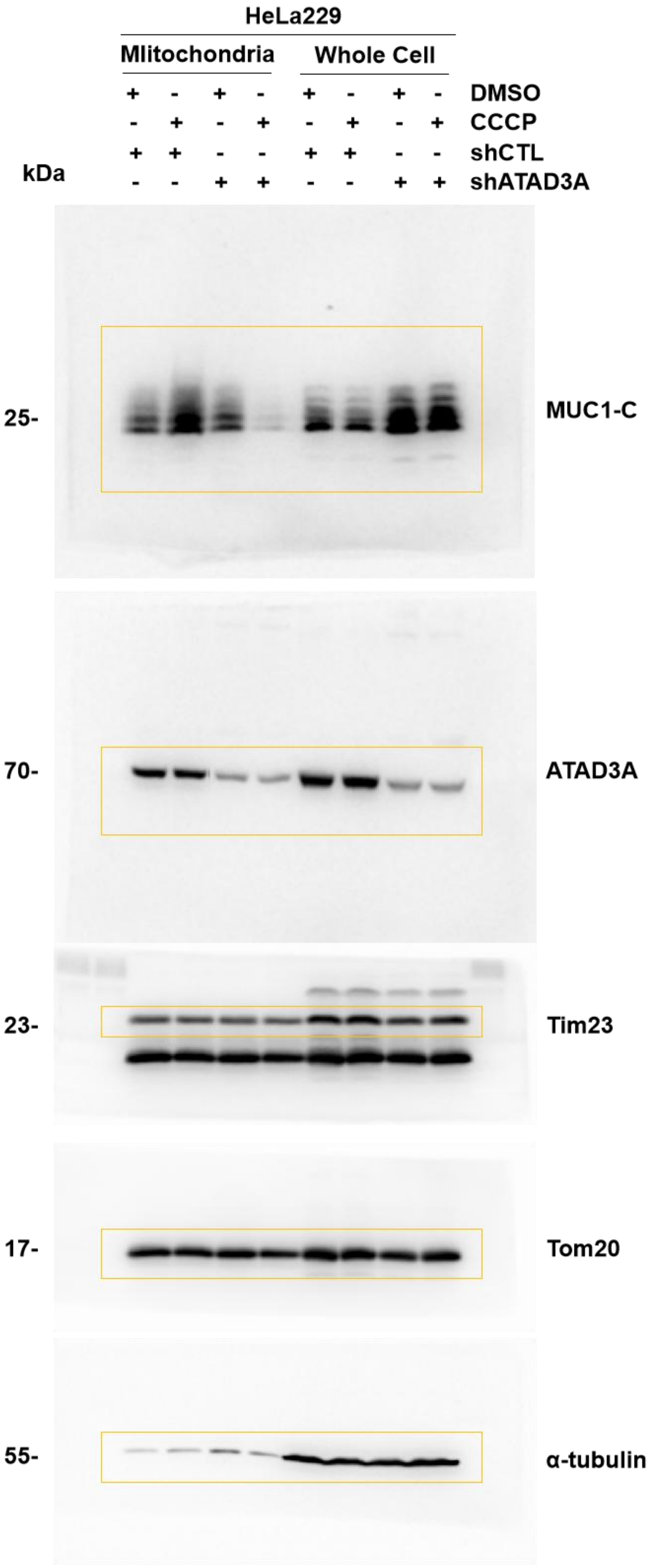

HeLa229
Mlitochondria
Whole Cell
+ - + - + - + - DMSO
- + - + - + - + CCCP
+ + - - + + - - shCTL
- - + + - - + + shATAD3A
kDa
25-
MUC1-C
70-
ATAD3A
23-
Tim23
17-
Tom20
55-
α-tubulin

**Figure S6**

**Fig. S6G**

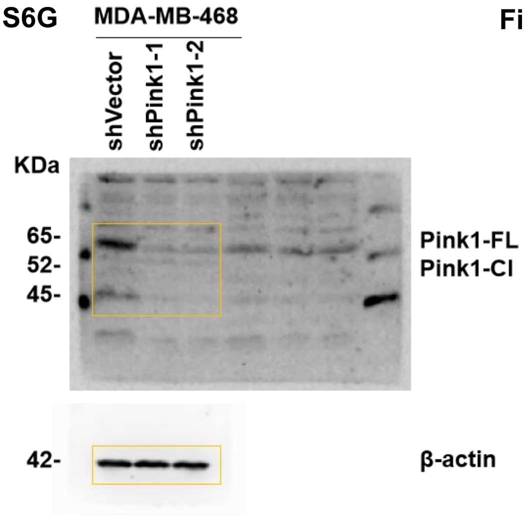


**Fig. S6H**

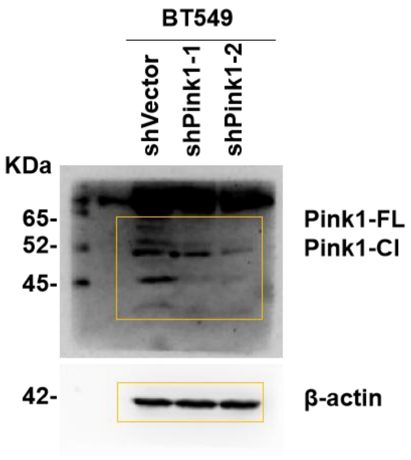

Supplement: Supplementary file 2 — Original western blots [file 41419_2022_5345_MOESM2_ESM.pdf]
